# Ionic–Bionic Interfaces: Advancing Iontronic Strategies for Bioelectronic Sensing and Therapy

**DOI:** 10.1002/advs.202513985

**Published:** 2025-11-19

**Authors:** Yun Goo Ro, Yoojin Chang, Jeeyoon Kim, Seungjae Lee, Sangyun Na, Cheolhong Park, Hyunhyub Ko

**Affiliations:** ^1^ School of Energy and Chemical Engineering Ulsan National Institute of Science and Technology (UNIST) Ulsan 44919 Republic of Korea

**Keywords:** bioelectronic interface, biosensing, Ionic materials, iontronics, therapeutics, tissue interfacing

## Abstract

Iontronic bioelectronics provides a powerful framework for bridging the mismatch between conventional electronic systems and soft, ion‐mediated biological tissues. By harnessing mobile ions as charge carriers and functional mediators, iontronic devices enable biocompatible, conformal, and low‐impedance interfaces that support both signal acquisition and therapeutic delivery. Recent advances in ionic materials, such as hydrogels, ion gels, and ionic liquids, have facilitated high‐fidelity physiological sensing, wound monitoring, and programmable drug and ion release. In addition to passive sensing and delivery, emerging iontronic platforms integrate real‐time biosignal monitoring with adaptive, AI‐guided feedback to enable closed‐loop therapeutic control. This review highlights the multifunctional role of ions in sensing, modulation, and stimulation across diverse applications, including skin‐interfaced electronics, neural and cardiac interfaces, and wound therapy. Key challenges such as operational stability, signal specificity, and long‐term biocompatibility are further examined, and material, structural, and system‐level innovations that are paving the way toward intelligent, responsive, and clinically viable iontronic bioelectronic platforms are discussed.

## Graphical abstract

Ionic–bionic interfaces for bioelectronics leverage ions as multifunctional mediators that combine mechanical compliance, ionic and electronic functionalities, and therapeutic effects. These systems offer real‐time biosignal transduction, effective wound dressing, responsive drug delivery, and seamless interaction between soft tissues and electronic platforms for advanced diagnostic and therapeutic applications.

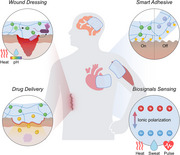

## 1 Introduction

Bioelectronics has emerged as a foundational technology in modern healthcare, driven by the shift toward personalized healthcare,^[^ ^1^, ^2^ ^]^ continuous physiological monitoring,^[^ ^3^ ^]^ and human–machine interfaces (HMIs).^[^ ^4^, ^5^ ^]^ Through direct communication between electronic systems and biological tissues, bioelectronics supports a wide range of applications, including diagnostics,^[^ ^6^ ^]^ neuromodulation,^[^ ^7^ ^]^ and targeted therapeutic delivery.^[^ ^8^ ^]^ A fundamental challenge in this field is achieving effective biointerfacing, the seamless structural and functional integration of electronics with soft, ion‐mediated biological tissues. Despite significant advances, many bioelectronic platforms still rely on rigid, electron‐based components, which are mismatched with the soft, dynamic, and ion‐mediated nature of biological environments.^[^ ^9^ ^]^ This mismatch often leads to poor interfacial adhesion, limited biocompatibility, and degraded signal transduction at the interface.^[^ ^10^ ^]^ Recent progress in soft electronics has mitigated mechanical disparities by introducing flexible and stretchable materials that better conform to tissues.^[^ ^11^ ^]^ However, these platforms still face limitations in interfacing with the ionic communication used by biological systems. While biological systems rely on ionic charge carriers to transmit signals and regulate physiological functions, traditional electronics rely on electron transport. This mismatch in charge carriers creates a communication bottleneck at the biointerface, limiting both the efficiency and fidelity of signal exchange across the biointerface.^[^ ^12^ ^]^

**Figure 1:**
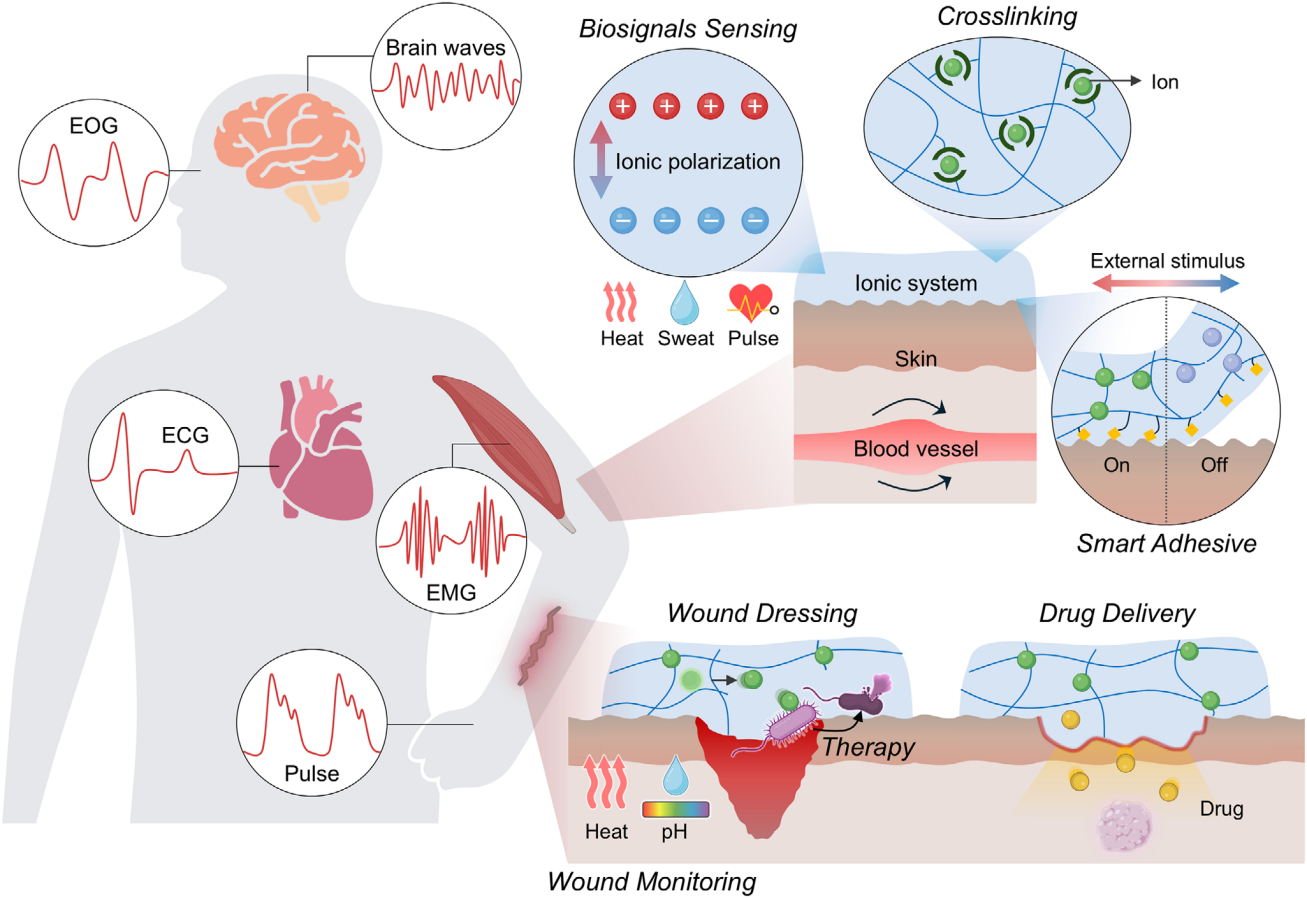
Overview of ionic–bionic interfaces for bioelectronics. Schematic illustration shows multifunctional applications of ionic bioelectronics interfaced with the human body. Upon skin attachment, ionic polarization and ion transport facilitate dynamic sensing, including real‐time biosignal acquisition, while ion‐mediated crosslinking supports adaptive adhesion. These systems further extend to therapeutic applications, including wound monitoring through pH and temperature sensing, wound dressing with antibacterial action, and controllable drug delivery. Part of the schematics were created using BioRender.com.

To overcome the charge carrier mismatch at the biointerface, the field has progressively moved toward iontronic bioelectronics—systems that incorporate ionic materials or iontronic devices to bridge biological and electronic signaling (**Figure** **1**). Iontronics has emerged as a promising paradigm in both electronics^[^ ^13^ ^]^ and materials science,^[^ ^14^ ^]^ with ionic systems enabling advances in sensors,^[^ ^15^, ^16^ ^]^ transistors,^[^ ^17^ ^]^ energy harvesters,^[^ ^18^ ^]^ and energy storage.^[^ ^19^ ^]^ As the field expands into bioelectronic applications, iontronics offers intrinsic compatibility with biological systems by leveraging ionic charge carriers and integrating electronic functionality, positioning ionic–bionic interfaces as a versatile platform for multifunctional bioelectronics.^[^ ^20^ ^]^ Systems incorporating ionic materials facilitate direct coupling with biological ion fluxes, supporting ionic signal transduction that closely mimics native ionic signaling pathways.^[^ ^21^ ^]^ In particular, ionic materials and iontronic devices have demonstrated enhanced performance in electrophysiological sensing, including electrocardiogram (ECG), electromyogram (EMG), electrooculogram (EOG), as well as brain wave monitoring, by reducing interfacial impedance and improving signal fidelity at the tissue–device interface.^[^ ^22^ ^]^ Ionic systems also provide additional advantages through their compositional and structural diversity. Each ionic species possesses distinct physicochemical parameters such as ionic radius, diffusivity, ion–polymer coordination strength, and polarization. Modulating these factors enables precise tuning of ionic conductivity and interfacial coupling, thereby controlling response time and sensitivity in sensing processes. Ionic polarization, in particular, governs the formation and modulation of electric double layers (EDLs) and internal dipole alignments that influence potential distribution and signal amplification at the sensing interface.^[^ ^23^ ^]^ This tunability allows ionic systems to achieve highly sensitive and selective transduction of chemical, biological, and physical stimuli, which has advanced the development of iontronic sensors for monitoring vital signs such as pulse waves,^[^ ^24^, ^25^ ^]^ respiratory activity,^[^ ^26^, ^27^ ^]^ and skin temperature,^[^ ^28^, ^29^ ^]^ broadening their role in multimodal physiological monitoring. Moreover, organic electrochemical transistors (OECTs), which use ionic electrolytes to convert biochemical interactions into amplified electrical signals, are increasing employed in biomarker sensing.^[^ ^30^ ^]^

In addition to their sensing functionality, ionic interactions can dynamically modulate polymer networks through dynamic crosslinking, imparting properties such as self‐healing, tunable stiffness, and mechanical adaptability, which are essential for maintaining intimate, long‐term contact with soft biological tissues.^[^ ^31^ ^]^ These properties support the performance of bioadhesives and epidermal electrodes, by providing stable and conformal interfaces with the skin^[^ ^32^ ^]^ and internal organs.^[^ ^33^ ^]^ In more advanced systems, reversible ion coordination or crosslinking can enable tunable adhesion that responds actively to physiological or environmental cues, offering programmable, adaptive functionality.^[^ ^34^, ^35^ ^]^

Beyond structural roles, ions also serve therapeutic functions in wound care, contributing to antibacterial activity,^[^ ^36^ ^]^ modulation of inflammation,^[^ ^37^ ^]^ and tissue regeneration.^[^ ^38^ ^]^ They also play key roles in drug delivery^[^ ^39^, ^40^ ^]^ and electrostimulation.^[^ ^41^ ^]^ This ability to actively engage biological processes at the interface level distinguishes iontronic systems from conventional soft electronics, which offer mechanical conformity but lack dynamic and therapeutic functionality.

This review highlights the role of ionic materials and iontronic devices in forming seamless interfaces between electronic systems and biological environments. We begin by introducing representative ionic species, ion‐conductive polymers, and the underlying principles of ionic–bionic interfacing. Recent advances in iontronic bioelectronics are then summarized, including material innovations such as ion‐mediated crosslinked adhesives, and their integration into epidermal electrodes and sensors for monitoring electrophysiological signals, physical stimuli, and wound‐related biomarkers. These platforms have also been applied in therapeutic applications, including wound dressings, drug delivery, and electrostimulation. Finally, we outline current challenges in the field and discuss strategic directions to address these limitations and guide future advancements.

## 2 Ionic Materials and Iontronics

Biointerfaces represent dynamic junctions between biological tissues and electronic systems, where molecular interactions and ionic exchanges govern signal transduction and maintain electrochemical homeostasis. These interfaces can be broadly categorized into skin, organ, and wound environments, each presenting unique challenges in terms of structural compliance, wettability, environmental exposure, and functional requirements.

**Figure 2:**
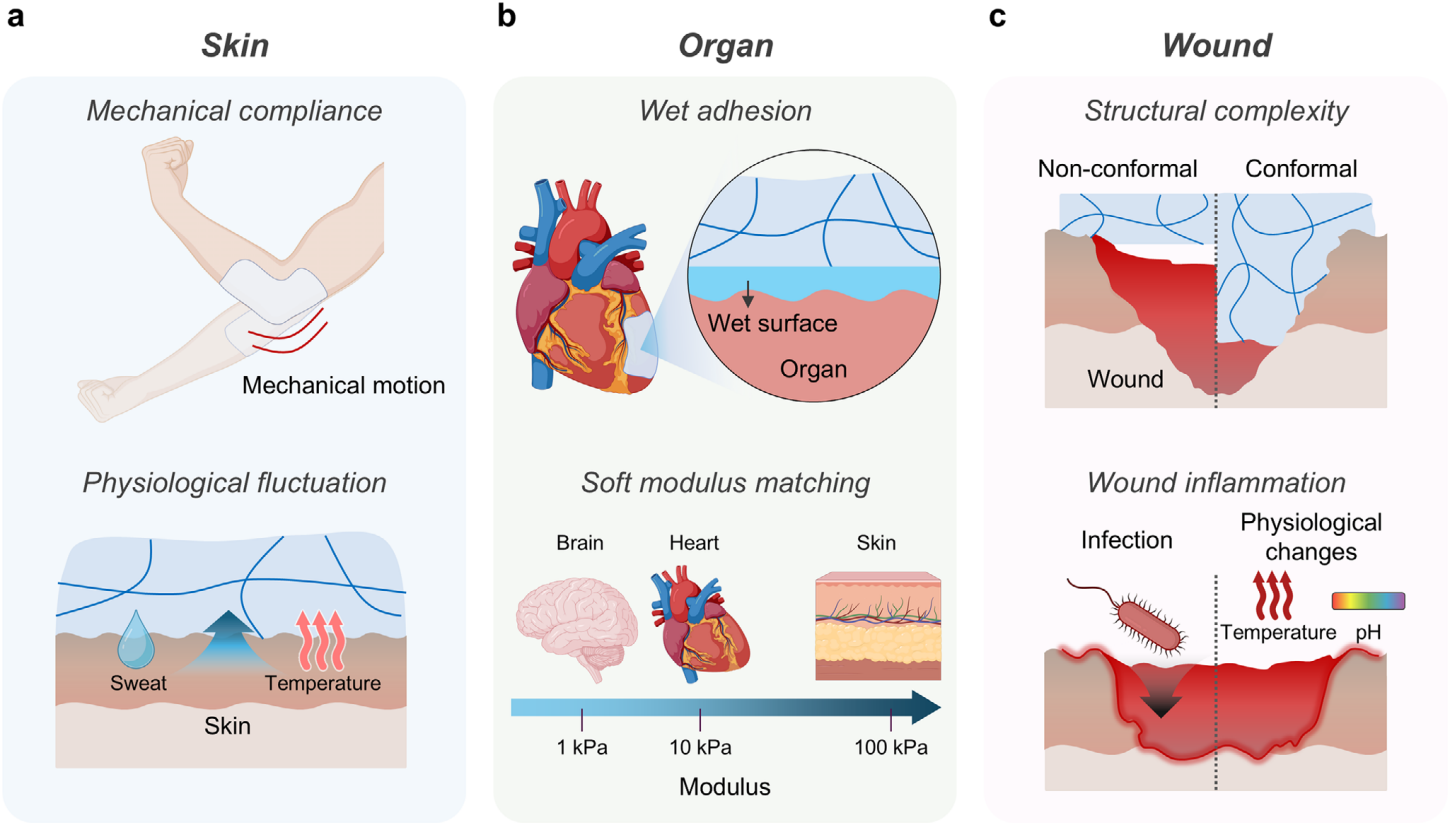
Representative biological interfaces and their associated design challenges for bioelectronic integration. a) Skin requires mechanical adaptability to joint motion and resilience to physiological variations including sweat and temperature. b) Organs demand strong wet adhesion and modulus matching to maintain reliable contact with dynamically moving soft tissues. c) Wounds exhibit structural irregularities and inflammatory fluctuations, where conformal coverage, antibacterial or antifouling properties, and responsiveness to biochemical cues (e.g., temperature, pH) are essential for therapeutic monitoring and healing. Schematics were created using BioRender.com.

Skin interfaces experience direct external exposure and continuous mechanical deformation (**Figure** **2**a). To ensure stable operation, devices must exhibit high mechanical compliance that accommodates large bodily movements while maintaining conformal contact. Additionally, epidermal electronics must preserve strong adhesion and electrical fidelity against skin‐derived byproducts such as sweat and temperature fluctuations, which can impair signal sensitivity and mechanical durability.

Organ interfaces operate in hydrated, physiologically dynamic environments rich in complex ionic and biochemical constituents (Figure 2b).^[^ ^42^ ^]^ Since internal organs possess a much lower elastic modulus than skin,^[^ ^9^ ^]^ devices for these conditions must employ low‐modulus materials to minimize interfacial stress and strain at tissue boundaries. Strong wet‐surface adhesion and long‐term mechanical stability are also essential to ensure continuous electrical performance and suppress immune responses during prolonged implantation.^[^ ^43^ ^]^

Wound interfaces require intimate, conformal contact with irregular and fragile surfaces while simultaneously serving as protective barriers against external contaminants (Figure 2c). Continuous exposure to microbial species and environmental pathogens demands both antibacterial and anti‐fouling functionalities. Moreover, wound healing activates immune responses that induce local physiological changes, such as elevated temperature and pH fluctuations.^[^ ^44^ ^]^ Therefore, integrating conformal adhesion with infection prevention, stability against local physiological variations, and the capability to monitor these changes enables the interface to actively support wound protection, healing, and real‐time monitoring without functional degradation.

Across all types of biointerfaces, ions serve as key design elements for enhancing interfacial integrity as they can tune mechanical properties,^[^ ^18^ ^]^ impart therapeutic functions,^[^ ^36^ ^]^ and enable sensing.^[^ ^45^ ^]^ While interfacial stability and adaptability can be optimized through the rational design of ionic species and matrix composition, the key challenge lies in balancing hydration stability, mechanical tunability, biocompatibility, and device performance by precisely controlling ionic species and their concentrations. A clear understanding of the physicochemical properties of ions is therefore essential for developing reliable and adaptive biointerfaces.

### 2.1 Ionic Species

#### 2.1.1 Native Ions

Ions play fundamental physiological functions in biological systems by mediating ion transport and electrostatic interactions with charged or partially charged biomolecules.^[^ ^46^, ^47^ ^]^ These interactions are central to maintaining homeostasis, contributing to key physiological processes, including neural signal transmission,^[^ ^48^ ^]^ osmotic balance,^[^ ^49^ ^]^ pH control,^[^ ^50^ ^]^ and cellular energy metabolism.^[^ ^51^ ^]^ In addition, ions mediate essential processes such as membrane potential generations,^[^ ^52^ ^]^ enzymatic activation,^[^ ^53^ ^]^ and protein conformation stabilization.^[^ ^54^ ^]^

Specific inorganic ions are essential for maintaining human health, as they participate in a wide range of physiological and biochemical functions. Their deficiency is closely associated with diseases, highlighting the importance of tightly regulated ionic homeostasis.^[^ ^55^ ^]^ Iron (Fe^3+^/Fe^2+^) in blood is essential for oxygen metabolism,^[^ ^56^ ^]^ while calcium (Ca^2+^) and phosphate (PO_4_^3−^) are fundamental to bone mineralization.^[^ ^57^ ^]^ Sodium (Na^+^), potassium (K^+^), and chloride (Cl^−^) regulate neural excitability and intracellular responses,^[^ ^58^, ^59^ ^]^ whereas proton (H^+^) and bicarbonate (HCO^3−^) maintain pH balance through buffering systems.^[^ ^59^ ^]^ Furthermore, magnesium (Mg^2+^),^[^ ^60^ ^]^ zinc (Zn^2+^),^[^ ^61^ ^]^ act as cofactors or reactants that facilitate enzymatic activity and metabolic processes by stabilizing reaction intermediates and products. Due to their high mobility and specific interactions with charged biomolecules, bioions are essential not only for core physiological functions but also for signal transduction and rapid responses to environmental stimuli, serving as indispensable elements in the function of excitable and adaptive systems such as nerves, muscles, and sensory organs.

### 2.2 Highly Mobile Ions

**Figure 3:**
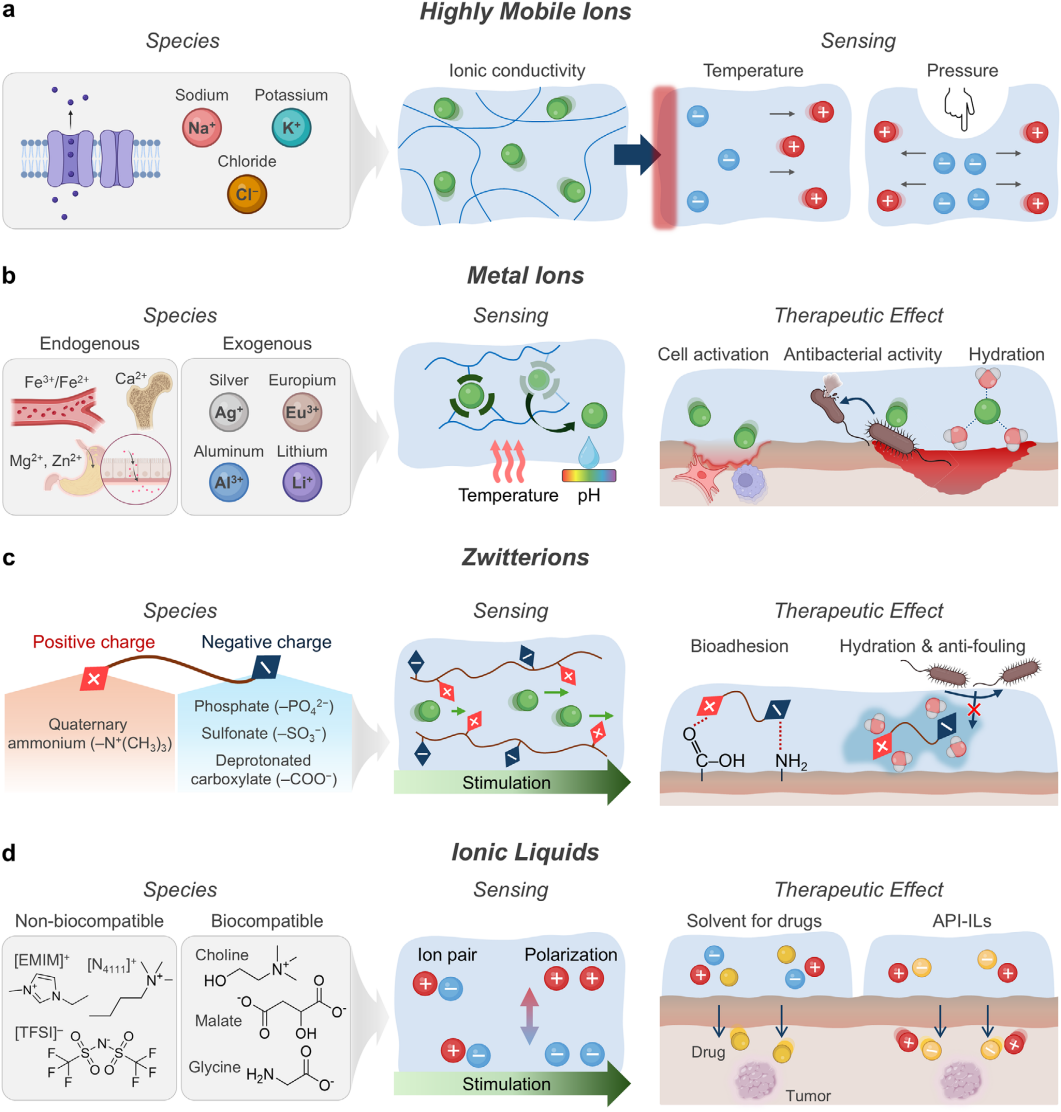
Ionic species. a) Highly mobile ions provide ionic conductivity in hydrogels, enabling sensing via charge separation in response to external stimuli such as temperature and pressure. b) Metal ions originated from endogenous and exogenous sources, mediating sensing through coordination dynamics under external stimuli and promoting therapeutic effects such as cell and tissue growth, antibacterial action, and enhanced hydration. c) Zwitterions with both positive and negative charges, altering ionic transport under stimulation and providing therapeutic benefits through bioadhesion, hydration, and antifouling properties. (d) Ionic liquids induce charge polarization under external stimulation for sensing applications and offering therapeutic utility as drug solvents and active pharmaceutical ingredients (API‐ILs). Part of the schematics were created using BioRender.com.

In bioelectronics, native ionic species are widely utilized for their ionic conductivity, water solubility, and excellent bio‐ and cyto‐compatibility. These properties make them ideal for incorporation into water‐based hydrogels, where mobile ions contribute to ionic conductivity^[^ ^62^ ^]^ Highly mobile ions such as Na^+^, K^+^, and Cl^−^ respond sensitively to diverse physiological and external stimuli, including temperature^[^ ^63^, ^64^ ^]^ and pressure,^[^ ^65^, ^66^ ^]^ enabling dynamic monitoring of local tissue environments (**Figure** **3**a).

### 2.3 Metal Ions

Metal ions such as Ca^2+^,^[^ ^67^ ^]^ Mg^2+^,^[^ ^68^ ^]^ Fe^3+^,^[^ ^69^ ^]^ Cu^2+^,^[^ ^70^ ^]^ and Zn^2+^,^[^ ^71^ ^]^ are increasingly used in soft bioelectronics, serving as mobile charge carriers and reinforcing polymer networks through coordination with functional ligands such as deprotonated carboxylate (–COO^−^)^[^ ^72^ ^]^ or bisphosphonate (Figure 3b).^[^ ^73^ ^]^ These coordination interactions enhance mechanical stability,^[^ ^74^, ^75^ ^]^ provide tunable stiffness,^[^ ^76^ ^]^ and enable self‐healing via dynamic bonds.^[^ ^77^ ^]^ Moreover, metal ion‐polymer systems exhibit stimuli‐responsive behavior, as changes in temperature modulate metal ion mobility and coordination strength,^[^ ^78^ ^]^ while pH affects functional group ionization and coordination density.^[^ ^79^ ^]^ Beyond structural roles, many metal ions provide therapeutic benefits through their intrinsic antibacterial properties, disrupting bacterial membranes, inactivating enzymes, and inducing oxidative stress.^[^ ^36^ ^]^ When released from hydrogels, these ions promote cellular responses such as cell migration, proliferation, and differentiation,^[^ ^80^ ^]^ facilitating endogenous activation with tissue,^[^ ^81^ ^]^ bone,^[^ ^82^, ^83^, ^84^, ^85^ ^]^ and cartilage^[^ ^71^ ^]^ regeneration. Notably, Mg^2+^ ions have been shown to activate vascular endothelial growth factor (VEGF) expression, promoting vascularization.^[^ ^86^, ^87^ ^]^ Furthermore, drugs with metal‐coordinating groups can reversibly complex with metal ions to form drug‐coordinated materials, enabling high drug loading, targeted delivery, and stimuli‐responsive release in pathological environments.^[^ ^40^ ^]^ Taken together, these features establish native ions as multifunctional components that support both biosensing and therapeutic functions in advanced bioelectronic systems.

#### 2.3.1 Exogenous Ions

The essential roles of native ions in bioelectronic systems have spurred growing interest in exogenous ionic materials, including non‐native metal ions, zwitterionic polymers, and ionic liquids. These materials not only replicate natural ionic functions but also offer additional capabilities, such as dynamic responsiveness to external stimuli and therapeutic delivery, thereby expanding the functional and design space of bioelectronic interfaces.

### 2.4 Non‐native Metal Ions

While biologically prevalent metal ions, such as Ca^2+^, Fe^3+^, and Mg^2+^ are favored for their biocompatibility, other non‐native metal ions such as silver (Ag^+^), aluminium (Al^3+^), europium, (Eu^3+^) and lithium (Li^+^) have also been utilized in bioelectronic applications. Ag^+^ is particularly notable for its strong antibacterial properties at low concentrations,^[^ ^88^, ^89^ ^]^ which has led to its wide application in wound dressings^[^ ^79^ ^]^ and wearable electronics.^[^ ^90^ ^]^ In addition, Ag^+^ forms strong coordination bonds with functional groups such as –COO^−[^ ^91^ ^]^ and thiol (–SH),^[^ ^92^ ^]^ driving the formation of mechanically robust polymer networks. Similarly, Al^3+^ engages in multivalent interactions with various negative charged groups such as –COO^−^ and phosphates (–PO_4_^2−^), enhancing mechanical strength and self‐healing in polymer matrices.^[^ ^93^ ^]^ It also exhibits antibacterial effects^[^ ^94^ ^]^ and promotes long‐term hydration in hydrogels by attracting water molecules through its high ionic charge density.^[^ ^95^ ^]^ Eu^3+^ and Li^+^ further support polymer coordination,^[^ ^96^, ^97^ ^]^ and hydration,^[^ ^98^, ^99^ ^]^ broadening applications in bioelectronics.^[^ ^100^, ^101^ ^]^

Beyond metallic ions, metalloid boron forms covalent bonds with oxides to generate borate ions (BO_3_^3−^ or B(OH)_4_^−^), which are attractive for bioelectronics due to their reversible dynamic bonding,^[^ ^102^ ^]^ particularly their ability to form dynamic boron–diol complexes with diol‐containing groups that enable self‐healing properties in polymer networks.^[^ ^103^, ^104^ ^]^ In addition, their pH‐ and temperature‐responsive coordination behavior facilitates the design of stimuli‐responsive hydrogels.^[^ ^105^, ^106^ ^]^ Moreover, the presence of free borate ions within the polymer matrix enhances ionic conductivity, supporting their application in strain‐sensing platforms.^[^ ^107^ ^]^

Metal ions offer broad functional versatility in bioelectronics, enabling functions such as coordination crosslinking, signal transduction, and antibacterial properties. However, excessive metal ion concentrations can disrupt cellular homeostasis and induce cytotoxic effects, underscoring the need for precise control over ion dosage to ensure biocompatibility.^[^ ^108^, ^109^ ^]^

### 2.5 Zwitterions

Zwitterions are molecules that contain both cationic and anionic functional groups, such as protonated amines (–NH_3_^+^) and carboxylates (–COO^−^), or sulfonates (–SO_3_^−^).^[^ ^110^ ^]^ While they are electrically neutral overall, their internal charge separation generates dipole moments that mediate local electrostatic interactions in biological environments.^[^ ^111^ ^]^ In biological systems, zwitterionic structures are prevalent. Amino acids, the building blocks of proteins, exist predominantly in zwitterionic structure at physiological pH (≈7.4).^[^ ^112^ ^]^ featuring protonated amine (–NH_3_^+^) and deprotonated carboxylate (–COO^−^) groups. These functional groups contribute to diverse physicochemical properties and enable complexation processes that improve the aqueous solubility of otherwise poorly water‐soluble species.^[^ ^112^ ^]^ Similarly, cell membrane phospholipids such as phosphatidylcholine contain zwitterionic headgroups comprising phosphate (–PO_4_^2−^) and choline (–N^+^(CH_3_)_3_) groups.^[^ ^113^ ^]^ These zwitterionic lipids preserve membrane integrity while mediating interactions with other zwitterionic molecules and proteins, supporting cellular polarity and membrane‐protein communication.^[^ ^114^ ^]^ In addition, zwitterionic phospholipids exhibit strong hydration, with water molecules forming stable hydrogen‐bonded networks around the phosphate group and more loosely associated clathrate‐like structures around the choline group.^[^ ^115^ ^]^ Collectively, these properties contribute to the structural, electrochemical, and functional integrity in biological systems.^[^ ^116^ ^]^

The pronounced hydrophilicity of zwitterions has spurred the integration of their polymeric forms into bioelectronic platforms^[^ ^117^, ^118^ ^]^ (Figure 3c). Among them, phosphorylcholine (PC), is commonly used in zwitterionic polymers for its biomimetic properties, as it mimics the headgroup of phospholipids found in cell outer membranes.^[^ ^119^ ^]^ Other zwitterionic moieties, such as sulfobetaine (SB)^[^ ^120^ ^]^ and carboxybetaine (CB),^[^ ^121^ ^]^ are widely employed in bioelectronics owing to their exceptional biocompatibility.^[^ ^122^ ^]^ Additional representative zwitterions used in bioelectronics platforms include [2‐(methacryloyloxy)ethyl] dimethyl‐(3‐sulfopropyl)ammonium hydroxide (DMAPS)^[^ ^123^, ^124^ ^]^ and trimethylamine N‐oxide (TMAO).^[^ ^125^, ^126^ ^]^

Zwitterionic matrices exhibit excellent antifouling properties by tightly binding water molecules through hydrogen bonding, forming a stable hydration layer that resists the adhesion of proteins, cells, and bacteria.^[^ ^116^, ^127^ ^]^ Their strong water‐retention capability improves resistance to dehydration, overcoming a major drawback of conventional hydrogels.^[^ ^128^ ^]^ Owing to their structural resemblance to natural skin and resistance to microbial contamination, zwitterion matrices are especially suitable for tissue‐mimicking scaffolds^[^ ^129^ ^]^ and protective barriers for infected or damaged skin.^[^ ^130^ ^]^ In addition, dipole–dipole interactions with nucleophilic groups (hydroxyl, amino, carboxyl, amide) groups on tissue surfaces enable the use of zwitterionic polymers as effective bioadhesives.^[^ ^120^, ^131^ ^]^ Beyond these roles, zwitterions are broadly applied in bioelectronics, including antifouling coating,^[^ ^125^, ^132^ ^]^ cell culture scaffolds,^[^ ^133^ ^]^ wound healing^[^ ^126^ ^]^ and drug delivery materials,^[^ ^134^ ^]^ tissue^[^ ^124^ ^]^ and bone^[^ ^123^ ^]^ regeneration platforms, and joint lubricants.^[^ ^135^ ^]^ Furthermore, zwitterions exhibit environment‐responsive behavior,^[^ ^136^ ^]^ leading to their use in biosensing by altering ionic transport properties in response to stimuli such as temperature,^[^ ^137^ ^]^ pH,^[^ ^138^ ^]^ and mechanical deformation.^[^ ^120^, ^128^, ^139^, ^140^ ^]^ As a result, zwitterionic systems show promise as both passive tissue interfaces and active therapeutic platforms that combine sensing, protection, and biofunctionality.

### 2.6 Ionic Liquids

Ionic liquids (ILs) are organic salts that remain liquid at or below 100 °C, often even at room temperature. They exhibit high ionic conductivity, non‐flammability, and low volatility, making them well‐suited for electrochemical and bioelectronic applications.^[^ ^141^ ^]^ ILs consist of various combinations of cations and anions (Figure 3d). Common cations include imidazolium‐based species such as 1‐ethyl‐3‐methylimidazolium ([EMIM]^+^)^[^ ^29^, ^142^ ^]^ 1‐butyl‐3‐methylimidazolium [BMIM]^+^,^[^ ^26^ ^]^ and ammonium‐based cations like N‐trimethyl‐N‐butylammonium ([N_4111_]^+^).^[^ ^143^ ^]^ Representative anions include bis(trifluoromethyl sulfonyl)imide ([TFSI]^−^),^[^ ^144^ ^]^ tetrafluoroborate ([BF_4_]^−^)^[^ ^145^ ^]^ and hexafluorophosphate ([PF_6_]^−^)^[^ ^146^ ^]^ While most ILs are hygroscopic, as their polar ionic structures strongly interact with water molecules,^[^ ^147^ ^]^ those containing fluorinated anions such as [TFSI]^−^ and [PF_6_]^−^ exhibit hydrophobic characteristics.^[^ ^148^ ^]^ Owing to their negligible vapor pressure, ILs are often incorporated into gel matrices to form ion gels, which are widely used in iontronic sensors leveraging EDL.^[^ ^15^ ^]^ They also serve as effective electrolytes in both liquid and gel forms for OECTs,^[^ ^149^ ^]^ where stable ion transport is critical for long‐term device performance.

While ILs offer outstanding electrochemical properties, imidazolium‐based ILs or those containing long alkyl chains suffer from limited biocompatibility due to their tendency to disrupt cellular membranes, interact with proteins, and resist biodegradation.^[^ ^150^ ^]^ Additionally, common anions such as [BF_4_]^−^ and [PF_6_]^−^ can hydrolyze in the presence of water, generating highly corrosive and toxic hydrogen fluoride.^[^ ^151^ ^]^ Likewise, [TFSI]^−^ has been shown to induce cellular toxicity by generating reactive oxygen spieces (ROS), leading to cell death.^[^ ^152^ ^]^ These toxicity concerns restrict the use of conventional ILs in biomedical applications.^[^ ^33^, ^153^ ^]^ To address these limitations, biocompatible and biodegradable ILs have been developed. Choline‐based cations, derived from natural sources, are among the most widely used biocompatible ILs.^[^ ^154^ ^]^ For anions, nature‐derived species such as amino acid‐derived glycine,^[^ ^155^ ^]^ as well as carboxylate‐based anions like acetate^[^ ^156^ ^]^ or lactate^[^ ^157^ ^]^ have been employed, retaining favorable physicochemical properties while minimizing cytotoxicity and improving biological compatibility. These anions not only reduce cytotoxicity but also offer functional groups for hydrogen bonding or ionic interactions, enhancing solubility, drug loading, and tissue compatibility.^[^ ^154^ ^]^ More recent developments of biocompatible anions include malate^[^ ^41^, ^158^, ^159^ ^]^ and fatty acid‐derived species such as oleate.^[^ ^160^ ^]^

In biomedical applications, ILs have been widely used as solvents to improve solubility and bioavailability of hydrophobic therapeutics^[^ ^161^, ^162^ ^]^ such as paclitaxel,^[^ ^163^ ^]^ curcumin,^[^ ^164^ ^]^ amphotericin B,^[^ ^165^ ^]^ and minoxidil.^[^ ^166^ ^]^ Moreover, ILs can disrupt lipid barriers and penetrate the stratum corneum barriers,^[^ ^167^, ^168^ ^]^ making them effective vehicles for transdermal and topical drug delivery. Notably, certain ILs can function as active pharmaceutical ingredients (API‐ILs) by incorporating therapeutic agents directly into the IL structure.^[^ ^161^, ^169^, ^170^ ^]^ Representative examples include ibuprofen,^[^ ^171^ ^]^ lidocaine,^[^ ^172^ ^]^ and salicylate for pain relief.^[^ ^173^ ^]^ Moreover, their wide electrochemical window expands their utility to neuromodulation.^[^ ^41^, ^174^ ^]^ Owing to their unique physicochemical characteristics, ILs serve as ionic conductors, drug solubilizers, and therapeutic agents, broadening their applicability in bioelectronic interfaces and biomedical therapeutics.

The integration of native and exogenous ions provides a versatile platform for designing multifunctional bioelectronic systems (**Table** **1**). Native ions exhibit intrinsic biocompatibility and can be embedded in polymer matrices to provide ionic conductivity and responsiveness to external stimuli for sensing. Metal ions coordinate with polymers to improve mechanical strength and introduce dynamic crosslinking that supports self‐healing and stretchability. Metal ions also play active biological roles by exhibiting antimicrobial, anti‐inflammatory, and regenerative effects. Zwitterionic species offer antifouling through strong hydration, enhancing biostability. ILs function as sensing media, drug carriers, or active therapeutic components. These ionic species collectively contribute both sensing and therapeutic functions. The key challenge remains precise control over these roles while maintaining long‐term safety, degradability, and biological compatibility.

**Table 1:** Ionic species.

Category	Type	Ionic species	Biocompatibility	Sensing mechanism	Therapeutic property	Additional property	Applications
Endogenous ions	Highly mobile ions	Na^+^, K^+^, Cl^−^	High	Stimuli dependent ion migration and charge separation	‐	‐	Physiological sensor
	Transition metal ions	Ca^2+^, Fe^3+^/Fe^2+^, Mg^2+^, Zn^2+^, Cu^2+^	Toxic at high concentration	Coordination dynamics	Anti‐bacterial, anti‐dehydration, tissue regeneration	Self‐healing capability	Wound therapy, bio‐adhesive, biosensor
Exogenous ions	Non‐native metal ions	Metal ions: Ag^+^, Al^3+^, Li^+^, Eu^3+^ Metalloid: borate ions (BO_3_^3−^ or B(OH)_4_^−^)	Toxic at high concentration				
	Zwitterions	PC, SB, CB, DMAPS, TMAO	High	Ionic conduction/transport		Self‐healing capability, adhesion	
	ILs	Cation: [EMIM]^+^, [BMIM]^+^, [N_4111_]^+^, choline Anion: [TFSI]^−^, [BF_4_]^−^, [PF_6_]^−^, glycine, acetate, lactate, malate, oleate	Non‐biocompatible: imidazolium‐based, long alkyl chain, sulfonylimide‐based ILs Biocompatible: choline‐based, amino acid‐derived, carboxylate‐based ILs	Stimuli dependent ion migration and charge separation	Drug solvents, API‐ILs	Skin stratum corneum barrier penetration	Physiological sensor, OECT, drug delivery, API‐IL

### 2.7 Ionic–Bionic Interfaces

**Figure 4:**
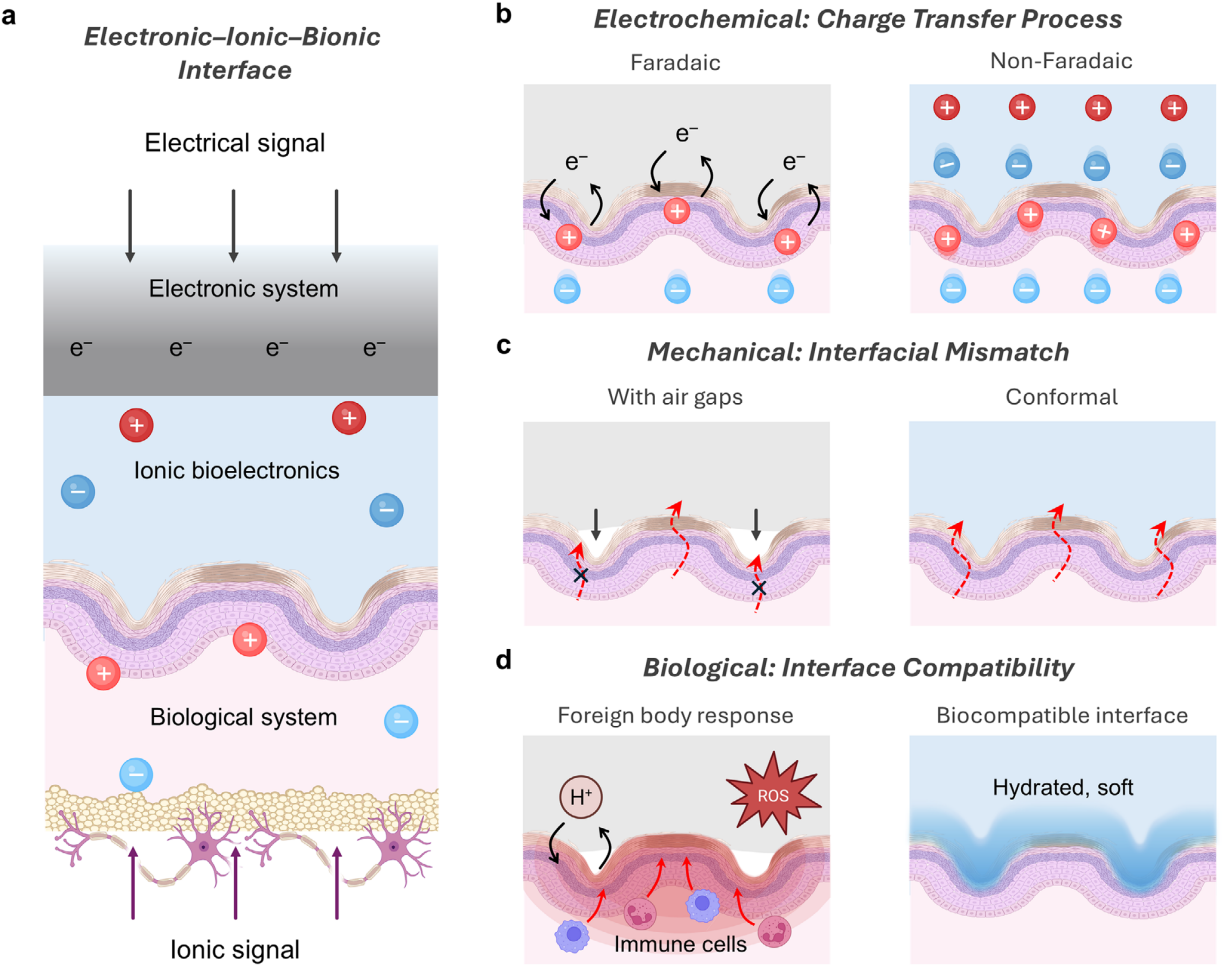
Ionic bioelectronics interfacing with biological systems. a) Ionic bioelectronics bridging biological and electronic systems, enabling efficient signal transduction. b) Charge transfer mechanisms at the ionic bioelectronics–tissue interface. Faradaic processes involve redox reactions at the interface (left), while non‐Faradaic processes rely on capacitive ion accumulation without electron transfer (right). c) Mechanical mismatch at the ionic bioelectronics–tissue interface. Mechanical mismatch induces air gaps at the interface, resulting in high mechanical impedance (left), whereas soft polymer matrices achieve conformal contact with tissues, reducing mechanical impedance (right). d) Foreign materials trigger immune responses that produce reactive oxygen species (ROS) and local pH fluctuations, leading to inflammation and tissue damage (left). In contrast, biocompatible interfaces with hydrated and soft contact minimize immune activation and maintain interfacial stability (right). Part of the schematics were created using BioRender.com.

Effective bioelectronic systems require creating interfaces that can seamlessly interact with biological tissues, which transmit signals through ionic pathways and possess soft, flexible architectures. The essential challenge is to achieve reliable electrical and mechanical coupling while ensuring biocompatibility. Yet, traditional rigid electronic interfaces struggle with electrical and mechanical mismatches and limited biological tolerance. Establishing a stable and biocompatible interface that can mediate signal exchange across these mismatched interfaces is essential, as poor interfacial compatibility can lead to signal loss, increased impedance,^[^ ^175^ ^]^ and eventual device failure in long‐term applications.^[^ ^176^ ^]^ Biological tissues transmit signals primarily through ionic charge carriers via action potentials, synaptic transmission, and ion channel gating.^[^ ^177^ ^]^ In contrast, conventional electronic devices rely on electronic carriers (electrons and holes), resulting in an inherent electronic and ionic mismatch at the interface.^[^ ^178^ ^]^ Effective signal transduction thus requires conversion of ionic to electronic signals,^[^ ^179^ ^]^ typically achieved through ionic materials or iontronic devices that facilitate intimate coupling with tissue interfaces, enabling bidirectional communication (**Figure** **4**a). This signal transduction is governed by Faradaic or non‐Faradaic electrochemical processes.^[^ ^180^ ^]^ Faradaic charge transfer involves redox reactions between the electrode and electroactive species (Figure 4b). However, faradaic process primarily involves irreversible redox reactions, such as water electrolysis and ROS formation, which can damage both electrodes and surrounding tissues.^[^ ^181^ ^]^ In contrast, non‐Faradaic process relies on ion accumulation without chemical reactions, achieving efficient capacitive coupling via EDL formation, thereby reducing impedance and improving signal fidelity.^[^ ^182^ ^]^

Beyond electrical impedance, mechanical mismatch remains a critical barrier to stable bioelectronic interfacing^[^ ^183^, ^184^ ^]^ (Figure 4c). Most human tissues are soft and hydrated, with Young's moduli below 100 kPa, whereas conventional electrode materials such as metal, ceramic, and silicon are dry and rigid, with moduli exceeding 1 GPa.^[^ ^185^, ^186^ ^]^ This mechanical difference hinders conformal and stable contact at the tissue–electrode interface. Moreover, biological tissues undergo dynamic deformation (e.g., stretching, bending), making it particularly difficult for rigid devices to maintain stable adhesion.^[^ ^187^ ^]^ Mechanical mismatches in modulus and stretchability can lead to interfacial delamination, mechanical failure, and disrupted electrical continuity, ultimately degrading signal quality. To address this issue, recent efforts have focused on the development of soft polymer‐based electrode matrices that better match the mechanical properties of biological tissues.^[^ ^16^ ^]^ Addressing both mechanical and electrical impedance mismatches is essential for the reliable operation of bioelectronic devices. Materials that combine mechanical softness with embedded ionic species represent a promising solution for building stable tissue–electrode interfaces.^[^ ^188^ ^]^

From a biological standpoint, maintaining interface compatibility is essential (Figure 4d). Non‐biocompatible materials are recognized as foreign substances, triggering immune responses that cause local pH fluctuations, cytotoxic effects, and the generation of ROS from inflammatory byproducts.^[^ ^189^ ^]^ Moreover, inadequate mechanical conformity between the device and surrounding tissue introduces interfacial stress, further irritating tissues and amplifying the immune response. Therefore, designing interfaces that combine soft, tissue‐like mechanics with high biocompatibility is vital for minimizing immune responses and ensuring long‐term interfacial stability.

#### 2.7.1 Organic Mixed Ionic‐Electronic Conductors (OMIEC)

**Figure 5:**
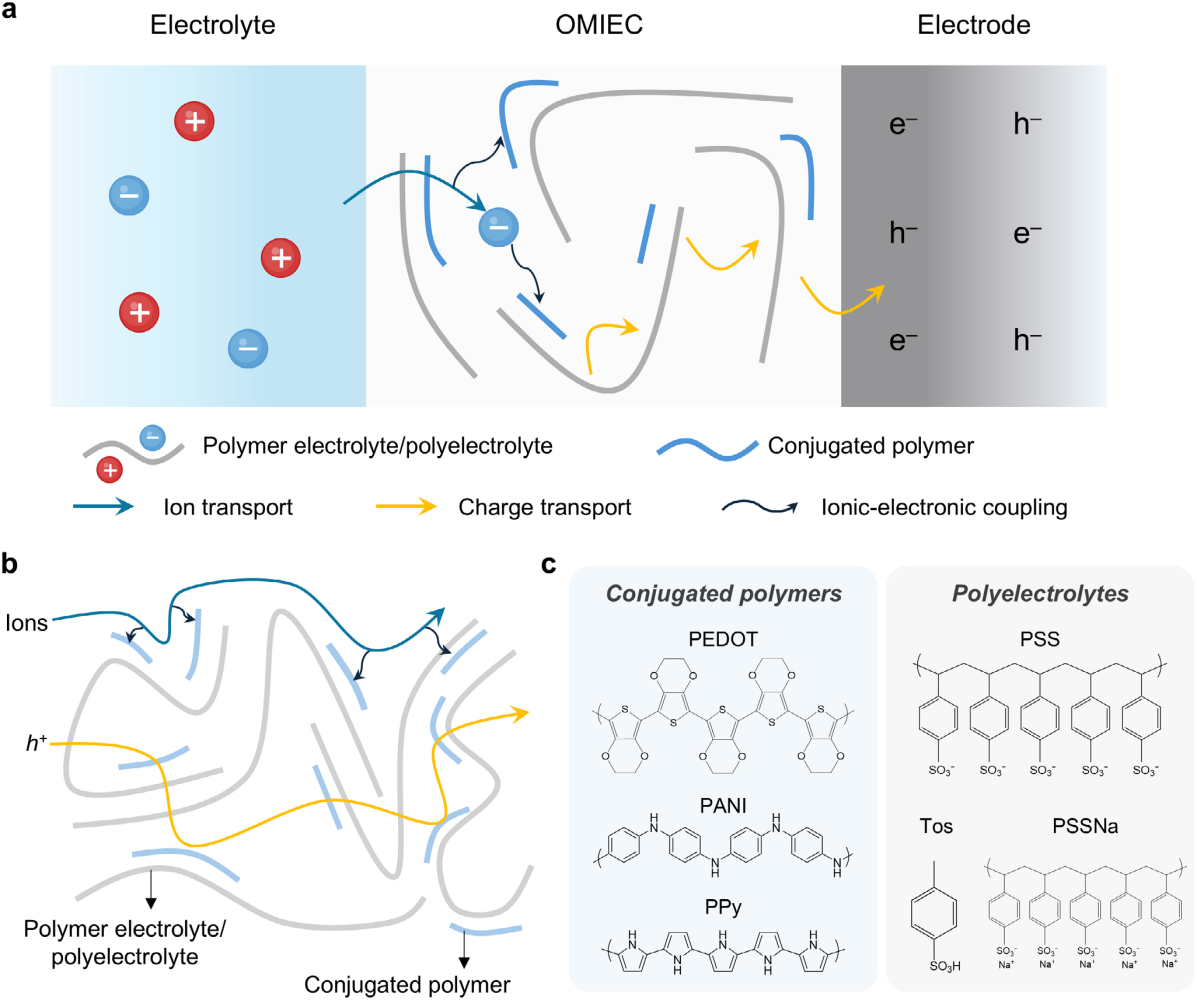
OMIECs for bioelectronic applications. a) Electrolyte–OMIEC–electrode interface dynamics. b) Electronic and ionic transport mechanisms of OMIECs. c) Chemical structures of OMIEC materials.

Organic mixed ionic‐electronic conductors (OMIECs) are promising materials for next‐generation bioelectronics, offering an effective interface between electronic devices and biological ionic systems^[^ ^190^ ^]^ (**Figure** **5**a). OMIECs simultaneously store and transport both ionic and electronic charges, enabling seamless transduction between ionic biological signals and electronic outputs. Charge transport in OMIECs involves electronic carriers stabilized by ionic dopants from the electrolyte. These carriers migrate through the material via hopping mechanisms and couple electrostatically with electrode charges. OMIECs can conduct both ionic and electronic charges throughout their bulk state.^[^ ^191^ ^]^ Owing to their relatively high free volume, OMIECs allow exceptionally large volumetric capacitance. Unlike conventional interfacial capacitors, this volumetric charging occurs throughout the active layer, enabling high charge storage and density. As a result, OMIECs establish low‐impedance and highly capacitive contacts that facilitate efficient transduction of ionic biological signals into electronic outputs for bioelectronic interfaces.

OMIEC typically consists of an electronically conductive π‐conjugated polymer and an ionically conductive components such as polyelectrolyte (Figure 5b). Electronic conduction occurs through delocalized π‐orbitals along the conjugated polymer backbone and π–π stacking between adjacent chains. Polyelectrolyte, as a fixed anionic counterion and ion‐conductive matrix, enables ion transport via hopping through its network.^[^ ^192^ ^]^ In hydrated OMIEC, proton conduction is further enhanced through the Grotthuss mechanism, in which protons hop between hydronium and water molecules via a hydrogen‐bonded network.^[^ ^190^ ^]^ Moreover, OMIEC exhibits intrinsic doping via ionic–electronic coupling between polyelectrolyte and conjugated polymer, reducing charge hopping activation energy and enhancing carrier mobility and electrical conductivity.^[^ ^190^ ^]^ Among OMIECs, poly(3,4‐ethylenedioxythiophene):poly(styrenesulfonate) (PEDOT:PSS) is one of the most widely used. ^[^ ^192^ ^]^ The synergy between ionic and electronic conduction, combined with mechanical softness and biocompatibility, makes PEDOT:PSS highly suitable for a wide range of bioelectronic applications such as electrophysiological sensing,^[^ ^193^ ^]^ neural recording,^[^ ^194^, ^195^ ^]^ and neural stimulation.^[^ ^196^, ^197^ ^]^

In addition to PEDOT:PSS, other conjugated polymer and polyelectrolyte composites have been developed to tune the ionic–electronic coupling behavior (Figure 5c). Polyaniline (PANI)^[^ ^198^ ^]^ and polypyrrole (PPy)^[^ ^199^ ^]^ are representative π‐conjugated polymers that exhibit high charge carrier mobility and large volumetric capacitance, indicating their ability to efficiently transport and store ions throughout the bulk. However, their poor solubility restricts practical processability.^[^ ^200^ ^]^ To enhance solubility and mechanical flexibility of conjugated polymers, thiophene‐based materials such as poly(3‐hexylthiophene) (P3HT) have been widely studied, where alkyl side chains improve solubility in organic solvents.^[^ ^201^ ^]^ Expanding on P3HT derivatives, the substitution of alkyl side chains with ethylene glycol (EG) groups produces glycolated polythiophenes with enhanced polarity and miscibility that facilitates ion insertion and increases volumetric capacitance.^[^ ^202^, ^203^ ^]^

For polyelectrolytes, tosylate (Tos)^[^ ^204^ ^]^ and dextran sulfate (DS)^[^ ^205^ ^]^ have been employed as alternative counterions to PSS in PEDOT‐based composites. Yet, systematic studies on the performance of these PEDOT‐based materials remain limited, and most reported composites still exhibit lower electrical properties compared to PEDOT:PSS.^[^ ^206^ ^]^

Despite its favorable electrical performance, PEDOT:PSS still suffers from the intrinsic hygroscopicity of PSS, which can compromise the environmental and operational stability of bioelectronic devices. To mitigate this issue, hydrophobic modification strategies, such as copolymerization with sodium 4‐styrenesulfonate (SSNa), have been employed to reduce moisture absorption and enhance the hydrophobicity of the resulting polymer electrolytes.^[^ ^190^ ^]^ Continued research of novel OMIEC materials that combine high conductivity, and biocompatibility will be key to advancing next‐generation bioelectronic interfaces.

#### 2.7.2 Ionic Matrices

Conventional rigid electronic materials suffer from mechanical and structural mismatch with soft biological tissues, compromising interfacial stability and long‐term biocompatibility. In response, extensive research has focused on the development of polymeric soft matrices that can better accommodate the dynamic and compliant nature of biological systems for bioelectronic applications. Ionic matrices are soft polymer network that incorporate ion species, enabling signal transduction through ion transport mechanisms. In addition to ionic conductivity, they offer mechanical compliance, self‐healing capabilities, and conformal contact with complex tissue surfaces, enabling stable and high‐fidelity interfaces. Ionic matrices are typically categorized into hydrogels, organogels, ion gels, and eutectogels, depending on their composition and ionic medium (**Table** **2**). Each class exhibits distinct advantages, as highlighted in the flowing sections.

**Table 2:** Ionic matrices.

Matrix	Ionic medium	Polymer matrix^a)^	Ionic conductivity	Advantages	Challenges
Hydrogel	Water, salts	Alginate, GelMa, HEMA, PAM, PVA	High	Softness, biocompatibility	Dehydration, low environmental stability
Organogel	Organic solvent, salts	PEGDA, PMMA	Low	Chemical stability	Low conductivity, limited biocompatibility
Ion gel	IL	PVDF‐HFP, PEO	Moderate	Moisture stability, thermal stability	High cost, toxicity, low biodegradability
Eutectogel	DES	Chitosan, agarose, HEC	Moderate	Biocompatibility, moisture stability	Variable polymer compatibility

^a)^ HEMA: 2‐hydroxyethyl methacrylate, HEC: hydroxyethyl cellulose

### 2.8 Hydrogels

Hydrogels are composed of 3D hydrophilic polymer networks swollen with water, mimicking the physicochemical properties of living tissues.^[^ ^188^, ^207^ ^]^ Ionically conductive hydrogels are typically formed by integrating aqueous electrolytes into the network, enabling the transport of solvated ions through the hydrated network. Their high‐water content and porous structure facilitate the dissociation and transport of mobile ionic species, contributing to high ionic conductivity essential for effective bioelectronic signal transduction.^[^ ^208^ ^]^ Mechanically, the low Young's modulus of hydrogels facilitates conformal contact with soft, curved, and deformable biological surfaces, reducing interfacial mechanical impedance.^[^ ^209^ ^]^ Polysaccharide‐based hydrogels derived from natural sources such as hyaluronic acid,^[^ ^210^ ^]^ alginate,^[^ ^22^ ^]^ chitosan,^[^ ^41^ ^]^ and carrageenan^[^ ^211^ ^]^ are representative hydrogels used in bioelectronics due to their excellent biocompatibility, biodegradability, and low cost.^[^ ^212^ ^]^ Polyacrylamide (PAM or PAAm) is a synthetic hydrogel with tunable stiffness, which can be adjusted by varying the concentrations of monomer and crosslinker.^[^ ^213^, ^214^ ^]^ Additionally, gelatin methacryloyl (GelMA), synthesized from gelatin, replicates essential structural and biochemical features of the native extracellular matrix (ECM), providing a bioactive, tunable, and processable platform for tissue–electronic interfaces, including printable formats.^[^ ^215^ ^]^

Hydrogels also serve as biocompatible matrices for incorporating PEDOT:PSS,^[^ ^216^ ^]^ yielding conductive, flexible, and stretchable composites suitable for electrophysiological signal recording.^[^ ^217^, ^218^ ^]^ These hydrogel–OMIEC hybrids support stable neural recording and stimulation via conformal interfacing with tissues and nerves.^[^ ^196^, ^197^, ^219^ ^]^ However, hydrogels inherently suffer from dehydration over time, leading to degradation of both mechanical integrity and electronic performance, restricting their use in long‐term bioelectronic interfaces.^[^ ^188^ ^]^ As water gradually evaporates from the polymer matrix, hydrogel undergoes shrinkage or stiffening, impairing ion transport and interfacial stability. In addition, hydrogels exhibit poor thermal stability, making reliable operation difficult under low‐ or high‐temperature conditions.^[^ ^220^, ^221^ ^]^ Consequently, ensuring long‐term and environmental stability remains a major challenge for hydrogel‐based biointerfaces. To address this limitation, strategies such as surface encapsulation^[^ ^222^, ^223^ ^]^ and the development of alternative solvent systems like organogels have been investigated to improve the environmental stability and extend operational lifespan.

### 2.9 Organogels

Organogels retain the soft polymeric network structure of hydrogels but replace water with organic solvents such as glycerol, ethylene glycol, and polyethylene glycol dimethyl ether (PEGDME), reducing evaporation while enhancing thermal and environmental stability.^[^ ^224^ ^]^ A key advantage of organogels lies in their broad solvent compatibility, which enables precise control over vapor pressure and moisture sensitivity, making organogels less susceptible to humidity‐induced deformation compared to hydrogels.^[^ ^225^ ^]^ When organic solvents serve as the primary medium, their lower volatility compared with water suppresses evaporation, further improving long‐term stability. Moreover, the broad liquid‐phase temperature range of organic solvents enables reliable operation of organogels under extreme temperature conditions.^[^ ^226^ ^]^ Furthermore, their inherently low modulus ensures mechanical compatibility with soft biological tissues.^[^ ^227^ ^]^ However, organogels often face biocompatibility and environmental sustainability challenges because of the chemical nature of many organic solvents.^[^ ^228^ ^]^ Accordingly, recent efforts have focused on developing biocompatible and low‐toxicity organogels that retain mechanical and electrical functionality.^[^ ^229^ ^]^ Polymer matrices commonly used for organogels include poly(ethylene glycol) diacrylate (PEGDA) and poly(methyl methacrylate) (PMMA), which are compatible with a wide range of organic solvents.^[^ ^230^, ^231^ ^]^ Ultimately, a comprehensive understanding of the molecular interactions between organic solvents and polymer networks is essential to achieve efficient gelation and stable structural integration.

### 2.10 Ion Gels

Unlike hydrogels that are primarily swollen with water, ion gels are semi‐solid materials, in which ILs serve as the internal medium.^[^ ^232^ ^]^ In this structure, the polymer matrix forms a 3D network that imparts mechanical strength, while the incorporated IL provides ionic conductivity. By independently tuning the cation and anion species of ILs, ion gels with a wide spectrum of tailored properties can be achieved. Poly(vinylidene fluoride‐hexafluoropropylene) (PVDF‐HFP) exhibits high mechanical strength and flexibility, and its excellent compatibility with a wide range of ILs makes it an ideal polymer matrix for ion gel.^[^ ^233^ ^]^ Polyethylene glycol (PEO or PEG) is a biocompatible matrix that exhibits ionic interaction with ILs, making it highly suitable for applications in bio iontronic systems.^[^ ^234^ ^]^ However, the intrinsic high viscosity of ILs limits ion mobility, resulting in lower ionic conductivity than hydrogels.^[^ ^16^, ^235^ ^]^ Increasing IL content improves ionic conductivity but plasticizes the polymer chains, compromising the mechanical strength and dimensional stability.^[^ ^236^ ^]^ In addition, excessive IL content or mechanical stress can lead to IL leakage or structural degradation of ion gel, raising safety concerns at biointerfaces.^[^ ^237^ ^]^ Moreover, ion gels generally exhibit weak mechanical strength, with fracture stress below 1 MPa and elastic modulus under 0.1 MPa.^[^ ^238^ ^]^ This fragility primarily arises from screening effects of ILs, which reduces electrostatic interactions and hydrogen bonding among solvated polymer chains, thereby weakening the polymer network. Therefore, designing ion gel architecture with enhanced mechanical integrity is crucial to improve its durability and structural reliability. Therefore, achieving an optimal balance between mechanical robustness and ionic transport remains a central challenge in ion gel design. One of the key advantages of ion gels is their low volatility, stemming from the low saturated vapor pressure of ILs, providing strong dehydration resistance and stable ambient operation.^[^ ^239^ ^]^ However, the limited biocompatibility of commonly used ILs constrains the use of ion gels in bioelectronic fields.^[^ ^240^, ^241^ ^]^ Incorporating biocompatible ILs, such as choline‐based cations and nature‐derived anions (e.g., glycine, acetate, lactate), can improve biofunctionality and tissue compatibility of ion gels.^[^ ^232^ ^]^

Furthermore, the inherently hygroscopic nature of many ILs remains problematic for bioelectronic applications,^[^ ^242^ ^]^ as moisture uptake alters ionic concentration and mechanical modulus, leading to interfacial instability and signal drift under humid or physiological conditions. To mitigate these effects, recent efforts have focused on developing low‐hygroscopic ILs that minimize water absorption, ensuring stable operation under ambient and physiological conditions.^[^ ^232^ ^]^ In addition, the high cost of many ILs remains a significant barrier to practical implementation. Therefore, developing low‐cost and recyclable ILs, together with biodegradable polymer networks that allow IL recovery and reuse, represents a promising pathway toward sustainable ion gel systems.^[^ ^243^ ^]^

### 2.11 Eutectogels

To address the dehydration of hydrogels and the biocompatibility issues of organogels and ion gels, eutectogels—formed by incorporating deep eutectic solvents (DESs) into crosslinked polymer networks—have emerged as promising soft ionic materials for bioelectronics.^[^ ^244^ ^]^ Natural polymer networks, such as chitosan and agarose, serve as ideal matrices for biomedical applications due to their intrinsic biocompatibility and biodegradability.^[^ ^245^ ^]^ Synthetic polymers, including poly(vinyl alcohol) (PVA) and PAM, offer tunable crosslinking densities along with favorable mechanical and biocompatible characteristics, enabling their broad use in designing eutectogels.^[^ ^246^, ^247^ ^]^ DESs are composed of hydrogen bond donors and acceptors, offering low volatility and ionic conductivity similar to those of ILs.^[^ ^248^, ^249^ ^]^ When embedded within soft polymer matrices, eutectogels retain softness and stretchability, while offering enhanced environmental stability compared to conventional water‐based hydrogels owing to the use of low‐volatility DES solvents.^[^ ^250^ ^]^ Moreover, bio‐derived DESs, such as choline chloride (ChCl)–urea and ChCl–glycerol, offer low‐cost fabrication and enhanced biocompatibility, addressing key limitations of IL‐based ion gels in bioelectronic applications.^[^ ^251^, ^252^ ^]^ However, their ionic conductivity remains lower than that of typical aqueous salt solutions.^[^ ^244^ ^]^ Therefore, continuous research is needed to develop high‐conductivity eutectogels for advanced bioelectronic systems. Recently, conductive eutectogels incorporating OMIECs into DES‐based polymer networks have been developed, exhibiting enhanced charge storage and coupled transport characteristics that enable concurrent ionic and electronic conduction.^[^ ^253^, ^254^ ^]^ This dual‐conduction capability strengthens ionic–electronic coupling at the biointerface, offering new opportunities for high‐performance and durable bioelectronic devices. Despite these advantages, eutectogels are a relatively recent innovation, and systematic studies on their gel‐state ionic transport, electrochemical stability, and long‐term biocompatibility remain limited. Further research is needed to optimize their gel‐state ionic conductivity and evaluate their long‐term electrochemical and biological performance for successful integration into practical bioelectronic devices.

#### 2.11.1 Ion‐Mediated Crosslinking

**Figure 6:**
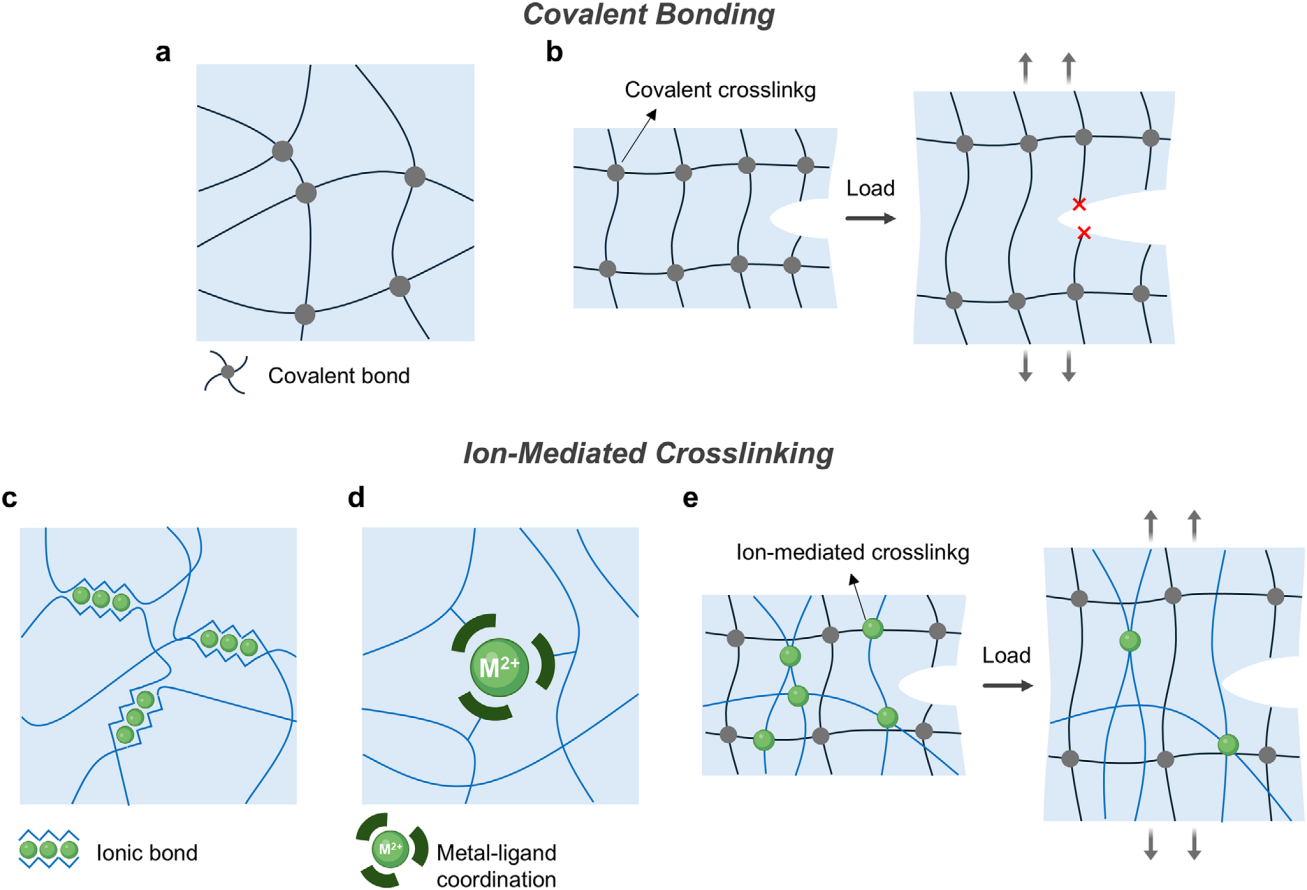
Comparison of covalent and ion‐mediated crosslinking mechanisms. a) Covalent bonding in polymer networks. b) Dissipation mechanisms of covalent‐bonded network under external mechanical load. c) Polymer network interactions mediated by ionic interactions. d) Coordination bonding through metal–ligand coordination. e) Energy dissipation mechanisms of ion‐mediated crosslinked networks under external load.

Ions play multifunctional roles in polymer networks not only as mobile charge carriers but also as structural crosslinkers, enabling dynamic and tunable interactions that enhance mechanical toughness, self‐healing, and stretchability.^[^ ^255^, ^256^, ^257^ ^]^ Conventional polymer gel‐based matrices are typically soft and elastic, but their low toughness limits their practical applicability under mechanical stress.^[^ ^258^ ^]^ This mechanical brittleness primarily arises from permanent covalent bonding, which are strong but lack reversibility and energy dissipative capacity (**Figure** **6**a). Under external load, stress concentrates at covalent bonding points that cannot deform reversibly. As a result, energy is not efficiently dissipated throughout the polymer matrix, leading to localized stress accumulation, crack initiation, and rapid fracture propagation (Figure 6b).

To address the limitations of covalent bonding, ion‐mediated crosslinking (Figure 6c) has been introduced in combination with static covalent bonding, providing reversible interactions that dissipate energy and enhance mechanical resilience.^[^ ^259^ ^]^ Ionic crosslinking refers to the formation of non‐covalent bonds between polyelectrolyte chains and oppositely charged multivalent ions. For example, anionic alginate forms physically crosslinked networks with divalent or trivalent ions such as Ca^2+[^ ^260^ ^]^ and Fe^3+^,^[^ ^261^ ^]^ The resulting ionically crosslinked polymers form physically reversible and mechanically cohesive networks, effectively enhancing the mechanical toughness of soft materials.^[^ ^262^, ^263^ ^]^ Notably, the dynamic and reversible nature of ionic crosslinks enables self‐healing and reprocessability, which are desirable for stretchable bioelectronic interfaces.^[^ ^264^ ^]^ Although ionic crosslinking typically has lower bond energies (5–200 kJ mol^−1^), densely packed charged groups within the polymer matrix can form locally crosslinked domains that contribute to the structural stability.^[^ ^265^ ^]^

Another well‐established ion‐mediated approach involves metal–ligand coordination (Figure 6d), where transition metal ions (e.g., Fe^3+^, Zn^2+^, Cu^2+^, Ni^2+^, Ca^2+^) form strong and reversible bonds with functional groups such as catechols, biphosphonates, or carboxylates.^[^ ^77^, ^266^ ^]^ These metal–ligand complexes act as dynamic crosslinking that provides mechanical reinforcement, structural adaptability, self‐healing, and rapid gelation.^[^ ^261^, ^267^, ^268^ ^]^ The number and strength of coordination bonds can be tuned by the valence state of the metal ion, offering opportunities to develop redox‐responsive bioelectronic interfaces.^[^ ^35^ ^]^ A particularly robust coordination bonding is metal chelation, where multidentate ligands form stable complexes with metal ions.^[^ ^269^ ^]^ Chelation enhances crosslinking stability through the chelate effect, thereby exhibiting superior toughness. Coordination interactions provide higher bond energies (100–300 kJ mol^−1^) than ionic crosslinking, contributing to enhanced toughness.^[^ ^265^ ^]^ However, their application in bioelectronic system requires careful selection of metal ions that are biocompatible.

Covalent and ion‐mediated crosslinking mechanisms exhibit distinct characteristics (**Table** **3**). Incorporating ions into soft polymer enables dynamic crosslinking architectures that complement conventional covalent or physical networks. These ion‐mediated crosslinking introduces reversible and dynamic interactions into polymer networks, where ionic interactions function as sacrificial bonds that can reversibly dissociate and reform under mechanical stress (Figure 6e). This dynamic behavior allows for efficient energy dissipation and supports various functionalities, such as self‐healing, reprocessability, and rapid gelation. Together, these properties offer a versatile platform for developing next‐generation bioelectronic interfaces that require both mechanical compliance and functional adaptability.

**Table 3:** Ionic and non‐ionic crosslinking.

Ionic/non‐ionic	Crosslinking	Type	Mechanism	Ion	Polymer backbone	Bonding energy^[^ ^265^ ^]^	Property
Non‐ionic	Covalent bonding	Chemical crosslinking	Chemical interaction	‐	Functional group‐containing synthetic polymers (PVA, PEG, PAA, PAM)	High (200–500 kJ mol^−1^)	Permanent, chemically stable
Ionic	Ionic crosslinking	Dynamic crosslinking	Electrostatic interaction	Divalent ion (Ca^2+^, Ba^2+^, Mg^2+^)	Charged polymers (alginate, chitosan)	Low (5–200 kJ mol^−1^)	Reversible, self‐heating, fast gelation, stimuli‐responsive
	Coordination interaction		Metal‐ligand coordination	Transition metal ion (Fe^3+^, Zn^2+^, Cu^2+^, Ni^2+^) Metalloid ion (BO_3_^3−^)	Ligand functionalized polymers (catechol‐, carboxylate‐, histidine‐based polymer)	Moderate (100–300 kJ mol^−1^)	

### 2.12 Bioelectronic Applications through Ionic–Bionic Interfaces

**Figure 7:**
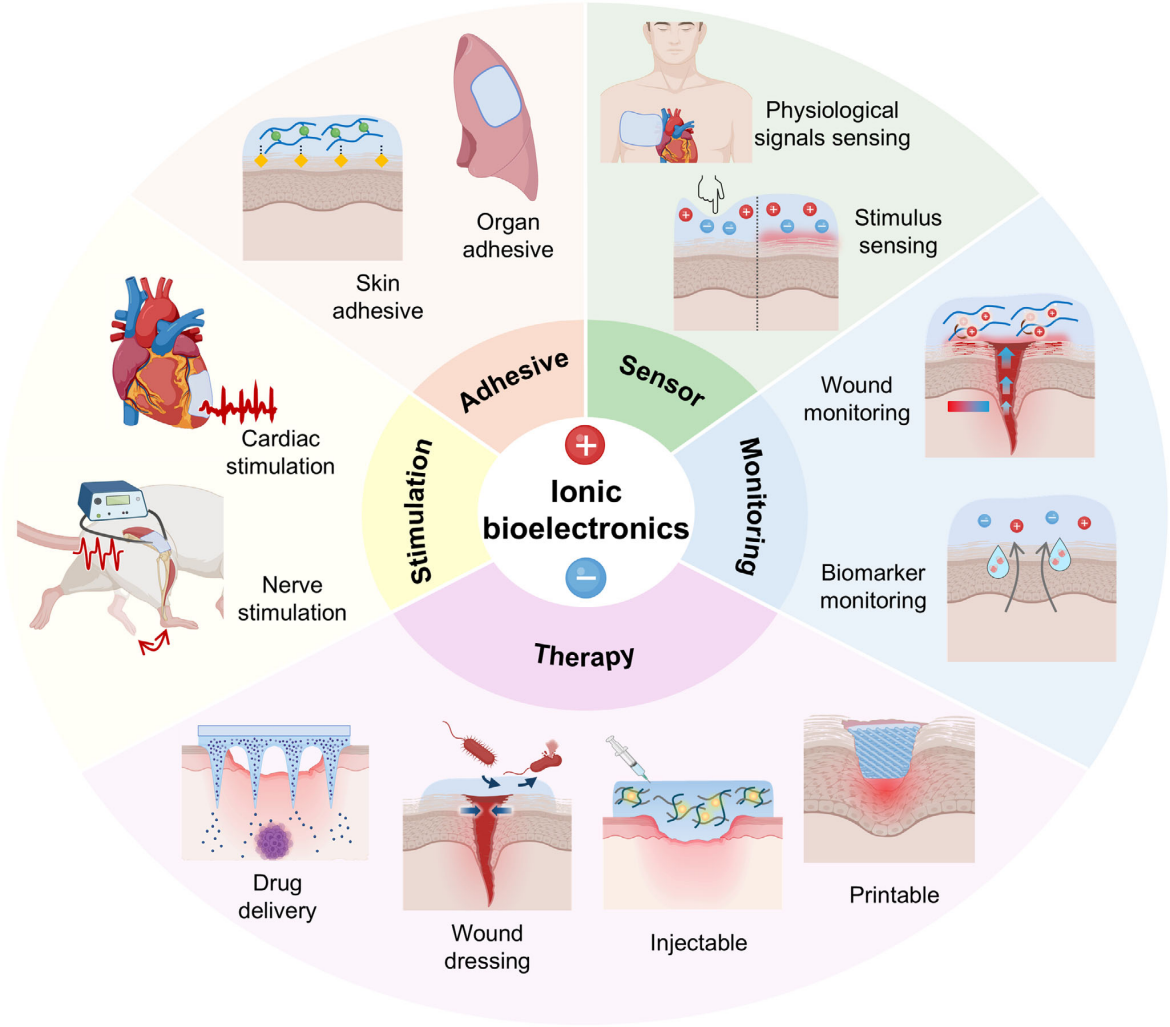
Multifunctional applications of ionic bioelectronics. Adhesive: wound dressing and bioelectrodes. Sensor: detection of electrophysiological signals and physiological parameters such as pressure and temperature. Monitoring: tracking wound status and biomarker levels. Therapy: drug delivery, wound dressing, and application via injectable or printable therapeutic platforms. Stimulation: cardiac and nerve stimulation. Schematics were created using BioRender.com.

Driven by the versatile functionality of ionic species, ionic materials and iontronic devices are rapidly advancing as essential components in bioelectronic systems, enabling dynamic, biocompatible, and seamlessly integrated ionic–bionic interfaces (**Figure** **7**). In adhesive applications, ion‐conductive materials offer conformal contact with biological tissues, functioning as epidermal electrodes for signal acquisition while simultaneously promoting wound closure. In sensing, iontronic devices enable real‐time monitoring of electrophysiological signals, local wound states, and biomarkers, providing localized and dynamic insights into physiological conditions. In the stimulation domain, iontronic systems deliver targeted electroceutical therapies, such as cardiac and nerve stimulation, to actively modulate physiological functions. For therapeutic applications, ionic materials support controlled drug delivery and bioactive wound dressings and are implemented in injectable systems and printable bioelectronic platforms for personalized and on‐demand treatment strategies. Altogether, the integration of soft, ion‐conductive materials with electronic interfaces establishes a multifunctional framework for next‐generation bioelectronic systems.

## 3 Adhesive, Wearable, and Epidermal Interfaces

Seamless integration between biological tissues and electronic systems requires materials that can interface both mechanically and electrically with the soft, hydrated, ion‐rich environments of biological tissues. However, stable adhesion and efficient signal transduction remain challenging due to the dynamic and hydrated nature of tissues.^[^ ^270^ ^]^ Traditional rigid electronics often fail to conform to such environments, leading to mechanical mismatch, poor interfacial contact, and signal degradation. To address these limitations, ions play multifunctional roles, not only as mobile charge carriers but also as structural and chemical mediators that enhance interfacial adhesion, electrical conductivity, and overall stability.

**Figure 8:**
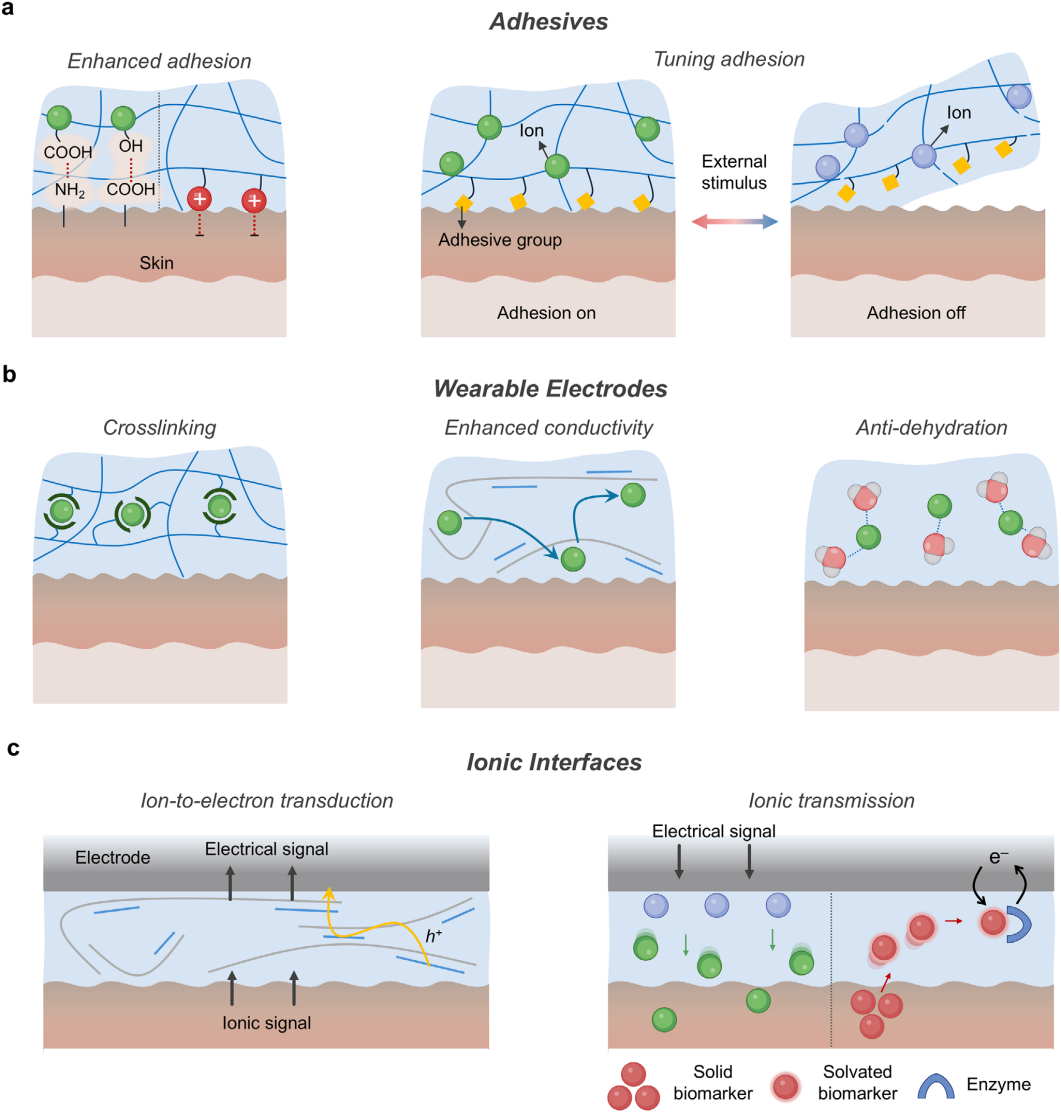
Ionic biointerface systems. a) Schematic illustration of ion‐based bioadhesives, enabling enhanced and tunable adhesion. b) Schematic illustration of ion‐based wearable electrodes, featuring ion‐mediated crosslinking, improved conductivity, and anti‐dehydration. c) Schematic illustration of ionic interfaces facilitating ion‐to‐electron transduction and ionic signal transmission.

Mechanically, ions strengthen tissue adhesion through hydrogen bonding^[^ ^33^, ^271^ ^]^ and electrostatic interactions,^[^ ^272^, ^273^ ^]^ forming strong initial contacts with biological surfaces (**Figure** **8**a). In addition, stimuli‐responsive ion‐based adhesives can offer tunable adhesion in response to triggers such as light,^[^ ^35^ ^]^ heat,^[^ ^274^ ^]^ or moisture,^[^ ^34^ ^]^ supporting dynamic interfacing with fragile tissues. Electrically, ion‐containing polymer networks in iontronic electrodes emulate biological ionic conduction (Figure 8b). Here, ions act as charge carriers and modulate mechanical and chemical properties through ion‐mediated crosslinking. For example, the incorporation of ionic dopants into PEDOT:PSS promotes dual ionic–electronic conduction,^[^ ^275^ ^]^ while embedding metal ions in hydrogels enhances water retention and prevent dehydration in hydrogels.^[^ ^119^ ^]^ At the ionic‐electronic interface, ions help bridge the ionic–electronic charge carrier mismatch, enabling efficient signal conversion,^[^ ^276^ ^]^ and bidirectional communication between biological and electronic systems^[^ ^231^ ^]^ (Figure 8c). These ionic pathways also support electrochemical sensing of soluble biomarkers such as lactate and cholesterol.^[^ ^277^ ^]^ Overall, ions serve as critical mediators of both mechanical integration and electrical communication at the biointerface, driving the performance and versatility of bioadhesives, wearable electrodes, and ionic transduction platforms in advanced bioelectronic applications.

### 3.1 Adhesives

Achieving strong adhesion to biological tissues is crucial for a wide range of biomedical and bioelectronic applications, including tissue engineering,^[^ ^278^ ^]^ biosensing,^[^ ^279^ ^]^ and implantable systems.^[^ ^280^ ^]^ However, maintaining robust adhesion to human skin or internal organs remains a significant challenge due to the microscale roughness,^[^ ^281^ ^]^ dynamic mechanical motions,^[^ ^282^ ^]^ and persistent moisture from sweat or bodily fluids.^[^ ^270^ ^]^ To address these issues, extensive research has focused on developing bioadhesives that conform and adhere effectively to complex biological surfaces. Soft polymeric materials such as hydrogels and organogels have attracted significant interest due to their tissue‐like compliance and biocompatibility.^[^ ^208^, ^224^, ^283^ ^]^ Recently, gel‐type bioadhesives have recently demonstrated not only robust interfacial adhesion,^[^ ^284^, ^285^, ^286^ ^]^ but also tunable adhesion strength^[^ ^287^, ^288^, ^289^ ^]^ on soft and fragile biological surfaces. Notably, incorporating ions into the polymer matrix has emerged as a powerful strategy to enhance adhesive performance. Ions facilitate dynamic crosslinking,^[^ ^143^ ^]^ strengthen interfacial interactions,^[^ ^33^, ^290^, ^291^ ^]^ and enable stimulus‐responsive behavior.^[^ ^35^ ^]^ These ion‐mediated adhesive systems allow for stable adhesion under physiological conditions and offer tunability of adhesion strength for specific biomedical applications (**Table** **4**).

**Table 4:** Adhesives.

Types	Ionic material^a)^	Polymer matrix^b)^	Functions of ions	Adhesive force (target)	Applications	References
Enhanced adhesion	Ca^2+^	Silk fibroin	Electrostatic interactions	180 J m^−2^ (dog tooth)	Hard tissue monitoring and repair	[273]
			Increased viscoelastic property	≈ 400 N m^−2^ (skin)^c)^	Epidermal electronics and drug delivery system	[266]
	CAC	PDMAA	Hydrogen bonding	38.3 kPa (porcine skin)	Wound closure, lift porcine heart, epidermal electronics	[33]
	Choline acrylate	GelMA	Electrostatic interactions	359.39 kPa (skin)	Puncture, sealing, and patching of the wound	[272]
	Fe^3+^	Dopamine‐modified gelatin	Crosslinking with tissue proteins	≈ 5 N cm^−2^ (porcine skin)^c)^	Repair of visceral injury	[290]
	[N_4111_][TFSI]	P(BA‐co‐MAA)	Hierarchical crosslinking	370 J m^−2^ (porcine skin)	Epidermal electronics	[143]
	MPC	Dopamine acrylamide	Hydrogen bonding and dipole‐dipole interaction	34.7 kPa (pig skin)	Epidermal electronics	[271]
Tunable adhesion	Fe^3+^, Fe^2+^	PAA	Reversible coordination bonding	13.10 kPa–74.79 kPa (skin)	Epidermal electronics	[35]
	[HPVIm][TFSI], boric acid	MEA	Tunning adhesion under aqueous environment	15 N m^−1^–180 N m^−1^ (glass)	Epidermal electronics under aqueous environment	[34]
	Zn^2+^	Dopamine‐modified hyperbranched poly(amino ester)	Ion‐responsive adhesion	12 kPa–37.1 kPa (glass)^c)^	Wound healing	[287]
Enhanced mechanical property	Al^3+^	PAA	Coordination bonding for self‐healing and high stretchability	7.02 kPa (hogskin)	Epidermal electronics	[256]

^a)^ CAC: citric acid/L‐(−)‐carnitine‐based IL, MPC: 2‐methacryloyloxyethyl phosphorylcholine, [HPVIm]: 1‐hydroxypropyl‐3‐vinylimidazolium

^b)^ PDMAA: poly(N,N‐dimethylacrylamide), P(BA‐co‐MAA): poly(butyl acrylate‐co‐methacrylic acid), PAA: polyacrylic acid, MEA: 2‐methoxyethyl acrylate, NIPAM: N‐isopropyl acrylamide, OMA: octadecyl methacrylate

^c)^ Performance demonstrated in the figure.

**Figure 9:**
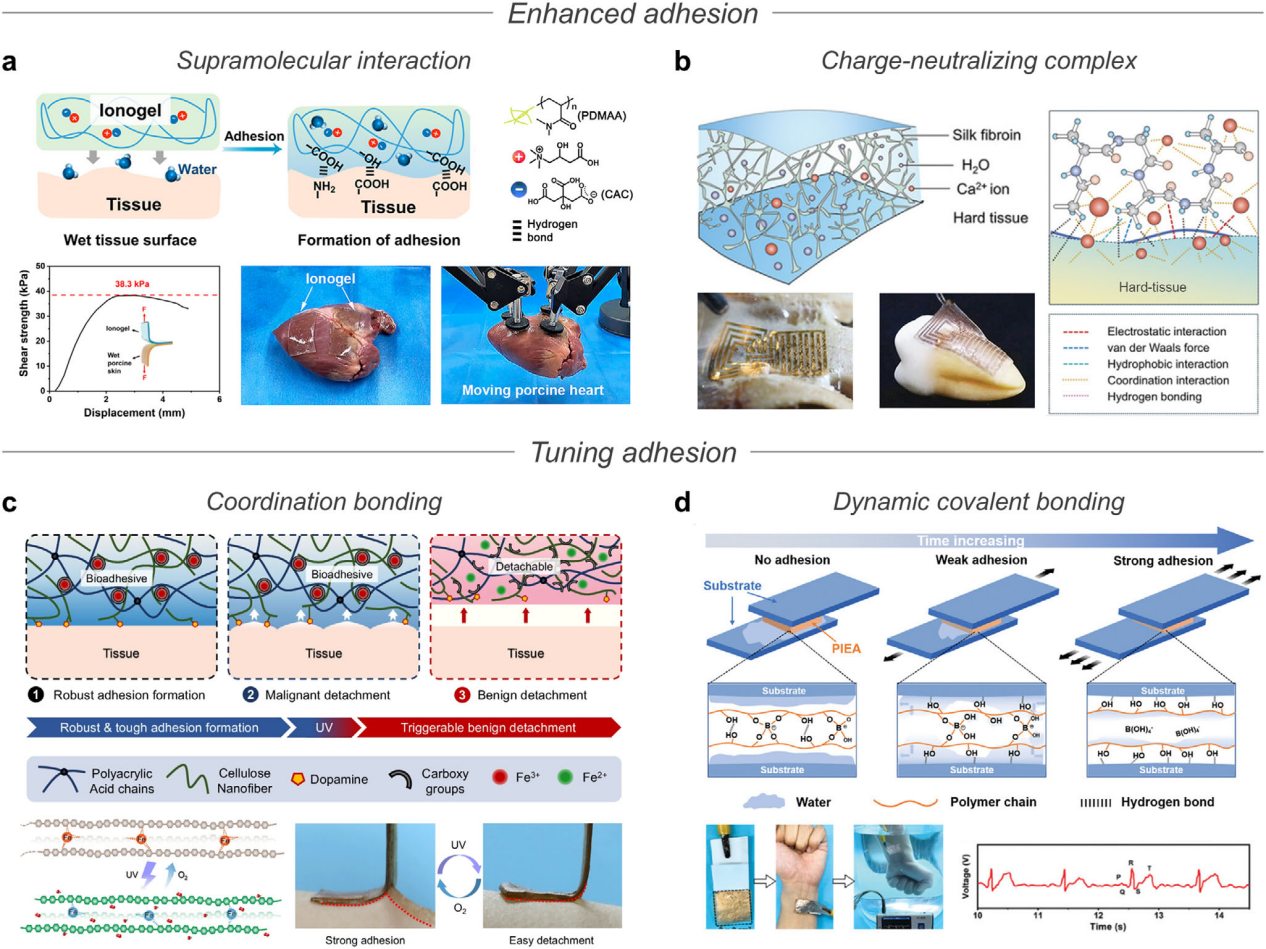
Bioadhesive system. a) Supramolecular interaction mechanism of ionogels showing hydrogen bonding interactions that enable tough adhesion and mechanical compatibility. Demonstration of adhesion by lifting a porcine heart using two ionogel adhesives. Reproduced with permission.^[^ ^33^ ^]^ Copyright 2024, American Chemical Society. b) Mechanism of hard tissue adhesion using an ionogel‐based bioadhesive and demonstration of adhesion to calvarial bone and human teeth. Reproduced with permission.^[^ ^273^ ^]^ Copyright 2024, Wiley‐VCH. c) Reversible adhesive structure: photo‐detachable dynamic hydrogel formed via a light‐driven supramolecular network based on the photo‐Fenton‐like reaction and removed from human skin under UV irradiation. Reproduced with permission.^[^ ^35^ ^]^ Copyright 2024, Springer Nature under CC BY 4.0 license http://creativecommons.org/licenses/by/4.0/. d) Mechanism of the poly(ionic liquid) elastomer adhesive enabling ECG monitoring in aqueous environments. Reproduced with permission.^[^ ^34^ ^]^ Copyright 2024, Wiley‐VCH.

To achieve strong adhesion to biological tissues, ions have been introduced into adhesive systems to facilitate stable interfacial bonding through specific interactions with functional groups on the tissue surfaces. For instance, Xiong et al. developed a bioadhesive ionogel incorporating a biocompatible IL composed of citric acid and L‐(−)‐carnitine (CAC), along with a matrix of *N,N*‐dimethylacrylamide (DMAA) (**Figure** **9**a).^[^ ^33^ ^]^ This system forms a supramolecular network through abundant hydrogen bonding and electrostatic interactions, resulting in a hydrophilic ionogel adhesive that rapidly absorbs interfacial water on biological tissues, forming a tissue‐compatible modulus. Moreover, the ionogel is rich in carboxyl (–COOH) and hydroxyl (–OH) groups originating from CAC, which physically crosslink with reactive functional groups on the tissue surfaces, such as amino (–NH_2_) and carboxyl (–COOH) groups, thereby enhancing adhesion strength. The ionogel adhesive demonstrated a high shear strength of 38.3 kPa and wound closure strength of 3.5 N on wet porcine skin. In ex vivo experiments, the ionogel was applied to a porcine lung incision to achieve sealing, and two ionogel patches adhered to a porcine heart were able to gradually lift the organ using a mechanical manipulator during simulated heart transplantation. These findings highlight that the biocompatible IL facilitate the formation of a dense supramolecular network via extensive hydrogen bonding, imparting the ionogel with mechanical robustness and reliable tissue adhesion, which are critical for biomedical applications.

Beyond soft tissues, there is growing demand for integrating bioelectronic devices with hard tissues, such as bones and teeth, for diagnostic and therapeutic applications. However, their high stiffness and smooth surfaces limit adhesion by impeding adhesive infiltration and mechanical interlocking. To address this challenge, Jiang et al. developed an ionogel‐based hard tissue–adhesive interface by incorporating calcium ions (Ca^2+^) into silk fibroin to enhance ionic interactions with hard tissues (Figure 9b).^[^ ^273^ ^]^ The silk fibroin, a biocompatible amphiphilic protein, provides abundant hydrogen bonding sites, while the incorporated Ca^2+^ ions formed stable, charge‐neutralizing complexes with negatively charged biomolecules present in hard tissues, thereby enhancing interfacial adhesion. This dual adhesion mechanism combining extensive hydrogen bonding and strong ionic interactions enabled robust bonding to hard tissue substrates, achieving shear strengths exceeding 220 kPa on calvarial bone and 240 kPa on tooth surfaces. Owing to its strong adhesion to both soft and hard tissue surfaces, the ionogel was employed for bone defect repair by adhering to the defect site and serving as a biological barrier to prevent soft tissue invasion and ensure proper bone regeneration. It was also applied in canine tooth reimplantation, where the extracted tooth root was wrapped with the ionogel, mimicking the function of the periodontal ligament and promoting integration between the root and alveolar bone. Notably, the self‐reinforcing nature of the silk fibroin network, specially designed for hard tissue applications, effectively addresses the interfacial and mechanical challenges associated with bone and dental adhesion.

Although strong adhesion to human tissue ensures reliable attachment, excessive adhesive force can cause damage upon removal. Given the fragility of human skin and internal organs, it is crucial to design adhesives with tunable adhesion properties. By allowing the adhesive strength to be selectively reduced after application, the risk of tissue injury during detachment can be substantially minimized. One effective strategy involves harnessing ions to induce dynamic transformations within the supramolecular network. Zhang et al. developed a composite hydrogel adhesive with reversible and tough adhesion by incorporating photo‐responsive Fe^3+^ ions into the adhesive system (Figure 9c).^[^ ^35^ ^]^ The bioadhesive consists of cellulose nanofibers (CNFs) and poly(acrylic acid) (PAA) chains functionalized with dopamine (DA) groups, which are further coordinated with Fe^3+^. The Fe^3+^ form reversible coordination bonds with carboxyl groups in the hydrogel network, enabling dynamic and controllable adhesion. The combination of oxygen‐containing functional groups on CNFs and the dopamine‐based adhesion mechanism imparts strong bonding to skin surfaces. Upon UV irradiation, Fe^3+^ are reduced to Fe^2+^ via a photo‐Fenton‐like reaction, leading to the dissociation of interactions between Fe ions and the CNF network. As a result, the adhesion strength on skin significantly decreases, with the tensile strength decreasing from 74.79 kPa to 13.10 kPa, enabling photo‐triggered detachment. Notably, this process utilizes low‐intensity, human‐friendly UV light (≤ 40 mW cm^−2^ for 5 min), which enables on‐demand detachment from the skin without any observable irritation or tissue damage. This work underscores the potential of ion‐mediated coordination networks for developing reusable and photo‐responsive bioadhesives.

While prior studies have largely concentrated on achieving adhesion under ambient conditions, establishing durable adhesion in aqueous environments is essential for applications such as tissue engineering and underwater communication. However, achieving reliable adhesion in such environments remains inherently challenging due to the presence of an interfacial water layer that disrupts effective interaction between the adhesive and the target surface.^[^ ^292^ ^]^ This challenge can be addressed by employing poly(ionic liquid), which allow for the modulation of hydrophilicity and hydrophobicity while promoting strong ionic interactions capable of displacing interfacial water.^[^ ^34^, ^293^ ^]^ Li et al. developed an underwater adhesive by copolymerizing a hydrophobic IL monomer, 1‐hydroxypropyl‐3‐vinylimidazolium bis(trifluoromethanesulfonyl)imide ([HPVIm][TFSI]), with 2‐methoxyethyl acrylate (MEA) and boric acid (BA) (Figure 9d).^[^ ^34^ ^]^ The resulting poly(ionic liquid) contains hydrophilic hydroxyl groups on its side chains, which readily form dynamic covalent borate ester bonds with BA. While the adhesive exhibits relatively weak bonding in air, immersion in water induce gradual dissociation of the borate ester bonds, thereby exposing hydroxyl groups. These groups migrate to the adhesive surface and facilitate the formation of strong hydrogen bonds at the interface, resulting in spontaneous and enhanced underwater adhesion. Meanwhile, the weak hydrogen bonding capability of the C–F bonds in the hydrophobic [TFSI]^−^ anion minimizes interference from surrounding water molecules. Furthermore, the hydrophobic segments of the polymer network ([HPVIm][TFSI] and MEA) assist in displacing excess interfacial water, promoting intimate contact and strong bonding with the target substrate. Leveraging both the water‐enhanced adhesion and intrinsic ionic conductivity, the adhesive enabled stable ECG monitoring under underwater conditions when applied to human skin. Its spontaneous adhesion and excellent underwater performance suggest broad applicability in marine exploration, wet‐surface attachment, and real‐time underwater bioelectronic monitoring.

Recent studies have demonstrated that incorporating ILs,^[^ ^33^ ^]^ zwitterionic groups,^[^ ^271^ ^]^ and metal ions^[^ ^273^ ^]^ into polymeric networks enables controlled modulation of interfacial adhesion. These material‐level improvements are especially important for wearable and implantable devices, which require stable and biocompatible contact with dynamic and moisture‐rich biological surfaces. To be effective across diverse biological sites such as skin, internal organs, and hard tissues, ion‐based adhesives should be designed to accommodate varying biomechanical environments and hydration levels, while maintaining strong adhesion. In particular, for implantable applications, additional considerations such as biodegradability, tissue compatibility, and minimal immune response are essential to ensure long‐term in vivo stability and functionality.

### 3.2 Wearable and Epidermal Electrodes

Over the past decade, wearable and epidermal electrodes have emerged as essential skin‐interfacing platforms for physiological signal monitoring,^[^ ^294^ ^]^ neuromodulation,^[^ ^295^ ^]^ and HMIs.^[^ ^296^ ^]^ Conventional rigid metallic electrodes, such as those made of gold (Au), silver (Ag), silver/silver chloride (Ag/AgCl), and stainless‐steel, offer excellent electrical conductivity but suffer from poor mechanical compatibility with soft, deformable skin. This mismatch can cause discomfort, motion‐induced artifacts, and poor long‐term adhesion.^[^ ^297^, ^298^ ^]^ To migrate this issue, recent studies have explored intrinsically soft ion‐conductive polymeric networks that either replace rigid metallic electrodes or function as interfacial ionic layers, effectively bridging metallic electrodes and biological tissues to reduce impedance and improve conformal contact.^[^ ^299^, ^300^ ^]^ These iontronic electrodes also exhibit high mechanical compliance,^[^ ^301^, ^302^, ^303^ ^]^ self‐healing capabilities,^[^ ^304^ ^]^ and enhanced interfacial adhesion with skin.^[^ ^272^, ^290^, ^305^ ^]^ As a result, iontronic electrodes can establish stable and low‐impedance interfaces with the skin, even under dynamic mechanical deformation, which are typical in wearable and epidermal applications (**Table** **5**).

**Table 5:** Wearable and epidermal electrodes.

Types	Ionic material^a)^	Polymer matrix^b)^	Functions of ions	Conductivity (S/m)	Applications	References
Enhanced mechanical property	[N_4111_][TFSI]	P(BA‐co‐MAA)	Hierarchical crosslinking	2.6 × 10^−4^	ECG, EMG monitoring	[143]
	Betaine	PAA	Ionic interactions	2.0 × 10^−5^ (RH 20%)	Finger bending, swallowing monitoring	[301]
	Ga^3+^, Al^3+^	P(AA‐co‐AU)	Coordination bonding	0.39	ECG, EMG monitoring	[302]
	Ca^2+^	PVA/b‐PEI		3.09	ECG, EMG, EEG monitoring	[303]
Enhanced conductivity	ChCl	PAA	Introducing ionic conductivity	0.126	EMG monitoring	[299]
	ChCl	PAM		1.6	ECG, EMG monitoring	[247]
	IP6	PVA		3.3 × 10^−2^	ECG, EMG, EEG monitoring	[300]
	[EMIM][TFSI]	PUF		2.6 × 10^−3^	ECG, sEMG monitoring	[142]
	Glycerol, ChCl	Gelatin	Enhanced conductivity of PEDOT:PSS	‐	ECG, sEMG monitoring, in vivo cardiac sensor	[275]
Anti‐dehydration capability	MPC	PAAm	Hydrogen bonding with water molecules for anti‐dehydration	‐	ECG, plant electrophysiology monitoring	[119]
Modulated phase transition behavior	Sodium citrate	Gelatin	Coordination bonding for phase transition temperature modulation	‐	EEG monitoring	[307]

^a)^ [N_4111_][TFSI]: butyltrimethylammonium bis(trifluoromethylsulfonyl)imide, IP6: inositol hexakisphosphate, PU: polyurethane

^b)^ P(AA‐co‐AU): poly(acrylated adenine‐co‐uracil), b‐PEI: branched polyethyleneimine, PUF: fluorinated polyurethane

**Figure 10:**
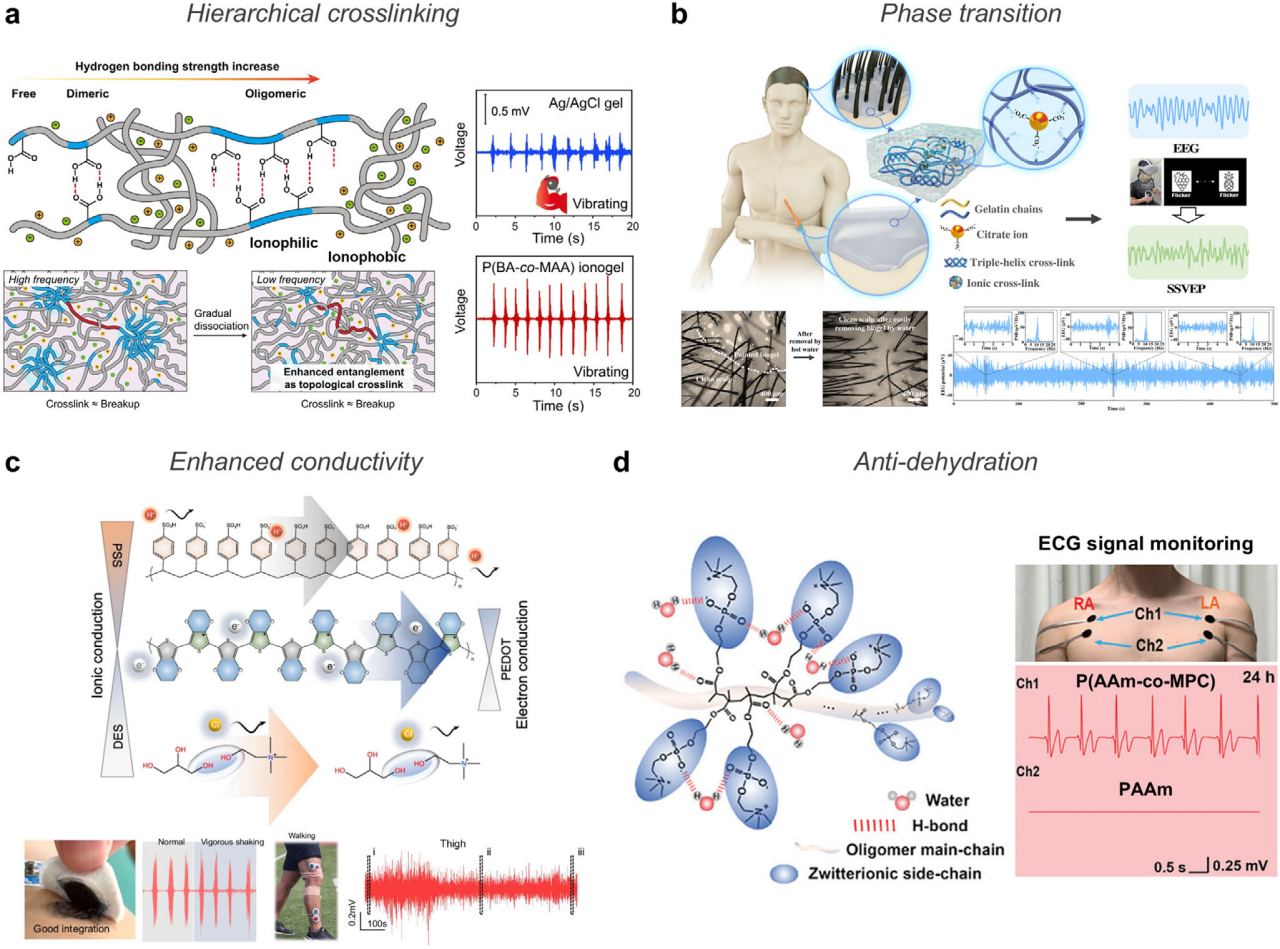
Wearable and epidermal electrode systems. a) Chemical structure of P(BA‐co‐MAA) ionogel and distribution of hydrogen bonding strengths. As the frequency decreases, physical interactions progressively dissociate, resulting in simultaneous interchain breakup and enhanced entanglement that serve as topological crosslinks. Reproduced with permission.^[^ ^143^ ^]^ Copyright 2024, Springer Nature under CC BY 4.0. license http://creativecommons.org/licenses/by/4.0/. b) On‐skin paintable biogel for EEG recording on a hairy scalp. Biogel is painted on the scalp and removed with water, leaving the skin clean. Reproduced with permission.^[^ ^307^ ^]^ Copyright 2022, The American Association for the Advancement of Science. c) In situ rapid gelation process of the biogel and formation mechanism of a conductive network for sEMG signal monitoring during vigorous motion. Reproduced with permission.^[^ ^275^ ^]^ Copyright 2025, Springer Nature. d) Molecular dynamics (MD) simulation showing that the MPC_10_ oligomer forms multiple hydrogen bonds with water molecules. ECG signals recorded using the anti‐dehydration hydrogel (P(AAm‐co‐MPC)) and conventional PAAm hydrogel after 24 h. Reproduced with permission.^[^ ^119^ ^]^ Copyright 2023, Wiley‐VCH.

Minimizing the mechanical mismatch between soft skin and rigid electronic components is essential for ensuring stable, long‐term operation of epidermal bioelectronic systems. To address this issue, researchers have developed various epidermal electrodes using soft polymer matrices or ultrathin electronic films to improve compliance with the skin.^[^ ^306^ ^]^ However, human skin continuously undergoes complex deformations such as bending, stretching, and shaking, which can lead to interfacial failure and motion‐induced signal artifacts.^[^ ^187^ ^]^ Therefore, beyond simply reducing mechanical mismatch, there is a growing need for materials that can actively conform to real‐time skin deformations. Ye et al. developed a self‐compliant ionogel electrode based on a phase‐separated hierarchical crosslinking architecture that enables a frequency‐independent gel point (**Figure** **10**a).^[^ ^143^ ^]^ Generally, viscoelastic materials exhibit frequency‐dependent transition between viscous and elastic states; at low frequencies, interchain bonds dissociate and the material behaves like a viscous sol; at high frequencies, physical interactions dominate, favoring elastic gel behavior. Achieving a stable gel point across a wide frequency range remains a challenge. Ye et al. addressed this limitation by designing a phase‐separated ionogel system composed of poly(butyl acrylate‐*co*‐methacrylic acid) (P(BA‐*co*‐MAA)) and the IL butyltrimethylammonium bis(trifluoromethylsulfonyl)imide ([N_4111_][TFSI]). The phase‐separated morphology arises from the incompatibility of PMAA chains (ionophobic) with both PBA and IL, whereas PBA (ionophilic) is well‐mixed with IL. This phase separation facilitates the formation of hierarchical hydrogen bond aggregates with a wide distribution of bonding strengths. As frequency decreases, these hydrogen bonds dissociate gradually, releasing polymer chains that form entanglements, which serve as topological crosslinks. This interplay maintains the gel point condition over a wide frequency range (10^−11^–500 Hz), enabling robust stress relaxation during repeated deformation. When applied to the arm for epidermal EMG while holding a 100 Hz vibrating ball, the iongel‐based electrode produced significantly more stable signals compared to commercial Ag/AgCl gel electrodes.

In addition to the challenge of achieving mechanical compliance with deformable skin, establishing conformal contact at biological interfaces is further complicated by the surface irregularities such as microscale roughness and hair. This issue is especially critical for electroencephalogram (EEG) recording, where dense hair coverage on the scalp can significantly interfere with stable electrode–skin contact. To overcome this limitation, Wang et al. developed an on‐skin paintable conductive biogel that undergoes a temperature‐controlled reversible phase transition between a fluidic state and a viscoelastic gel state (Figure 10b).^[^ ^307^ ^]^ This biogel consists of a gelatin matrix incorporating sodium chloride for ionic conductivity, sodium citrate for ionic crosslinking, and a water–glycerol binary solvent system for long‐term moisture retention. The gelatin undergoes a reversible sol‐gel transition due to non‐covalent triple‐helix and ionic crosslinks. When heated, the biogel becomes fluid and can be painted directly onto skin surfaces. As it cools to skin temperature, it undergoes in situ gelation, forming a mechanically robust and adhesive interface that conforms to the surface microstructure of the skin, including wrinkles and hair. To optimize the gelation temperature for on‐skin application, the crosslinking density of the gelatin is modulated by incorporating sodium citrate. The tricarboxylate structure of sodium citrate forms ionic crosslinks with amino groups in the gelatin, creating amine–carboxylate electrostatic domains that increases the crosslinking density. When painted on the scalp, the biogel demonstrated excellent EEG signal quality over prolonged wear. Furthermore, the biogel can be safely removed by applying warm water, owing to its thermally reversible network structure.

Incorporating ions into biogels not only enables the modulation of mechanical properties but also contributes to enhanced conductivity. High conductivity is an essential characteristic of bioelectric electrodes, as it ensures efficient charge transport and minimizes signal loss during signal acquisition. Li et al. developed a temperature‐responsive, in situ forming biogel with high conductivity, composed of a gelatin matrix, PEDOT:PSS as the conductive component, and a DES based on glycerol and ChCl (Figure 10c).^[^ ^275^ ^]^ The biogel remains fluidic above 60 °C and undergoes rapid gelation within 3 min at room temperature through hydrogen bonding and physical crosslinking, forming a conformal contact with the skin. The hydrogen bonding between ChCl and the carbonyl/hydroxyl groups of gelatin modulates the thermoresponsvie sol‐gel transition behavior. This interaction disrupts triple‐helix domains and enhances chain mobility, enabling lower‐temperature gelation suitable for on‐skin application. In addition to thermal modulation, DES improves ionic conductivity by providing mobile ions (Cl^−^) and enhancing electronic conductivity via partial phase separation between PEDOT^+^ and PSS^−^. This phase separation facilitates PEDOT aggregation and π–π stacking interactions, which synergistically enhance the electronic conductivity. The resulting biogel achieves robust skin integration and high conductivity based on the dual ionic and electronic conduction pathway, enabling stable ECG and surface EMG (sEMG) recordings, even during vigorous body motions such as shaking, walking, and running. The incorporation of ionic materials such as DES thus enables simultaneous modulation of the rheological phase transition behavior and conductivity of the biogel, optimizing its performance for wearable electronic applications.

Hydrogels are considered ideal interface materials for on‐skin electrodes due to their low modulus and excellent biocompatibility.^[^ ^184^, ^208^ ^]^ However, their inherently high‐water content poses a major challenge, as they are prone to dehydration over time,^[^ ^308^ ^]^ which results in increased stiffness and reduced ion mobility, ultimately compromising electrical performance of the hydrogel. Consequently, improving the anti‐dehydration properties of hydrogels is a key requirement for their practical application, particularly in skin‐adhesive systems that demand long‐term stability and reliable performance. To address this issue, He et al. developed an anti‐dehydration hydrogel electrode by incorporating biocompatible zwitterionic oligomers into a hydrogel matrix, achieving excellent water retention capability (Figure 10d).^[^ ^119^ ^]^ A stretchable and adhesive hydrogel network was constructed through the copolymerization of acrylamide and 2‐methacryloyloxyethyl phosphorylcholine (MPC) (P(AAm‐co‐MPC)). Subsequently, a drying–swelling process was employed to embed MPC oligomers (MPC_10_) into the hydrogel, resulting in a functional anti‐dehydration hydrogel. Molecular dynamics (MD) simulations revealed that MPC_10_ forms multiple robust hydrogen bonds with surrounding water molecules, effectively suppressing water evaporation. These anti‐dehydration hydrogels were applied as bioelectronic interfaces for long‐term ECG monitoring. Unlike conventional PAAm hydrogels, which completely failed to record signals after 24 h due to conductivity loss from dehydration, the anti‐dehydrated hydrogels retained their conductive properties and delivered stable and continuous ECG signal. The incorporation of zwitterion components thus provides an effective strategy to mitigate dehydration, enabling hydrogel‐based electrodes to maintain electrical performance and signal fidelity over extended durations.

Ionic wearable electrodes have demonstrated remarkable potential in achieving robust integration with biological surfaces by offering enhanced mechanical performance,^[^ ^302^ ^]^ conformal contact,^[^ ^143^ ^]^ and efficient charge conduction.^[^ ^275^, ^309^ ^]^ Specifically, reducing mechanical impedance ensures better compliance with soft, deformable tissues, while lowering chemical impedance enhances interfacial charge transport and reduces signal degradation. Beyond impedance reduction, future improvements in ionic wearable electrodes should also address challenges in long‐term structural stability under physiological conditions, as well as integration of multimodal functions such as sensing, stimulation, and drug delivery. Furthermore, to achieve reliable biosignal monitoring in motion‐rich environments, ionic electrodes must be engineered to minimize motion artifacts and maintain high signal resolution under dynamic mechanical stress. Continued efforts in material design, fabrication scalability, and physiological validation are expected to accelerate the real‐world translation of ionic wearable electronics as next generation biointerface technologies.

### 3.3 Ionic Interfaces

With the growing demand for applications such as healthcare monitoring, disease diagnosis, and human‐machine interfaces, there has been a surge of interest in developing bioelectronic devices that can interface effectively with biological tissues. Traditionally, most electronic devices have relied on materials that detect and transmit signals via electric conduction. However, biological systems operate through ionic conduction, such as neural signals that rely on ion movement through ion channels.^[^ ^193^, ^310^, ^311^, ^312^ ^]^ This discrepancy in signal carriers, electrons in devices versus ions in biological systems, create fundamental challenges at the device‐tissue interface. In particular, it results in mismatched propagation speeds and poor mutual signal compatibility, leading to interfacial bottlenecks. To prevent such issues, material design should consider key parameters such as electronic mobility and volumetric capacitance, which govern signal amplification and dissipation.^[^ ^313^ ^]^ Otherwise, these mismatches can cause several issues, including high interfacial impedance at low frequencies, significant noise and errors in signal transduction, and substantial voltage drop across the interface.^[^ ^276^, ^314^, ^315^ ^]^ As a result, the performance, sensitivity, and reliability of bioelectronic devices are significantly limited when interfacing with living tissues.

To overcome these limitations in signal transduction across the device–tissue interface, recent efforts have focused on developing materials that bridge the gap between electric and biological systems. Ion‐conductive materials based on hydrogels or elastomers offer improved physiological compatibility by enabling ionic signal transmission and reducing impedance and signal loss.^[^ ^22^, ^300^, ^314^, ^315^, ^316^, ^317^, ^318^ ^]^ Another strategy involves composite materials that combine ionic and electronic conduction within a single system, enhancing the fidelity of signal transduction between bioelectronic devices and tissues.^[^ ^210^, ^276^, ^319^ ^]^

**Figure 11:**
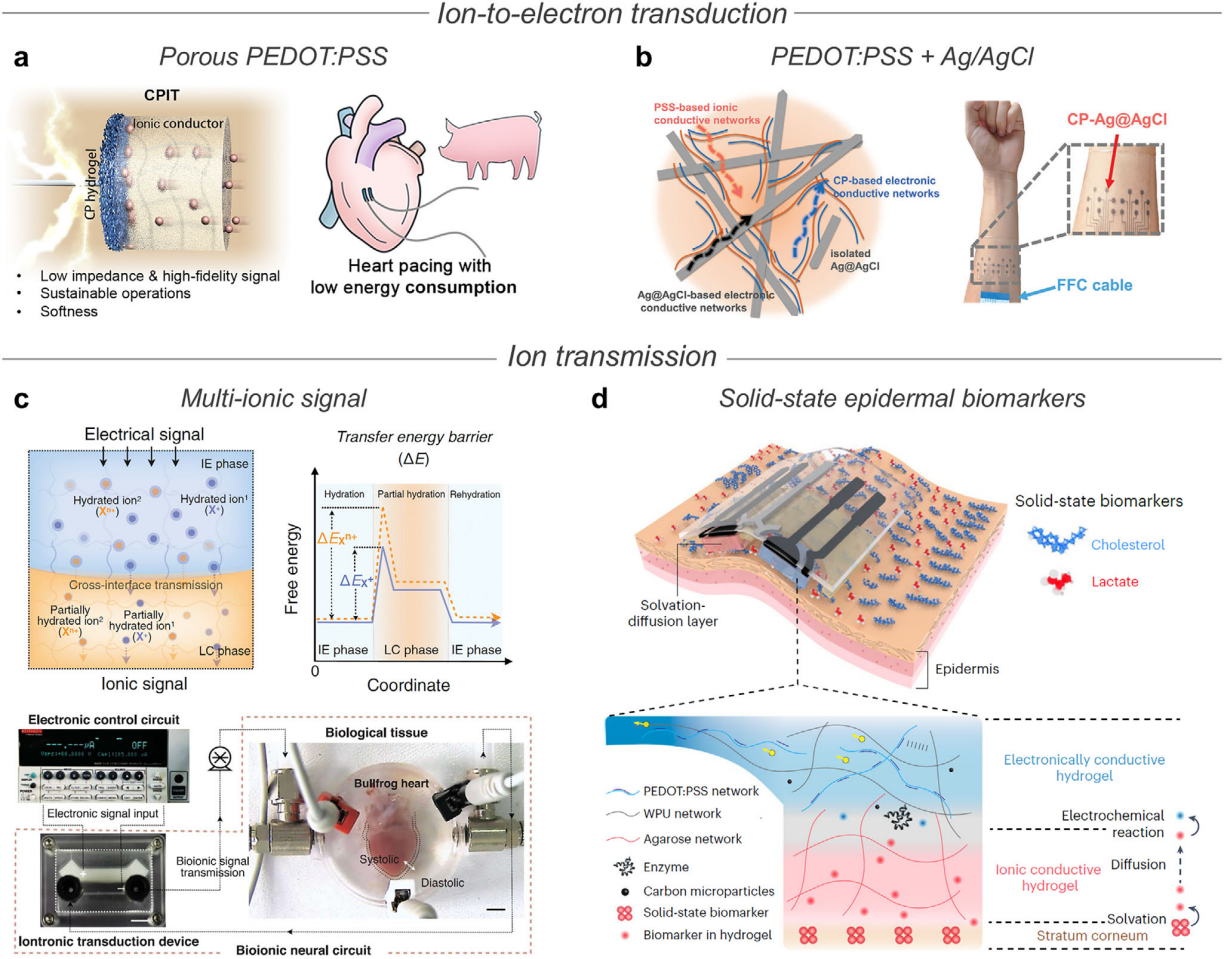
Ionic interfaces for bioelectronics. a) PEDOT:PSS hydrogel electrodes for pacing a porcine heart. Reproduced with permission.^[^ ^314^ ^]^ Copyright 2022, Elsevier Inc. b) PEDOT:PSS–Ag/AgCl electrode with mixed ionic‐electronic conductivity, demonstrating sEMG recording using an electrode array. Reproduced with permission.^[^ ^276^ ^]^ Copyright 2024, Wiley‐VCH. c) Heterogated cross‐interface ion transmission, enabling diverse electronic‐to‐multi‐ionic signal conversion based on distinct ion transfer energy barriers (∆E) between X^+^ and X^n+^ ions. Demonstration of neurohumoral signal modulation. Reproduced with permission.^[^ ^231^ ^]^ Copyright 2023, The American Association for the Advancement of Science. d) SEB sensor on the skin, with a cross‐sectional view illustrating the layered structure. Reproduced with permission.^[^ ^277^ ^]^ Copyright 2024, Springer Nature.

PEDOT:PSS is a representative OMIECs, capable of both electron conduction and ion transport,^[^ ^192^ ^]^ a feature that has led to their extensive use in biointerface applications.^[^ ^193^, ^276^, ^314^ ^]^ To further enhance signal transduction at the device‐tissue interface, introducing porosity into PEDOT:PSS has proven effective.^[^ ^320^, ^321^ ^]^ The porous structure increases the effective surface area, boosting EDL capacitance and enabling stronger capacitive charge injection.^[^ ^322^, ^323^, ^324^ ^]^ However, increased porosity can compromise mechanical stability. Addressing this challenge, Yao et al. developed a nanoporous PEDOT:PSS hydrogel electrode with high tensile strength (≈30 MPa) and reduced low‐frequency impedance, achieved through surface gelation, sulfuric acid crosslinking, and partial densification (**Figure** **11**a).^[^ ^314^ ^]^ These modifications overcome the intrinsic low tensile strength (e.g., 0.1–2 MPa) of untreated PEDOT:PSS. Controlling water evaporation further shrinks micropores into nanopores, enhancing volumetric capacitance and EDL‐based capacitive charge injection. Electrically, the hydrogel exhibited a comparable high‐frequency impedance to conventional inert metal electrodes, with minimal change from 10^5^ to 1 Hz, outperforming inert metal electrodes that show ≈1000‐fold increase of impedance at low frequencies. Biologically, the electrode promoted fibroblast proliferation under electrical stimulation and enabled cardiac pacing in a porcine heart at a threshold of 0.4 V, significantly lower than gold (1.6 V) and platinum/carbon (1.0 V) electrodes. Despite its excellent performance, the low fracture strain of PEDOT:PSS remains a limitation for applications on highly deformable or curved tissue.

While PEDOT:PSS has emerged as a promising material for biointerface recording, conventional EXG (ECG, EMG, and EEG) signals are typically acquired using Ag/AgCl electrode with electrolytic interfaces. These electrodes facilitate faradaic reactions and maintain stable potentials, which are beneficial for suppressing artifacts in ECG signal such as defibrillation currents from pacemakers during their operation, as well as the cardiac condition. However, their rigid structure leads to nonlinear frequency response and low interfacial stability, ultimately compromising signal transmission accuracy.^[^ ^276^, ^279^, ^314^ ^]^ To leverage the complementary advantages of both Ag/AgCl and PEDOT:PSS, Hu et al. developed a hybrid bioelectrode combining Ag/AgCl and PEDOT:PSS to form a mixed ionic–electronic conduction pathway with bidirectional ion‐to‐electron transduction capability (Figure 11b).^[^ ^276^ ^]^ This composite structure facilitates self‐adaptive optimization of ionic and electronic transport in response to input frequency, enhancing transduction efficiency across a broad bandwidth. At high frequencies (10^5^ Hz), it shows low impedance (≈34 Ω) due to the high conductivity of AgNWs. In the mid‐frequency range, the 3D nanostructured Ag@AgCl framework promotes rapid ion transport, reducing resistance compared to pure PEDOT:PSS. At low frequencies, it retains capacitive behavior with ultralow impedance and high areal capacitance (0.28 F m^−2^), supporting efficient charge storage. Additionally, the faradaic properties of Ag/AgCl contributes to a stable open‐circuit potential over 10 h, improving signal stability by reducing DC offset. Functionally, the electrode enabled sEMG mapping of five hand gestures and detected steady‐state visual evoked potentials from the prefrontal cortex, enabling a brain‐computer interface that facilitated human‐machine collaborative control of robot navigation. Moreover, it supported charge injection for neuromuscular stimulation, highlighting its multifunctional capability. However, the potential cytotoxicity of Ag/AgCl remains a concern for long‐term implantation and must be addressed in future designs.^[^ ^325^, ^326^ ^]^

Building on recent advances in bioelectric signal detection, the ionic interfaces have expanded beyond simple signal acquisition to include signal transmission to biological tissues and the detection of chemical biomarkers on the skin. However, most existing iontronic systems rely on single‐species ions as signal carriers,^[^ ^41^, ^327^, ^328^ ^]^ whereas biological neurons utilize multiple ion species to transmit diverse signals via action potentials.^[^ ^231^, ^310^, ^311^ ^]^ This constraint limits the signal diversity and functional complexity, driving interest in platforms capable of transmitting multiple ionic species. To demonstrate electronic‐to‐multi‐ionic signal transmission, Chen et al. developed a cascade‐heterogated biphasic gel (HBG) that enables voltage‐controlled, cross‐interface transport of multivalent cations (Figure 11c).^[^ ^231^ ^]^ The HBG features a phase‐separated heteronetwork: an ion‐hydrating polyacrylamide hydrogel and an ion‐dehydrating organogel composed of PMMA and poly(lauryl methacrylate). Under an electric potential, ions encounter distinct cross‐interface transfer energy barriers, influenced by charge density, size, hydrogen bonding, and the hydration shell stability. Theoretically, ions with higher charge densities lead to the strong electrostatic interaction with water molecules to stabilize their hydration shells, resulting in higher cross‐interface transfer energy barriers ($\Delta E_{Fe^{3+}}$ > $\Delta E_{Ca^{2+}}$ > $\Delta E_{K^{+}}$).^[^ ^329^ ^]^ These energy differences result in different voltage thresholds for various multivalent cations, enabling electronic‐to‐multi‐ionic hierarchical transmission and selective ionic cross‐stage transmission. Functionally, the HBG modulated the cardiomyocyte permeability and restored ECG rhythms in bullfrog hearts by delivering K^+^ under low‐voltage stimulation (5 mV, 5 Hz), mimicking neurohumoral signaling. In contrast, a higher voltage (100 mV) preferentially transmitted Ca^2+^, inducing abnormal cardiac responses. While this approach enables programmable, multispecies ion transmission, it is currently limited to cations of different valences. Future studies should explore selective control over anions and similarly charged cations such as Na^+^ and K^+^, which are central to physiological signaling.

In addition to electric biosignals, continuous monitoring of biomarkers in biofluids offer valuable information for healthcare and disease diagnostics. However, conventional approaches often rely on exercise‐induced sweat or invasive procedures, limiting accessibility.^[^ ^277^, ^330^, ^331^, ^332^ ^]^ Solid‐state epidermal biomarkers (SEBs) such as solid‐state cholesterol, lactate and glucose in stratum corneum represent a promising class of analytes for non‐invasive, in situ, and continuous health monitoring. SEB sensing does not require sweat stimulation or invasive procedures such as venipuncture.^[^ ^277^, ^332^ ^]^ However, in situ detection remains challenging due to low reaction kinetics in the solid phase and the absence of compatible ionic and electronic pathways in conventional electrodes. Traditional SEB sampling methods involve invasive or minimally invasive procedures, including skin biopsy or tape stripping, which are poorly tolerated by patients, and could induce an infection.^[^ ^332^ ^]^ Although non‐invasive techniques exist, they typically require additional steps (e.g., extraction, purification, and pre‐concentration), increasing cost and time. Furthermore, detection methods like liquid chromatography‐mass spectrometry or enzyme‐linked immunosorbent assay rely on complex instrumentation, time‐consuming and professional operation.^[^ ^332^ ^]^ To address these limitations, Arwani et al. developed stretchable ionic–electronic bilayer hydrogel electronics capable of direct, in situ SEB detection via solvation and diffusion of SEBs utilizing ionic interface (Figure 11d).^[^ ^277^ ^]^ The ionic conductive hydrogel (ICH) forms a solvation‐mediated ionic conduction pathway from the stratum corneum to the electrochemical sensing layer. Basically, artificial eccrine perspiration is used as the solvent for the ICH, with Triton X‐100 and ethanol added to solubilize and emulsify water‐insoluble analytes like solid‐state cholesterol. Once SEBs are dissolved and diffused in the ICH matrix, they undergo enzyme‐mediated redox reactions, and the resulting signal are transduced through a waterborne polyurethane (WPU)/PEDOT:PSS‐based electronically conductive hydrogel (ECH) layer. The sensor demonstrated high sensitivity to solid lactate (93.8 nA nmol^−1^ cm^−2^) and solid cholesterol (201.34 nA nmol^−1^ cm^−2^), with low detection limit of 0.51 nmol cm^−2^ and 0.26 nmol cm^−2^, respectively. Device performance was validated against both tape‐stripped SEB samples analyzed by mass spectrometry and serum biomarkers from a commercial finger‐prick method, showing strong linear correlation and reproducibility. This platform highlights a universal approach to non‐invasive biomarker monitoring without the need for biofluid acquisition, offering future potential in areas such as drug screening and food safety. Nevertheless, long‐term hydration stability of hydrogel‐based ionic interfaces remains a key challenge for practical deployment.

The development of ionic interfaces has significantly advanced the field of bioelectronics by addressing the fundamental mismatch between electronic devices and biological tissues (**Table** **6**). Innovations in ion‐conductive hydrogels and hybrid electrode architectures have enabled high‐fidelity signal transduction,^[^ ^276^ ^]^ reduced interfacial impedance,^[^ ^314^ ^]^ and expanded the functional scope of bioelectronic devices.^[^ ^231^, ^277^ ^]^ Nevertheless, several key challenges remain for next‐generation ionic interfaces, including improving long‐term biocompatibility and stability in wearable applications, and exploring diverse ion species to enhance biosignal specificity and communication. Future work should focus on the development of tough, self‐healing, and anti‐dehydrating biocompatible materials to support long‐term and repeatable use in dynamic biological environments.^[^ ^333^, ^334^, ^335^ ^]^ Additionally, expanding the range of signal carriers, such as anions and similarly charged cations, may better match the complex signaling characteristics of biological systems and enable multi‐ionic bioelectronic communication.^[^ ^336^, ^337^ ^]^

**Table 6:** Ionic interfaces.

Functions of ionic species	Ionic species	Ionic material^a)^	Polymer matrix^b)^	Applications	References
Ion‐to‐electron transduction	OMIEC	PEDOT:PSS	Hydrogel (PEDOT:PSS)	Heart pacing electrode	[314]
		PEDOT:PSS	PET, Ecoflex	ECG, EEG, sEMG electrode	[276]
Ion conducting interfacial layer	OMIEC	Hydrophobic‐treated PEDOT:PSS (HT‐PP)	‐	K^+^ sensor	[319]
	Ionic polymer	PMPC	Zwitterionic polymer (PMPC)	ECG, EMG electrode	[318]
	Ion	NaCl, KCl	Hydrogel (HA)	EEG electrode	[210]
		CaCl_2_	Hydrogel (plasticized silk)	EMG electrode	[317]
		LiCl	Hydrogel (alginate–PAAm)	sEMG electrode	[22]
		NaCl	Organohydrogel (P(AM‐co‐AA))	ECG, EMG, auditory brainstem response monitoring electrode	[315]
Signal transmission	Ion	K^+^, Ca^2+^, Fe^3+^	Biphasic gel (PAAm, PMMA/PLMA)	Neurohumoral modulation	[231]
Biomarker	Solvated molecule	Solvated biomarkers (lactate)	Hydrogel (agarose, gelatin)	Solid‐state biomarker sensor	[277]
		Solvated biomarkers (serine)	Hydrogel (PVA)		[332]

^a)^ pDMC: poly(acryloyloxyethyltrimethylammonium chloride, pSMA: poly(sodium methacrylate)

^b)^ PET: polyethylene terephthalate, (PMPC: poly(2‐methacryloyloxyethyl phosphorylcholine), P(AM‐co‐AA): poly(acrylamide‐co‐acrylic acid), PLMA: poly(lauryl methacrylate)

## 4 Iontronic and OECT‐Based Sensors

Real‐time monitoring of physiological signals and biomarkers is essential for early diagnosis and personalized healthcare. Sensors based on ionic materials are well‐suited for these applications, offering high sensitivity, low power operation, and conformal integration with soft biological tissues. These sensors are generally classified into two types: iontronic sensors, typically sandwich‐type structures, where an electrolyte is placed between two electrodes, and OECTs, which consist of source, drain, and gate electrodes with an electrolyte layer and a semiconducting channel.

**Figure 12:**
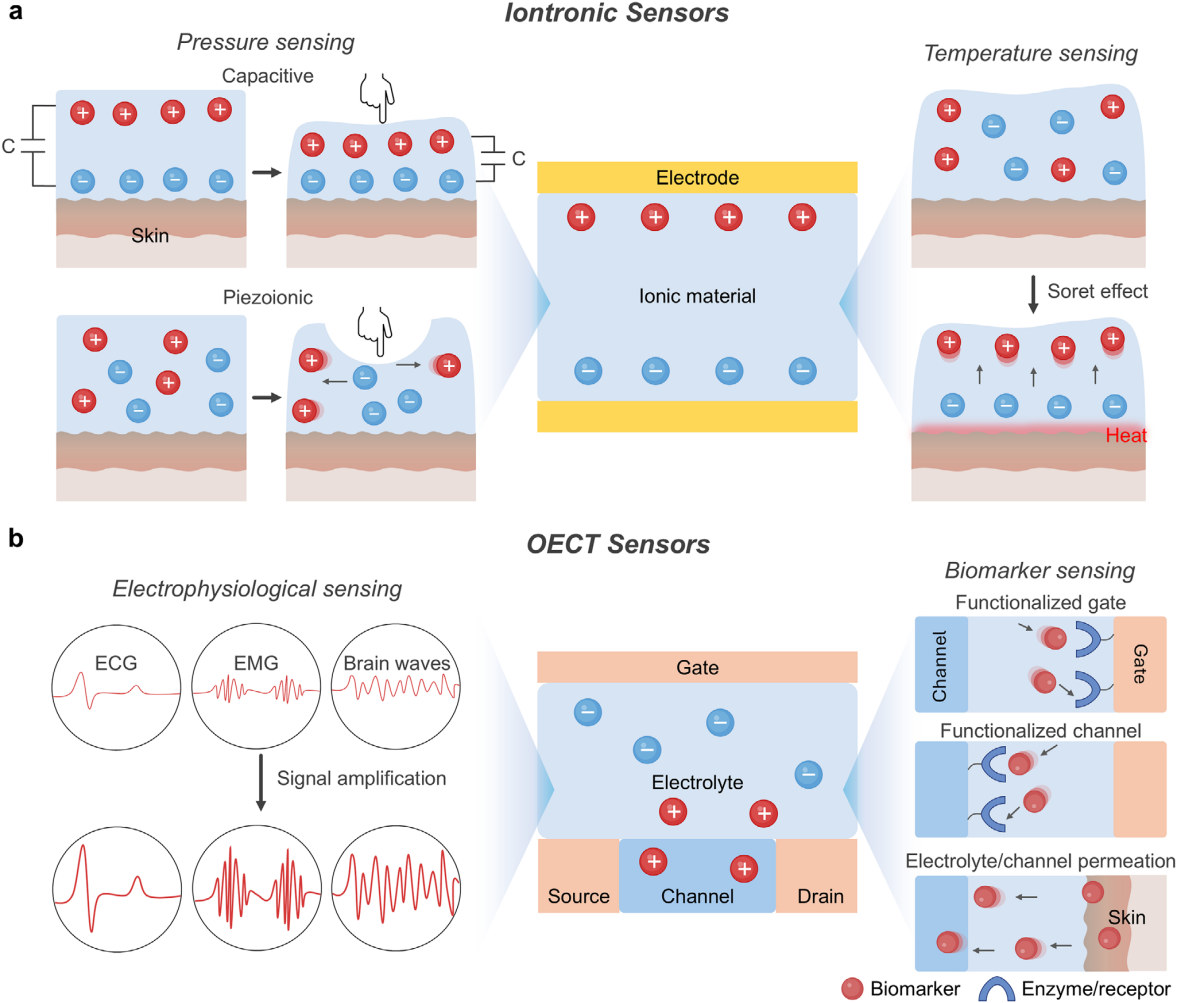
Iontronic sensors and OECT‐based sensing mechanisms for bioelectronics. a) Iontronic sensors detect pressure via capacitive (EDL modulation) or piezoionic (ionic polarization) mechanisms, and temperature through ion thermodiffusion driven by the Soret effect. b) OECT‐based sensors amplify low‐amplitude electrophysiological signals (ECG, EMG, brain waves) and detect biomarkers via ion penetration or specific molecular interactions at the gate or channel, offering multimodal biosensing capabilities.

Iontronic sensors respond to external stimuli through selective ion migration and polarization (**Figure** **12**a). Based on their sensing mechanisms, pressure sensors are classified into capacitive and piezoionic types. Capacitive sensors utilize EDL formation at the electrolyte–electrode interface under applied voltage. Pressure alters the electrode spacing and contact area, modulating EDL charge density and capacitance. Piezoionic sensors function without external power, relying on ionic polarization generated by mechanical deformation and differences in ion mobility.^[^ ^65^, ^328^, ^338^, ^339^, ^340^ ^]^ For temperature sensing, iontronic devices use the Soret effect, where a temperature gradient induces differential migration of cations and anions, resulting in ionic polarization and a measurable electrical output.^[^ ^29^, ^341^, ^342^ ^]^ Unlike conventional sensors that require external amplification, OECTs directly convert local field potential changes from organs or tissues into amplified channel currents through their high transconductance, enabling precise detection of low‐amplitude biosignals such as ECG, EMG, EOG, and brain waves (Figure 12b).^[^ ^343^ ^]^ Beyond electrical signal amplification, OECTs also support biomarker sensing by converting ionic and biochemical inputs into electrical signals.^[^ ^30^ ^]^ Through mechanisms such as ion migration in the electrolyte, ion penetration into the channel,^[^ ^344^ ^]^ or interactions between metabolites and functionalized gates^[^ ^345^ ^]^ or channels,^[^ ^346^ ^]^ OECTs enable the detection of diverse biomarkers, providing a versatile platform for wearable biosensing applications. Together, iontronic sensors and OECTs offer complementary capabilities for continuous, skin‐conformal monitoring, enabling multiplexed detection of pressure, temperature, electrophysiological signals, and biomolecular markers for real‐time clinical assessment and personalized therapeutic strategies.

### 4.1 Iontronic Sensors

With the rapid advancement of digital healthcare technologies, personal health monitoring and remote diagnostics have become increasingly important.^[^ ^347^, ^348^ ^]^ This trend drives demand for wearable sensors capable of continuous, real‐time monitoring of physiological signals while remaining compact, sensitive, and mechanically compatible with soft tissues.^[^ ^349^, ^350^, ^351^ ^]^ Among various physiological signals, pressure is particularly vital, as it reflects key indicators such as blood pressure,^[^ ^352^ ^]^ pulse,^[^ ^24^, ^25^, ^353^, ^354^, ^355^ ^]^ and respiratory activity.^[^ ^356^, ^357^ ^]^ Capacitive pressure sensors, which detect mechanical deformation via capacitance changes, have been widely used due to their simple structure, fast response, and low hysteresis.^[^ ^358^ ^]^ However, their sensitivity is limited by the low compressibility of solid dielectric layers,^[^ ^359^, ^360^ ^]^ and their rigid components hinder conformity to soft tissues, causing unstable contact and reduced accuracy.^[^ ^297^ ^]^ Furthermore, their reliance on electron‐based signal transmission—unlike the ionic nature of biological signals—requires complex amplification circuits and increases power consumption.^[^ ^12^ ^]^ These limitations hinder their effectiveness in detecting subtle physiological changes, which are critical for accurate health monitoring.^[^ ^361^ ^]^ In contrast, iontronic sensors form EDLs at the electrode–electrolyte interface, where subtle mechanical or thermal stimuli induce significant capacitance changes.^[^ ^362^ ^]^ Surface microstructure engineering further boosts sensitivity by increasing EDL contact area and localizing electric field.^[^ ^358^, ^363^ ^]^ These features support high‐resolution, low‐power physiological monitoring and enable early disease detection and health risk prediction through continuous data analysis.

**Figure 13:**
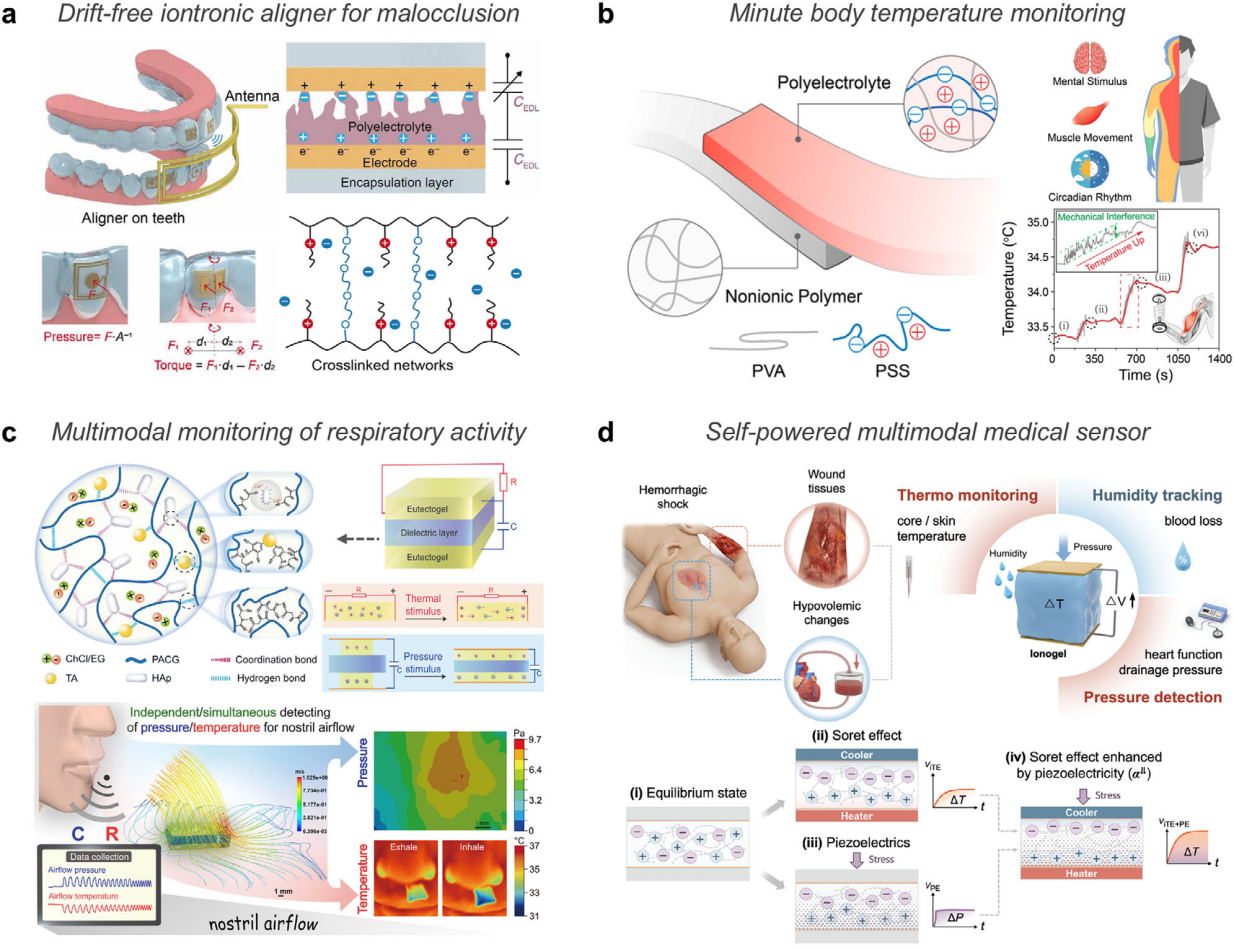
Iontronic sensors for smart healthcare applications. a) Wireless monitoring of teeth alignment using a drift‐free iontronic sensor. Reproduced with permission.^[^ ^144^ ^]^ Copyright 2025, The American Association for the Advancement of Science. b) Asymmetrical polymer bilayers for monitoring minute temperature changes induce by physiological activities. Reproduced with permission.^[^ ^28^ ^]^ Copyright 2024, American Chemical Society. c) Eutectogel‐based multimodal sensor for real‐time respiratory monitoring to diagnose respiratory disease. Reproduced with permission.^[^ ^27^ ^]^ Copyright 2025, Springer Nature. d) Self‐powered medical sensor based on piezoelectric‐enhanced thermoelectric ionogels for monitoring of temperature, pressure, and humidity. Reprinted with permission.^[^ ^29^ ^]^ Copyright 2025, Wiley‐VCH GmbH.

While iontronic pressure sensors have been widely applied for physiological monitoring such as EMG^[^ ^233^, ^364^ ^]^ and pulse detection,^[^ ^24^, ^25^, ^354^, ^355^ ^]^ their use in orthodontic monitoring remains limited despite the clinical importance of monitoring sustained forces during tooth alignment. Accurate force monitoring is essential to prevent tissue damage and ensure timely treatment adjustments, but conventional sensors often have stiff structures^[^ ^365^, ^366^ ^]^ or require wired connections.^[^ ^367^ ^]^ Orthodontic must detect high static pressures (up to ≈100 kPa) over prolonged periods. While flexible thin‐film sensors have been integrated into tooth aligners, their soft active materials are prone to viscoelastic creep, leading to signal drift and reduced accuracy.^[^ ^368^ ^]^ Microstructured iontronic sensors offer high sensitivity but still suffer from creep and ion leakage, leading to long‐term signal instability and biosafety concerns.^[^ ^369^ ^]^ Additionally, integrating iontronic sensors into wireless systems is hindered by uncontrolled ion dissociation, compromising signal reliability. To overcome these limitations, Song et al. developed a wireless iontronic sensor using a creep‐ and leakage‐free polyelectrolyte, poly (1‐vinyl‐3‐butylimidazolium bis(trifluoromethylsulfonyl)imide) (P(VBIM‐TFSI)) (**Figure** **13**a).^[^ ^144^ ^]^ The short‐chain, highly crosslinked polyelectrolyte network minimizes interchain friction, suppressing creep under pressures up to 650 kPa. Ion leakage is prevented by trapping mobile ions within the polymer chains, stabilizing EDL contact and minimizing signal drift.^[^ ^368^ ^]^ This material was integrated into a wireless inductance‐capacitance (LC) resonant sensor embedded in a tooth aligner. During orthodontic correction, applied forces and torques modulate capacitance at one or more sensing sites, modulating the resonance frequency of the LC circuit. Therefore, this wireless iontronic sensor allows non‐invasive, real‐time readout of mechanical load applied to the teeth. The creep‐ and leakage‐free properties ensured sharp, stable resonance peaks, enabling continuous and accurate tracking of tooth movement.

Pressure is a widely used physiological indicator due to its direct mechanical response, but subtle temperature changes can precede pressure‐related symptoms, particularly in circulatory and metabolic abnormalities.^[^ ^370^, ^371^, ^372^ ^]^ Even under normal conditions, temperature changes reflect key physiological activities such as exercise, stress responses, and circadian rhythms.^[^ ^373^ ^]^ High‐resolution temperature sensing therefore complements pressure monitoring, enabling more comprehensive health assessment. However, such thermal changes typically occur at the millikelvin (mK) level, beyond the detection limit of conventional sensors, which are generally limited to ≈100 mK.^[^ ^374^, ^375^, ^376^ ^]^ This necessitates the development of ultrasensitive sensors capable of detecting sub‐mK variations for accurate physiological monitoring. Addressing this challenge, Li et al. developed a flexible temperature sensor inspired by biological thermoreceptors (Figure 13b).^[^ ^28^ ^]^ The device features an asymmetric polymer bilayer (APB) sensor employs a temperature‐dependent cation injection mechanism at the interface of non‐ionic polymer PVA and polyelectrolyte sodium polystyrene sulfonate (PSS). The PVA layer initially shows high resistance due to the lack of mobile ions. As temperature increases, sodium ions dissociate from the PSS layer and migrate into the PVA layer, increasing conductivity and reducing resistance. Upon cooling, the sodium ions return to the PSS layer, restoring the high‐resistance state of the PVA layer. This reversible, temperature‐dependent cation redistribution, which is governed by PSS ionization, sodium ion mobility, and PVA chain dynamics, enables precise electrical responses to minimal thermal stimuli (1.42 mK). In wearable healthcare monitoring, the APB sensor effectively tracked localized temperature increases during exercise, forehead temperature variations under mental stress, and circadian body temperature during sleep, demonstrating its potential for real‐time, non‐invasive health monitoring.

Continuous respiratory is essential for health assessment, as breathing patterns reveal both physical and mental conditions, with abnormalities often indicating early‐stage health issues.^[^ ^377^ ^]^ In particular, obstructive sleep apnea syndrome (OSAS), characterized by repeated breathing interruptions during sleep, is associated with cardiovascular disease, chronic fatigue, and cognitive decline.^[^ ^26^ ^]^ Accurate and real‐time monitoring of respiratory patterns is therefore vital for timely diagnosis and treatment. However, most existing respiratory monitoring technologies rely on detecting a single physiological signal, making them prone to motion artifacts and environmental noise, which can lead to unreliable assessments or missed apnea events.^[^ ^26^, ^378^ ^]^ To overcome these limitations, sensors should simultaneously and independently detect multiple physiological signals with minimal signal interference, while ensuring skin conformity, biocompatibility, environmental stability, and seamless integration into wearable devices for continuous use. Addressing these challenges, Liu et al. developed a multimodal sensor integrating temperature and pressure sensing within a single platform, enabling accurate and crosstalk‐free respiratory monitoring (Figure 13c).^[^ ^27^ ^]^ The sensor is based on a biocompatible poly (NAcryloyl 2‐Glycine) eutectogel composed of ChCl/ethylene glycol (EG)‐based DES and a physically crosslinked polymer network incorporating hydroxyapatite (HAp) and tannic acid (TA). The dual‐mode sensor features a sandwiched structure with a VHB dielectric layer positioned between two eutectogels. First, temperature is measured via resistance changes in one eutectogel layer. Increased temperature accelerates choline cations and chloride anions and promotes Ca^2+^ dissociation from HAp, increasing free ion concentration and conductivity. This mechanism enables sensitive thermal detection with a temperature coefficient of resistance (TCR) of −1.67 % °C^−1^ over 20–35 °C. In parallel, nostril airflow‐induced pressure is detected via capacitance changes from distance fluctuations between the two eutectogel layers, achieving a sensitivity of 0.42 kPa^−1^ and a detection limit below 10 Pa. By simultaneously capturing both temperature and pressure signals, this sensor enables accurate and comprehensive monitoring of respiratory activity. Its eutectogel‐based design also provides long‐term stability, self‐healing ability, and strong skin adhesion, making it well‐suited for wearable devices that continuously track breathing patterns under daily conditions.

Although bi‐ and multimodal iontronic sensors have been developed to detect various stimuli, they often rely on separate transduction mechanisms and external power supplies, limiting integration and scalability.^[^ ^27^, ^350^, ^379^ ^]^ To address these limitations, recent approaches utilize ambient stimuli such as pressure,^[^ ^65^, ^380^ ^]^ humidity,^[^ ^381^, ^382^ ^]^ and temperature,^[^ ^383^ ^]^ not only as sensing inputs but also as energy sources, enabling self‐powered sensing via stimuli‐induced voltage or capacitance changes through ion redistribution. Integrating self‐powered functionality with multimodal sensing offers a robust platform for efficient, continuous health monitoring.^[^ ^384^ ^]^ Pai et al. demonstrated a self‐powered multimodal medical sensor based on a piezoelectric‐augmented thermoelectric ionogel (i‐TE ionogel) capable of simultaneously detecting temperature, pressure and humidity (Figure 13d).^[^ ^29^ ^]^ The sensor, composed of poly(vinylidene fluoride‐co‐hexafluoropropylene) (PVDF‐HFP) and [EMIM][TFSI], utilizes a piezoelectric‐enhanced i‐TE mechanism. Mechanical pressure induces an internal electric field via the piezoelectric PVDF‐HFP, which enhances directional ion migration under a temperature gradient, amplifying the Soret effect. This synergistic coupling of mechanical and thermal stimuli boosts thermovoltage output from ambient inputs, enabling fully self‐powered operation. The sensor exhibits a temperature sensitivity of 0.96 mV K^−1^, pressure sensitivity of 0.13 mV kPa^−1^ within 5.3–24.0 kPa, and humidity sensitivity of 0.033 mV K^−1^ %RH^−1^, with fast response and stable performance over 1000 cycles. To evaluate its clinical utility, the sensor was applied to monitor hemorrhagic shock, characterized by changes in body temperature, blood volume, and tissue pressure. Two ionogel‐based devices, a catheter‐type for internal monitoring and a sponge‐integrated type for surface tracking, enabled accurate, multimodal sensing without external power, demonstrating strong potential for practical deployment in power‐limited medical environments. Despite its potential, full signal independence is limited by cross‐sensitivity.

Iontronic sensors convert external stimuli into electrical signals via mobile ions that respond sensitively to pressure, temperature, as well as strain^[^ ^234^, ^385^ ^]^ and humidity^[^ ^382^, ^386^ ^]^ through chemical, electrical, and thermal gradients (**Table** **7**). These stimuli induce ion movement, which is transduced into electrical signals by changes in interfacial capacitance through EDL formation or by resistance variation due to ion redistribution. Leveraging these mechanisms, advances in material and device designs have enabled iontronic sensors with high sensitivity^[^ ^26^ ^]^ and low‐power operation without relying on complex amplification circuits.^[^ ^12^ ^]^ Despite these advantages, challenges remain, including signal crosstalk from broad ionic responsiveness, and long‐term signal drift from ion leakage and material creep, and biocompatibility concerns arising from the release of non‐biocompatible ions. Addressing these challenges requires the development of stimulus‐selective ionic systems, leakage‐resistant architectures, and mechanically robust materials, along with continued progress in material and signal processing to enable next‐generation, self‐powered health monitoring.

**Table 7:** Iontronic sensors for bioelectronic applications.

Sensing target	Mechanism	Ionic material^a)^	Polymer matrix^b)^	Applications^c)^	References
Pressure	Capacitive	[EMIM][DCA]	P(BA‐co‐PEGMA)	Deep breath, swallow, pulse monitoring	[362]
		[BMIM][PF_6_]	DFPU	Sleep apnea monitoring	[26]
		[EMIM][OTF]	PVDF‐HFP	FMG, sEMG monitoring	[233]
		[EMIM][TFSI]	PVDF‐HFP	Fingertip pulse monitoring	[25]
Temperature	Capacitive	H^+^, PO_4_^−^	PVDF‐HFP/PEO	Diagnosis of diabetic foot ulcer	[372]
	Resistive	Na^+^	PVA/PSS bilayer	Minor skin temperature monitoring in physiological processes	[28]
Strain	Stimuli‐driven ion transport modulation	EMIM^+^ (in [EMIM][SPA]), TFSI^−^ (in [METAC][TFSI])	PEG‐based BBEs	Finger flexion, eye blinking, ex vivo organ deformation monitoring	[234]
		FeCl_3_	TPU/PVA	Human breath, pulse monitoring	[385]
Humidity		NaCl	Bacterial cellulose/sodium alginate	Respiratory activities monitoring	[382]
		LiCl	TPU	Detection of transepidermal water loss from skin	[386]
Pressure, strain	Capacitive	Na^+^	PANa	ECG, EMG monitoring	[364]
Pressure, torque		VBIM^+^ (fixed charge), TFSI^−^ (mobile ion)	Crosslinked P(VBIM‐TFSI)	In vivo orthodontic pressure and torque measurement	[144]
Pressure, temperature	Capacitive, resistive	ChCl	Crosslinked poly (N‐Acryloyl 2‐Glycine)	Monitoring nostril airflow	[27]
Pressure, temperature, humidity	Piezoelectric, thermoelectric	[EMIM][TFSI]	P(VDF‐HFP)	In vivo hemorrhagic shock monitoring	[29]

^a)^ DCA: dicyanamide, OTF: trifluoromethanesulfonate, SPA: 3‐sulfopropyl acrylate, METAC: [2(methacryloyloxy)ethyl]trimethylammonium, VBIM: 1‐vinyl‐3‐butylimidazolium

^b)^ P(BA‐co‐PEGMA): poly(benzyl acrylate‐co‐poly(ethylene glycol) methyl ether methacrylate), DFPU: polyester diol enriched with alkyl side chains as the soft segment, non‐toxic chain extenders featuring fluorine moieties and isocyanates as the hard segment, BBE: bottlebrush elastomer, PANa: sodium polyacrylate

^c)^ FMG: force myography

### 4.2 OECTs

#### 4.2.1 OECTs for Electrophysiological Signal Recording

While iontronic sensors and electrodes have enabled electrophysiological recordings by transducing ionic fluctuations at the bioelectronic interface, their passive nature makes them susceptible to motion artifacts^[^ ^187^ ^]^ and often necessitates external amplification circuitry.^[^ ^387^ ^]^ In contrast, OECT‐based sensors have emerged as a powerful alternative,^[^ ^343^ ^]^ offering active signal amplification through volumetric ion‐to‐electron transduction within OMIECs.^[^ ^388^ ^]^ A typical OECT consists of gate, source, and drain electrodes, an organic semiconductor channel, and an electrolyte that bridges the gate and channel. In depletion‐mode OECTs, the channel is initially conductive due to the doped OMIEC (e.g., PEDOT:PSS), and applying a positive gate voltage drives cations into the channel, dedoping it and reducing the drain current (I_DS_). In contrast, enhancement‐mode OECTs start in a non‐conductive state; a negative gate voltage (V_G_) induces cation injection into the channel, increasing doping and thereby raising I_DS_. For bioelectrical signal detection, OECTs function as transconductance amplifiers, translating fluctuations in local electrical potential into modulations in I_DS_. This mechanism relies on ion flux‐induced variations in local field potentials generated by organ, tissue, or single‐cell activity.^[^ ^343^ ^]^ The transconductance (g_m_ = ΔI_DS_/ΔV_G_) of the OECT is a key performance metric, directly influencing its capacity to amplify weak signals. Higher g_m_ enhances the signal‐to‐noise ratio (SNR) and boosts detection sensitivity, making it critical for accurate electrophysiological monitoring. The g_m_ of OECT in the saturation region is expressed as,^[^ ^389^ ^]^

(1) $$g_{m}=\mu C^{*}\frac{Wd}{L}\left(V_{TH}-V_{G}\right)$$

where μ is the electronic carrier mobility, and C* is the capacitance of the channel per unit volume, *W* is channel width, *L* is channel length, *d* is channel thickness and *V_TH_* is the threshold voltage. In OECTs, the entire volume of the channel actively participates in the ion doping/de‐doping process, where the volumetric capacitance enables high g_m_ even at low operating voltages.^[^ ^390^ ^]^ This supports stable, high‐resolution monitoring of bioelectrical signals, such as EMG,^[^ ^391^, ^392^, ^393^, ^394^ ^]^ ECG,^[^ ^159^, ^395^, ^396^, ^397^, ^398^, ^399^, ^400^, ^401^, ^402^ ^]^ EEG^[^ ^389^, ^402^, ^403^, ^404^, ^405^ ^]^ and EOG,^[^ ^406^, ^407^, ^408^ ^]^ making OECTs particularly attractive for wearable and implantable neural interfaces (**Table** **8**).

**Table 8:** OECTs for electrophysiological sensing.

Signal type^a)^	Electrolyte layer^b)^	Channel^c)^	Other features	References
ECG	[Ch][MA]/levan polysaccharide	P3CPT	Side‐gate	[159]
	NaCl	p(C_4_‐T_2_‐C_0_‐EG)	Cofacial pair complementary inverter	[395]
	NaCl	p(g2T‐T)	Stretchable	[396]
	NaCl	p‐PEDOT‐APD	Nanoporous aerogel channel	[400]
	[EMIM][TFSI]	P3HT	Striped channel for LI‐assisted ion transport	[401]
EMG	NaCl	p‐type: PBBTL n‐type: BBL	Complementary inverter	[393]
EOG	NaCl	p(gDPP‐V)	Volatile/nonvolatile behaviors, complementary inverter	[407]
EEG	Internal ion (within the channel)	PEDOT:PSS/D‐sorbitol	Flexible	[403]
	Forehead	PEDOT:PSS	Transparent, flexible	[404]
LFP	Internal ion (within the channel)	Depletion mode: PEDOT:PSS/PEI Enhancement mode: PEDOT:PSS/D‐sorbitol	Vertical structure with H‐via, flexible	[418]
	External electrolyte	p‐type: PEDOT:PSS n‐type: p(C_6_NDI‐T)	p–n OED, flexible shank	[419]
LFP, SUA	External electrolyte	p‐type: PEDOT:PSS n‐type: p(g2T‐T)	Push‐pull and differential amplifiers, flexible shank	[420]
	Internal ion (within the channel)	PEDOT:PSS/PEI	Conformable probe, H‐via	[421]
ECoG	External electrolyte	PEDOT:PSS	Flexible, biodegradable	[422]
EEG, ECG	ChCl:EG‐based ionogel	PEDOT:PSS/P14[TFSI]	Stretchable	[402]
ECG, EEG, ECoG	External electrolyte	P(TII‐2FT)	Single component cofacial vertical OECT, flexible	[405]
ECG, EMG	External electrolyte	p(g2T‐Se)‐THP	Immune compatible	[394]
ECG, EMG, EOG	PBS	PEDOT:PSS	4‐terminal vertical Corbino structure, flexible	[408]
ECG, EMG, LFP	Internal ion (within the channel)	PEDOT:PSS/PEI	Internal ion‐gated, flexible	[392]
ECG, EOG, EEG, ECoG	External electrolyte	P(PyV)‐H	Semiconducting hydrogel channel	[423]

^a)^ SUA: single unit activity

^b)^ PBS: phosphate‐buffered saline

^c)^ P3CPT: poly[3‐(5‐carboxypentyl)thiophene‐2,5‐diyl], p(C_4_‐T_2_‐C_0_‐EG): donor‐acceptor polymer featuring a naphthalenetetracarboxylic diimide unit with hybrid alkyl‐glycol side chains and a triethylene glycol–substituted bithiophene unit, p(g2T‐T): poly(2‐(3,3′‐bis(2‐(2‐(2‐methoxyethoxy)ethoxy)ethoxy)‐[2,2′‐bithiophen]‐5)yl thiophene, APD: ambient pressure drying, P3HT: poly(3‐hexylthiophene), PBBTL: 6H‐pyrrolo[3,2‐b:4,5‐b’]bis[1,4]benzothiazine ladder, BBL: poly(benzimidazobenzophenanthrolinedione), p(C_6_NDI‐T): naphthalene‐diimide‐thiophene‐ (NDI‐T)‐ based semiconducting polymer with triethylene glycol side chains separated by a 6‐alkyl carbon spacer from the NDI, P14[TFSI]: N‐butyl, methylpyrrolidinium bis(trifluoromethylsulfonyl)imide, p(g2T‐Se): thiophene replaced by selenophene in p(g2T‐T) backbone, THP: triazole‐tetrahydropyran

**Figure 14:**
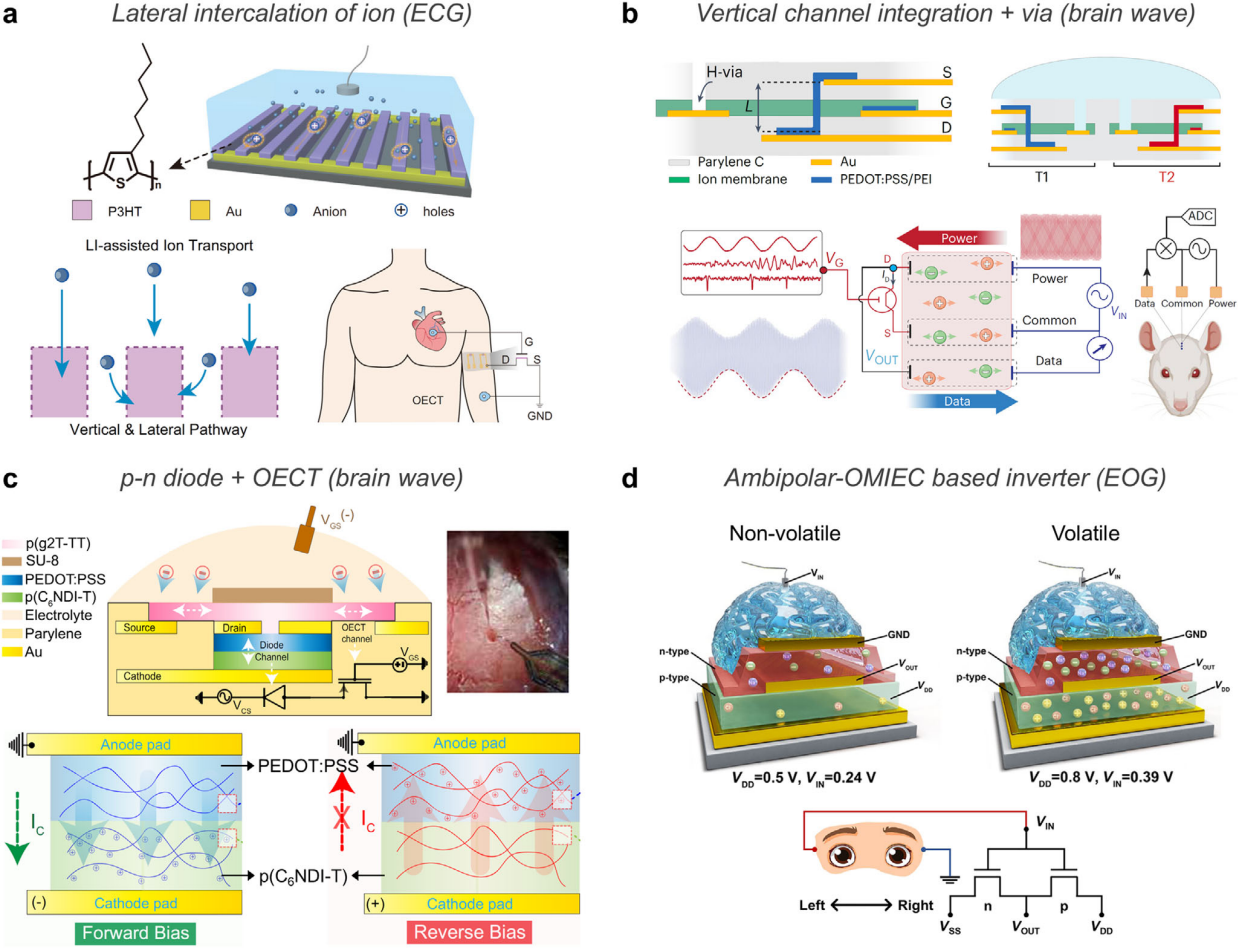
OECT‐based interfaces for electrophysiological signal recording. a) A striped‐channel OECT enabling lateral intercalation‐assisted ion transport for enhanced ECG recording. Reproduced with permission.^[^ ^401^ ^]^ Copyright 2024, Springer Nature. b) A vIGT architecture combining vertical channel orientation with a H‐via structure, enabling megahertz‐range brain signal recording. Reproduced with permission.^[^ ^418^ ^]^ Copyright 2023, Springer Nature under CC BY 4.0. license http://creativecommons.org/licenses/by/4.0/. c) A p–n junction OED integrated with an OECT for crosstalk suppression in brain signal recording. Reproduced with permission.^[^ ^419^ ^]^ Copyright 2024, Springer Nature under CC BY 4.0. license http://creativecommons.org/licenses/by/4.0/. d) An ambipolar OMIEC‐based inverter for EOG signal recording, exhibiting supply voltage‐dependent volatile and non‐volatile behaviors. Reproduced with permission.^[^ ^407^ ^]^ Copyright 2024, The American Association for the Advancement of Science.

ECG recording is one of the most extensively demonstrated applications of OECTs, as the low‐amplitude signals (≈mV) require local amplification for effective downstream processing.^[^ ^409^ ^]^ Moreover, weak signals from abnormal rhythms like fibrillation are easily masked by noise, requiring a high SNR for accurate detection.^[^ ^398^ ^]^ To capture such ECG signals accurately, the device must support a minimum sampling frequency exceeding 120 Hz,^[^ ^399^ ^]^ but the hydrophobic nature of organic semiconductors limits ion penetration through the EDL, resulting in sluggish transient responses. To address this, Yan et al. introduced an OECT with striped P3HT channel structure with a [EMIM][TFSI] electrolyte layer, where the striped design facilitates efficient ion exchange via lateral intercalation (LI)‐assisted transport (**Figure** **14**a).^[^ ^401^ ^]^ Unlike conventional architectures relying on vertical ion transport, the striped deign enables multidirectional (vertical and lateral) ion injection, shortening diffusion paths. As a result, OECTs with 2 µm strips showed a response time of 1.9 s, nearly ten times faster than 100 µm stripes (21.3 s), highlighting the effectiveness of lateral intercalation. Lateral intercalation also bypasses high crystalline barriers present in vertical ion movement, promoting uniform ion distribution, and lowering overall device resistance. As a result, g_m_ increased from 7.4 mS (100 µm) to 11.8 mS for (2 µm). In ECG recordings, the 2 µm channel showed lower noise and improved signal fidelity. While this approach significantly improves OECT response speed, it still remains slower than conventional Si‐based transistors with sub‐microsecond,^[^ ^410^ ^]^ indicating the need for further optimization for high‐speed electrophysiological recordings. In addition, the use of [EMIM][TFSI], which may pose potential toxicity,^[^ ^411^ ^]^ could raise biocompatibility concerns.

Brain signal recordings, whether subdural^[^ ^412^ ^]^ or deep‐brain,^[^ ^413^ ^]^ provide essential neurological information of cognitive behavior^[^ ^414^ ^]^ and are crucial for applications such as epilepsy monitoring^[^ ^412^ ^]^ and neuroprosthetic implants.^[^ ^415^ ^]^ While passive electrodes have been widely employed, integrating transistors into neural recording devices offers advantages including local signal amplification for enhanced SNR, enabling clearer waveform resolution.^[^ ^416^ ^]^ OECTs, in particular, have emerged as promising platforms for implantable brain signal interfaces due to their low‐voltage operation, biocompatibility and mechanical conformability,^[^ ^417^ ^]^ supporting the recording of local field potential (LFP),^[^ ^392^, ^418^, ^419^, ^420^, ^421^ ^]^ single‐unit activity (SUA),^[^ ^420^, ^421^ ^]^ and electrocorticography (ECoG) signals.^[^ ^405^, ^422^, ^423^ ^]^ However, in addition to channel hydrophobicity, their switching speed remains limited by slow ionic transport,^[^ ^424^ ^]^ restricting their ability to capture fast neuronal events, such as single‐neuron activity (≈1 kHz).^[^ ^425^ ^]^ High‐speed operation is also crucial for wireless signal transmission to enable brain recording without bulky electrode wiring that limits mobility and increases noise.^[^ ^426^ ^]^ Cea et al. addressed the speed limitations of OECTs by adopting a vertical channel orientation while minimizing crosstalk by introducing a hydration access conduit (H‐via) in their vertically integrated ion‐gated transistor (vIGT) (Figure 14b).^[^ ^418^ ^]^ Two types of vertically oriented PEDOT:PSS channels were developed: a depletion‐mode channel incorporating D‐sorbitol and an enhancement‐mode channel modified with polyethylenimine (PEI). Chitosan served as the ionic membrane to mediate ion transport between the gate and channel. In the vertical configuration, the channel length is defined by the film thickness, allowing for ultra‐short channels that generate strong electric fields to accelerate ion drift, thereby enhancing response speed.^[^ ^427^ ^]^ Additionally, to prevent cross‐talk in ion‐rich environments, like cerebrospinal fluid, the device was encapsulated with parylene C, with a localized H‐via providing selective hydration of the active region, ensuring ionic conduction and EDL formation. These combined strategies achieved a temporal response of 900 ns, enabling megahertz‐range operation in a stand‐alone neural interface device. The vIGT, placed on the surface of the rat's skull, recorded somatosensory evoked potentials from hindlimb electrical stimulation. Operating as a common‐source amplifier, it amplified the physiological signals at the gate and transmitted the amplified wave through the ion‐rich medium via a third contact. The received signal is then differentially amplified relative to the original sine wave to extract the encoded physiological information, achieving SNRs comparable to conventional devices. These advancements demonstrate the feasibility of high‐speed, OECT‐integrated neural interfaces capable of capturing rapid neuronal activity with high fidelity.

While the vIGT demonstrated high‐speed and low‐noise operation through vertical architecture, Uguz et al. proposed a complementary strategy by integrating an organic electrochemical diode (OED) with an OECT to suppress leakage current, a key source of crosstalk in brain signal recording (Figure 14c).^[^ ^419^ ^]^ The vertical p–n junction OED was formed using n‐type p(C_6_NDI‐T) and p‐type PEDOT:PSS. To evaluate the rectifying behavior of the p–n OED, cations from the electrolyte were first injected into the top PEDOT:PSS layer. Under a positive forward bias, cations migrate into the n‐type p(C_6_NDI‐T), compensating injected electrons and increasing its conductance. Under reverse bias, cations move into the PEDOT:PSS layer and neutralize the sulfonate anions on PSS, and extracting holes at the anode, which reduces the hole density of PEDOT film. Simultaneously, p(C_6_NDI‐T) loses cations, resulting in high resistance between the electrodes and enabling unidirectional conductivity in the p–n OED. In the OECT, p(g2T‐TT) was used both as the channel and as a capping layer to seal the p–n OED. Due to its low water absorption and the caging of cations within its glycolated side chains, p(g2T‐TT) exhibits low cation mobility. In addition, SU‐8 photoresist layer was applied on top of p(g2T‐TT) for further encapsulation. Together, p(g2T‐TT) and SU‐8 form an ion barrier over the p‐n OED, confining cations within the diode and suppressing leakage current. For OED–OECT operation, a negative gate voltage is applied to OECT, injecting anions from the electrolyte into the channel and doping it into a conductive state. The device is switched on by applying a negative cathode‐source potential (V_CS_), where the p–n OED conducts and the OECT drain current flows through it as the cathode current (I_C_). Any modulation in OECT current from gate potential changes is thus reflected in I_C_, where biopotentials in the electrolyte can be effectively amplified and recorded. When a positive V_CS_ is applied, the rectifying behavior of the p–n OED blocks current flow in the OED–OECT device, switching it to the off‐state. This study presents a novel strategy for minimizing crosstalk in neural recordings by confining ionic flow and enabling unidirectional signal propagation. However, the multilayer architecture involving multiple materials introduces fabrication complexity.

OECT‐based inverters typically require separate p‐type and n‐type organic semiconductors or OMIECs, complicating fabrication. To simplify this device architecture, Cong et al. developed a diketopyrrolopyrrole (DPP)‐based ambipolar OMIEC, p(gDPP‐V), enabling single‐material inverter fabrication with both volatile and non‐volatile behavior (Figure 14d).^[^ ^407^ ^]^ Ambipolarity was achieved by aligning highest occupied molecular orbital (HOMO) and lowest occupied molecular orbital (LUMO) levels of p(gDPP‐V) with the electrode work function, allowing efficient injection and transport of both holes and electrons. The p(gDPP‐V)‐based OECT exhibited gate‐ and drain‐voltage‐dependent p‐type and n‐type characteristics with NaCl electrolyte. A vertically stacked ambipolar inverter showed voltage (V_DD_)‐dependent volatility due to polaron and bipolaron generation upon charging. At lower voltages (−0.5 V), reduction‐induced polaron accompanied Na^+^ ion penetration into p(gDPP‐V) to ensure charge neutrality. At 0.5 V, since oxidation produced few polarons, ion concentration is lower than reduction progress, resulting in non‐volatility. At higher voltages (0.8 V), ion concentrations of negative charge/anion and positive charge/cation are balanced, enabling volatile operation. This tunable behavior enables dual‐mode operation: non‐volatile for neuromorphic applications such as convolutional neural network, and volatile for electrophysiological recording. The OECT inverter successfully recorded EOG signals by amplifying cornea‐retina potential variations during horizontal eye movements. The voltage‐tunable, single‐material ambipolar inverter offers a scalable and energy‐efficient platform for future neuromorphic and biosensing technologies.

OECTs have shown strong promises for electrophysiological recording, owing to high g_m_ that enables on‐site signal amplification, leading to high sensitivity. Recent efforts have focused on accelerating response times and mitigating crosstalk, enabling more accurate and high‐resolution electrophysiological recording across diverse biological interfaces. Subsequent advancements are being directed toward enhancing mechanical properties such as flexibility^[^ ^408^ ^]^ and stretchability^[^ ^402^, ^428^ ^]^ to ensure compliance with body movements, as well as integrating with non‐volatile synaptic devices to enable signal processing^[^ ^429^ ^]^ and reservoir computing^[^ ^430^ ^]^ for real‐time diagnostics. Despite these advances, operational stability remains a significant challenge, particularly for n‐type OECTs.^[^ ^431^ ^]^ During electrophysiological recording, exposure to ambient air or aqueous environments can induce oxidative degradation in the organic semiconductor, compromising long‐term signal fidelity and impeding their practical implementation in continuous or implantable bioelectronic systems. Looking ahead, future developments should prioritize improving device stability to ensure long‐term, reliable operation in complex bioelectronic environments.

#### 4.2.2 OECTs for Biomarker Sensing

Beyond electrophysiological recording, OECTs offer a robust platform for biomarker sensing due to their ability to amplify subtle ionic and biochemical signals.^[^ ^30^, ^432^ ^]^ Their volumetric gating mechanism provides high sensitivity to subtle ionic concentration changes, while the ability to exchange ions between the active channel and electrolyte makes OECTs well‐suited for detecting a broad range of biological analytes, including neurotransmitters,^[^ ^433^, ^434^, ^435^ ^]^ metabolites,^[^ ^207^, ^344^, ^393^, ^436^, ^437^, ^438^, ^439^, ^440^ ^]^ deoxyribonucleic acid (DNA),^[^ ^441^, ^442^ ^]^ ribonucleic acid (RNA),^[^ ^345^, ^443^ ^]^ growth factors,^[^ ^346^, ^444^, ^445^ ^]^ and disease related biomarkers^[^ ^446^, ^447^, ^448^, ^449^, ^450^, ^451^ ^]^ (**Table** **9**). Furthermore, their compatibility with soft, aqueous environments enables direct integration with biological tissues and fluids, facilitating real‐time, label‐free sensing in both in vitro and in vivo settings.

**Table 9:** OECTs for biomarker sensing.

Biomarker type^a)^	Electrolyte layer^b)^	Channel^c)^	Sensing sites^d)^	Sensing mechanism^e)^	Other features	References
miRNA‐21	PBS containing L‐cysteine	PEDOT:PSS	Functionalized gate (DL‐PS‐MOF/TiO_2_ NR)	miRNA‐triggered DNSs growth at the gate	Light‐modulated g_m_	[345]
miRNA‐10b	PBS containing ascorbic acid	PEDOT:PSS	Functionalized gate (aldehyde group, walker‐blocker strands and hairpin strand1)	DNA walker and BCP reaction at the gate	Light‐accelerated Faradaic detection	[443]
HTLV‐DNA	PBS containing ascorbic acid	PEDOT:PSS	Functionalized gate (COF‐on‐MOF)	HTLV‐II DNA triggered GWS growth at the gate	Light‐modulated I_On_/I_Off_	[442]
Sweat (Na^+^, K^+^)	PBS in PDMS reservoir	PEDOT:PSS	Channel	Electrolyte permeation and interaction with the channel	3D‐IP, TP channel	[344]
Lactate, glucose	PBS	DI water + THF	Functionalized gate (LOx, GOx)	Enzymatic oxidation	Fiber	[438]
Glucose	PAAm‐Na^+^‐alginate hydrogel with GOx	PEDOT:PSS	Electrolyte (hydrogel)	Glucose oxidation by GOx followed by enzyme oxidation	Microneedle patch, wireless readout	[439]
	PBS	f‐BSTI2g‐SVSCN	Functionalized gate (GOx‐loaded PtNP/PAni hydrogel)	Enzymatic oxidation followed by redox byproduct catalysis at the gate	Low detection limit	[440]
	PBS	p‐PEDOT‐APD	Functionalized gate (GOx)		Nanoporous aerogel channel	[400]
	PBS	Hydro‐SC (p(g2T‐T) + PAAm)	Channel (GOx immobilization)	Enzymatic oxidation followed by channel oxidation by redox byproduct	Hydrogel semiconductor, stretchable, conformable	[207]
SARS‐CoV‐2 IgG	PBS	PEDOT:PSS	Functionalized gate (MAA)	Antigen–antibody binding	Side gate	[446]
SARS‐CoV‐2 spike protein	Saliva	p(C_6_NDI‐T)	Functionalized gate (nanobody‐SpyCatcher fusion proteins)	Protein–nanobody binding	Short detection time	[447]
	Saliva	p(g0T2‐g6T2)	Functionalized gate (SpyDirect nanobody)		Eco‐friendly	[448]
Bacterial virulence from wound exudate (S. aureus)	PBS	PEDOT:PSS	Functionalized gate (DNA hydrogel)	DNA hydrogel degradation by enzyme (DNase) from S. aureus	Flexible	[449]
AD biomarkers (Aβ_1‐40_, Aβ_1‐42_, t‐tau, and p‐tau^181^)	PBS with FBS	p(g2T‐T)	Functionalized gate (3‐MPA, 11‐MUA)	Antigen–antibody binding	Microelectrode array gate	[450]
EGFR	PBS in PDMS well	Gefitinib‐grafted PEDOT:PSS	Channel	Replacement interaction between probe (gefitinib) and the target	Reusable	[346]
TGF‐β_1_	PBS	PEDOT:PSS	Functionalized gate (MB‐modified TGF‐β_1_ aptamer)	Aptamer–target binding	Channel as the counter electrode	[445]
Dopamine	aCSF	PEDOT:PSS	Gate (carbon fiber microelectrode)	Redox reaction at the gate	In vivo monitoring	[434]
	PBS	P3MEET‐PTES	Gate (Ag/AgCl)		Flexible	[435]

^a)^ HTLV (human T‐cell lymphotropic virus type II), SARS‐CoV‐2: severe acute respiratory syndrome coronavirus 2, IgG: immunoglobulin G, AD: Alzheimer's disease, Aβ_1‐40_, Aβ_1‐42_: amyloid‐β, t‐tau: total tau, p‐tau^181^: phosphorylated tau, TGF‐β_1_: transforming growth factor beta 1

^b)^ FBS: fetal bovine serum, aCSF: artificial cerebrospinal fluid

^c)^ DI: deionized, THF: tetrahydrofuran, BTI: bithiophene imide, SVSCN: selenophene–vinylene–selenophene, p(g2T‐T): (2‐(3,3′‐bis(2‐(2‐(2‐methoxyethoxy) ethoxy) ethoxy)‐[2,2′‐bithiophen]‐5)yl thiophene, hydro‐SC: hydrogel semiconductor, P3MEET: poly(3‐(methoxyethoxyethoxy)thiophene), PTES: poly(4‐episulfide2,2,6,6‐tetramethylpiperidine‐1‐oxyl)

^d)^ MOF: metal‐organic framework, COF: covalent‐organic framework, LOx: lactate oxidase, PAni: polyaniline, MAA: mercaptoacetic acid, 3‐MPA: 3‐mercaptopropionic acid, 11‐MUA: 11‐mercaptoundecanoic acid, MB: methylene blue

^e)^ BCP: biocatalytic precipitation, GWS: G‐quadruplex wires superstructure

**Figure 15:**
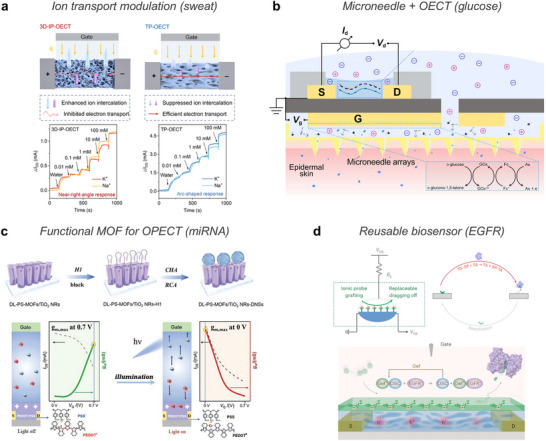
OECT‐based platforms for biomarker detection. a) Modulation of the OECT channel structure with 3D‐IP or TP architectures for differentiated ion transport, resulting in distinct current response profiles to sweat exposure. Reproduced with permission.^[^ ^344^ ^]^ Copyright 2025, Elsevier Inc. b) Integration of a microneedle patch, a soft and adhesive hydrogel buffer membrane, an OECT‐based glucose sensor, and a miniaturized OECT readout system for continuous glucose monitoring. Reproduced with permission.^[^ ^439^ ^]^ Copyright 2024, The American Association for the Advancement of Science under CC BY 4.0 license http://creativecommons.org/licenses/by/4.0/. c) An OECT incorporating photosensitive DL‐PS‐MOFs/TiO_2_ NRs for zero‐gate‐bias operation in miRNA detection. Reproduced with permission.^[^ ^345^ ^]^ Copyright 2023, Wiley‐VCH GmbH. d) An OECT‐based reusable biosensor utilizing ionic probe grafting with gefitinib and a replaceable, drag‐off binding mechanism for EGFR target detection. Reproduced with permission.^[^ ^346^ ^]^ Copyright 2024, Springer Nature.

Among various biofluids, sweat has gained attention as a non‐invasive, information‐rich medium for health monitoring. It contains biomarkers such as electrolytes,^[^ ^452^ ^]^ metabolites,^[^ ^453^ ^]^ nutrients,^[^ ^330^ ^]^ hormones,^[^ ^454^ ^]^ and proteins^[^ ^455^ ^]^ that reflect physiological states such as hydration,^[^ ^456^ ^]^ stress,^[^ ^457^, ^458^ ^]^ haemodynamic^[^ ^459^ ^]^ and metabolic function.^[^ ^460^ ^]^ Its individual‐specific composition also offers potential for personalized identification. Yang et al. demonstrated a sweat‐analysis OECT platform by structurally engineering PEDOT:PSS channels to modulate ion transport dynamics and create unique electrical fingerprints of sweat (**Figure** **15**a).^[^ ^344^ ^]^ The OECT sweat sensor features a 3D‐interconnected porous (3D‐IP) structure for efficient ion diffusion and a denser tabular porous (TP) structure for improved electronic conductivity via enhanced PEDOT connectivity. The 3D‐IP‐OECT exhibited fast, square‐like current responses across a wide Na⁺/K⁺ concentration range (0.01–100 mM), while the denser TP‐OECT showed slower, arc‐shaped responses due to limited ion permeability. To extract detailed sweat fingerprint information, an array of eight OECTs—four 3D‐IP and four TP devices—was fabricated with 2‐, 4‐, 6‐, and 8‐layer PEDOT:PSS channels. Thinner, porous 3D‐IP channels yielded fast, right‐angled response curves, while thicker, denser TP channels produced slower, arc‐shaped responses. These structural and thickness‐dependent variations in ion dynamics enable the array to distinguish between Na⁺ and K⁺ levels, forming a unique electrical fingerprint of sweat. A convolutional neural network (CNN) algorithm was then employed to classify artificial and real sweat samples from different individuals based on these ion dynamic signatures. This study presents a novel approach to leveraging ion dynamics for differentiating sweat composition, demonstrating potential for advanced biosensing applications. However, the use of syringe‐injected sweat, rather than real‐time, on‐skin collection, limits its physiological accuracy, highlighting the need for future integration with direct sampling platforms.

Glucose monitoring is central to diabetes management, requiring accurate, real‐time data to guide insulin therapy and lifestyle decisions.^[^ ^461^ ^]^ To address the limitations of traditional finger‐prick methods, microneedle‐based sensors have emerged as minimally invasive alternatives.^[^ ^462^, ^463^, ^464^, ^465^, ^466^ ^]^ These devices penetrate only the outer skin layer, avoiding pain receptors while accessing interstitial fluid (ISF), where glucose levels closely mirror those in blood.^[^ ^467^ ^]^ Due to small ISF volume, high sensitivity is crucial for reliable detection.^[^ ^468^ ^]^ In contrast, OECT‐based sensors offer superior sensitivity due to intrinsic signal amplification.^[^ ^438^ ^]^ Integrating OECTs with microneedle arrays presents a transformative solution, combining high sensitivity with minimally invasive access to ISF, thereby enabling stable and continuous glucose monitoring (CGM). Bai et al. developed a coin‐sized CGM system by integrating a hollow microneedle array patch, a soft adhesive enzyme‐loaded hydrogel membrane, an OECT‐based glucose sensor, and a miniaturized readout circuit (Figure 15b).^[^ ^439^ ^]^ The hydrogel comprises an interpenetrating network (IPN) of PAAm and Na^+^‐alginate, forming a double‐network (DN) structure embedded with glucose oxidase (GOx). The 3D‐printed gold‐coated microneedles ensure biocompatibility and durability. The OECT employed PEDOT:PSS as the active channel material. Upon skin penetration, glucose diffuses from ISF through the hydrogel, where it is oxidized by GOx. The reduced GOx is then regenerated by oxidizing aminoferrocene, which in turn becomes reduced. This reduced mediator (i.e., aminoferrocene) is subsequently oxidized at the gate electrode, generating a faradaic gate current (I_G_) and inducing a slight gate voltage shift (ΔV_G_). The OECT amplifies this ΔV_G_, producing a large modulation in I_DS_ and significantly enhancing the SNR. The system demonstrated linear detection across a broad glucose concentration range (10^−6^ to 10^−1^ M), high selectivity against interferent, and a high SNR (60 dB). This work highlights the potential of integrating microneedles with OECTs, offering a compelling strategy for continuous biomarker monitoring that combines the minimal invasiveness and sampling efficiency of conventional sensors with the high sensitivity and amplification capability of OECTs.

For continuous biomarker monitoring, minimizing power consumption is essential, particularly in wearable systems that demand sustained operation. OECTs typically operate near their maximum g_m_, which often requires application of V_G_. Since power consumption scales with applied voltage, reducing the V_G_ required to reach maximum g_m_ directly contributes to lowering the overall power demand of the system. Gao et al. reported an organic photoelectrochemical transistor (OPECT) for microRNA (miRNA) detection that enables high maximum g_m_ operation at zero bias (Figure 15c).^[^ ^345^ ^]^ The device uses a photosensitive dual‐ligand metal–organic frameworks (DL‐PS‐MOFs), composed of [5,10,15,20‐(4‐carboxyphenyl) porphyrin]Zn(II) (ZnTCPP) and 2‐aminoterephthalic acid (ATA), grown on TiO_2_ nanorods (NRs) as a gate electrode, PEDOT:PSS as a channel, and PBS containing L‐cysteine as an electrolyte. Ultrasensitive detection is achieved via a stepwise growth process of DNA nanospheres (DNSs) on the DL‐PS‐MOF/TiO_2_ NR surface. The gating mechanism relies on interfacial potential modulation from negatively charged DNSs and light‐induced photovoltage (V_p_). In the dark, DNS‐induced negative surface charges increase the gate offset voltage (V_offset_) via the surface dipole effect, shifting the transfer curve. Upon 425 nm light illumination, a V_p_ is generated, increasing the effective gate voltage ($V_{G}^{\mathrm{eff}}$) and shifting the transfer curve to lower V_G_, achieving maximum g_m_ at zero V_G_. As more DNSs accumulate on the gate, the increasing charge density gradually suppresses the V_p_‐induced shift, resulting in smaller $V_{G}^{\mathrm{eff}}$ shifts and distinct ΔI_DS_ values, defined as the difference in drain current between dark and illuminated states. These changes allow miRNA‐21 detection by tracking ΔI_DS_. This work demonstrates the potential of leveraging light–matter interaction to modulate OECT channel conductance, enabling bias‐free, high‐sensitivity biosensing and paving the way for low‐power, programmable sensing platforms. However, its current limitation lies in the specificity to miRNA‐triggered DNA amplification and the non‐reusability arising from the irreversible formation of DNA nanostructures on the gate surface.

Reusability is a critical concern for chemical sensors, as many conventional designs suffer from limited operational lifespan due to irreversible analyte binding, surface fouling,^[^ ^469^ ^]^ or the need for regeneration steps involving harsh chemicals or complex procedures.^[^ ^470^ ^]^ For continuous monitoring or cost‐sensitive applications, sensors must retain sensitivity and selectivity over multiple cycles while enabling rapid interface restoration without compromising performance. Jiang et al. developed a reusable cancer biomarker sensor targeting epidermal growth factor receptor (EGFR) using a drug‐mediated‐OECT (DM‐OECT), in which gefitinib serves as an ionic probe (Figure 15d).^[^ ^346^ ^]^ The DM‐OECT is fabricated by immersing a PEDOT:PSS‐based OECT in a gefitinib solution, allowing easy integration of the probe into the transistor interface. The device leverages the electrostatic interaction between protonated gefitinib and PEDOT:PSS channel, along with the strong binding of gefitinib to EGFR, to achieve a refresh‐in‐sensing (RIS) capability. This allows the device to simultaneously detect the target and self‐refresh without additional reagents or steps. Unlike conventional sensing processes that rely on sequential probe–analyte binding and subsequent dissociation for reactivation, the RIS strategy operates through a simplified competitive interaction between the sensor probe (gefitinib) and the target (EGFR). The RIS function in the DM‐OECT is driven by dynamic interplay among the PEDOT:PSS layer, gefitinib, and EGFR, involving: i) alteration of the PEDOT:PSS interface by gefitinib grafting, ii) competitive replacement of gefitinib from PEDOT:PSS by high‐affinity binding to EGFR, and iii) resulting modulation of device conductivity due to changes in surface potential. The RIS‐enabled DM‐OECT achieved an exceptionally low limit of detection (LOD) of 5.74×10^−15^ g ml^−1^ and maintained performance over 200 regeneration cycles. It was successfully applied for the diagnosis of non‐small cell lung cancer. This study introduces an effective approach to achieving reusability in biosensing by allowing rapid and reliable regeneration of the sensing interface while maintaining high sensitivity and functional integrity.

Biomarker sensing using OECTs has made notable progress in minimizing power consumption, making them increasingly suitable for wearable and implantable diagnostic platforms. In parallel, strategies enabling sensor reusability, such as reversible probe binding, have enhanced cost‐efficiency and operational longevity. Despite these advances, most current OECT‐based biosensors are largely limited to single‐analyte detection, which constrains their utility in complex biological environments that demand simultaneous monitoring of multiple biomarkers for accurate diagnosis and personalized treatment. To overcome these limitations, strategies such as multi‐gate architectures^[^ ^445^, ^450^ ^]^ and multi‐sensor arrays^[^ ^471^ ^]^ have been proposed to enable selective, multi‐target detection. Meanwhile, most OECTs remain restricted to benchtop configurations, limiting their translational potential. For integration into real‐world wearable or point‐of‐care systems, devices that are mechanically flexible,^[^ ^472^ ^]^ portable,^[^ ^446^ ^]^ and capable of autonomous operation are highly desirable. Integrating self‐powered systems, such as solar cell‐based energy harvesting,^[^ ^473^ ^]^ would further enhance the practicality of OECT biosensors in mobile and resource‐limited environments.

## 5 Therapeutic Systems

Chronic wounds require continuous treatment and monitoring, yet conventional dressings like gauze and topical drugs offer only temporary protection and limited therapeutic effect, resulting in slow and passive healing without real‐time feedback.^[^ ^474^ ^]^ To improve outcomes, moisture‐retentive dressings that enable both accelerated healing and continuous monitoring are highly desirable. Ions play key roles in these systems due to their conductivity, water retention, antimicrobial activity, cell stimulation, and therapeutic potential supporting efficient healing.

**Figure 16:**
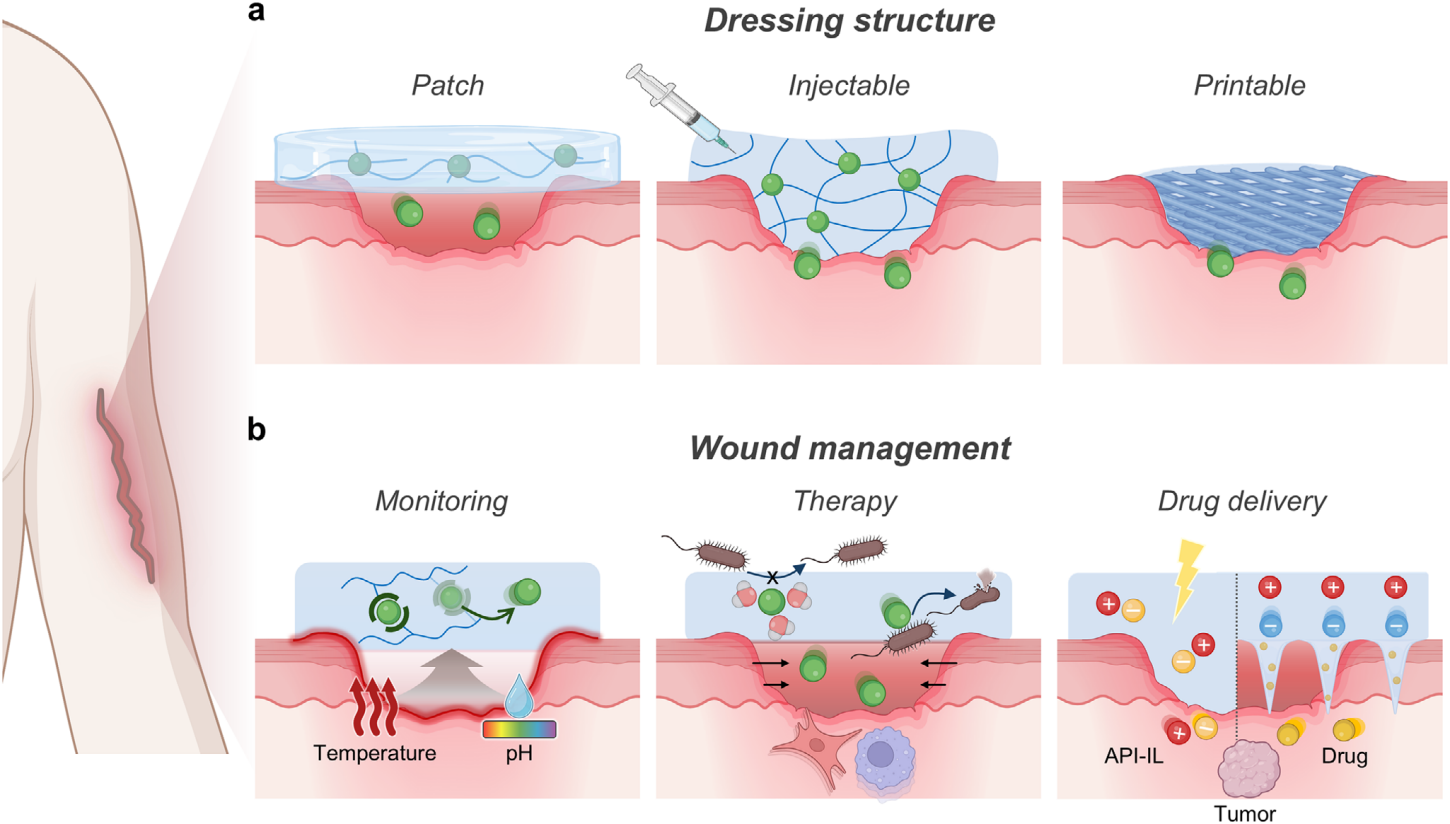
Ionic therapeutic systems. a) Schematic illustration of various ion‐based wound dressing designs, including patch‐type, injectable, and printable configurations, in which structural integrity is maintained through ionic crosslinking and metal–ligand coordination. b) Schematic illustration of ion‐mediated wound care systems for wound monitoring, wound therapy, and drug delivery. Part of the schematics were created using BioRender.com.

Ionic wound dressings adhere tightly to wound surface and deeper tissues through the use of ionic materials, serving as a protective barrier against external contaminants while providing an active healing environment, which includes patch‐type, injectable, and printable hydrogel dressings (**Figure** **16**a). Patch‐type dressings are directly applied to the wound, where ionic crosslinking and coordination within the polymer matrix enhance their mechanical stability and ensure robust attachment.^[^ ^475^ ^]^ Injectables conform to irregular wound geometries and enable minimally invasive treatment, where ionic crosslinking improves mechanical strength and allows manual injection.^[^ ^476^ ^]^ Printables allow precise construction of patient‐specific architectures that conform to complex wound shapes.^[^ ^477^ ^]^ Ionic wound dressings also provide integrated therapeutic functions, including real‐time monitoring, active therapy, and controlled drug delivery (Figure 16b). Coordinated ions within the gel dynamically respond to infection‐related signals like temperature and pH shifts, modulating ionic conductivity for wound status monitoring.^[^ ^79^, ^96^ ^]^ These ions also confer antifouling, antibacterial effects,^[^ ^478^ ^]^ enhance cell activation, and maintain hydration,^[^ ^479^ ^]^ all of which contribute to accelerate healing. For targeted treatment, transdermal drug delivery is achieved through ion penetration^[^ ^480^ ^]^ and electrostimulation‐driven migration of charged drugs.^[^ ^481^ ^]^ Overall, ionic wound dressings offer a versatile platform that combines structural integrity as well as wound treatment with responsive wound monitoring, active therapy, and precise drug delivery, offering strategies for advanced wound management.

### 5.1 Wound Monitoring

Skin serves as a primary protective barrier against external threats.^[^ ^482^ ^]^ While mild injuries can heal through a natural sequence, comprising hemostasis, inflammation, proliferation, and remodeling,^[^ ^483^ ^]^ chronic wounds, such as those caused by thermal burns^[^ ^484^ ^]^ or diabetes,^[^ ^485^ ^]^ often disrupt this process, leading to prolonged inflammation and delayed healing.^[^ ^486^ ^]^ To investigate chronic wound progression, smart wound monitoring systems have been developed for real‐time tracking of wound conditions. Upon injury, physiological biomarkers such as exudate secretion,^[^ ^487^ ^]^ pH,^[^ ^488^, ^489^ ^]^ and temperature^[^ ^475^ ^]^ dynamically change due to the immune responses and bacterial infections.^[^ ^475^, ^488^ ^]^ For instance, infection triggers immune cell infiltration and cytokine release,^[^ ^490^, ^491^ ^]^ raising local temperature.^[^ ^492^ ^]^ Simultaneously, wound pH shifts from its slightly acidic baseline (pH = 4–6, health skin) toward neutral (pH = 7–8), due to skin barrier breakdown bacterial infection.^[^ ^493^, ^494^ ^]^ Monitoring these biomarkers provides valuable insight into wound healing status and early signs of infection,^[^ ^495^ ^]^ enabling proper clinical interventions.^[^ ^496^ ^]^

**Figure 17:**
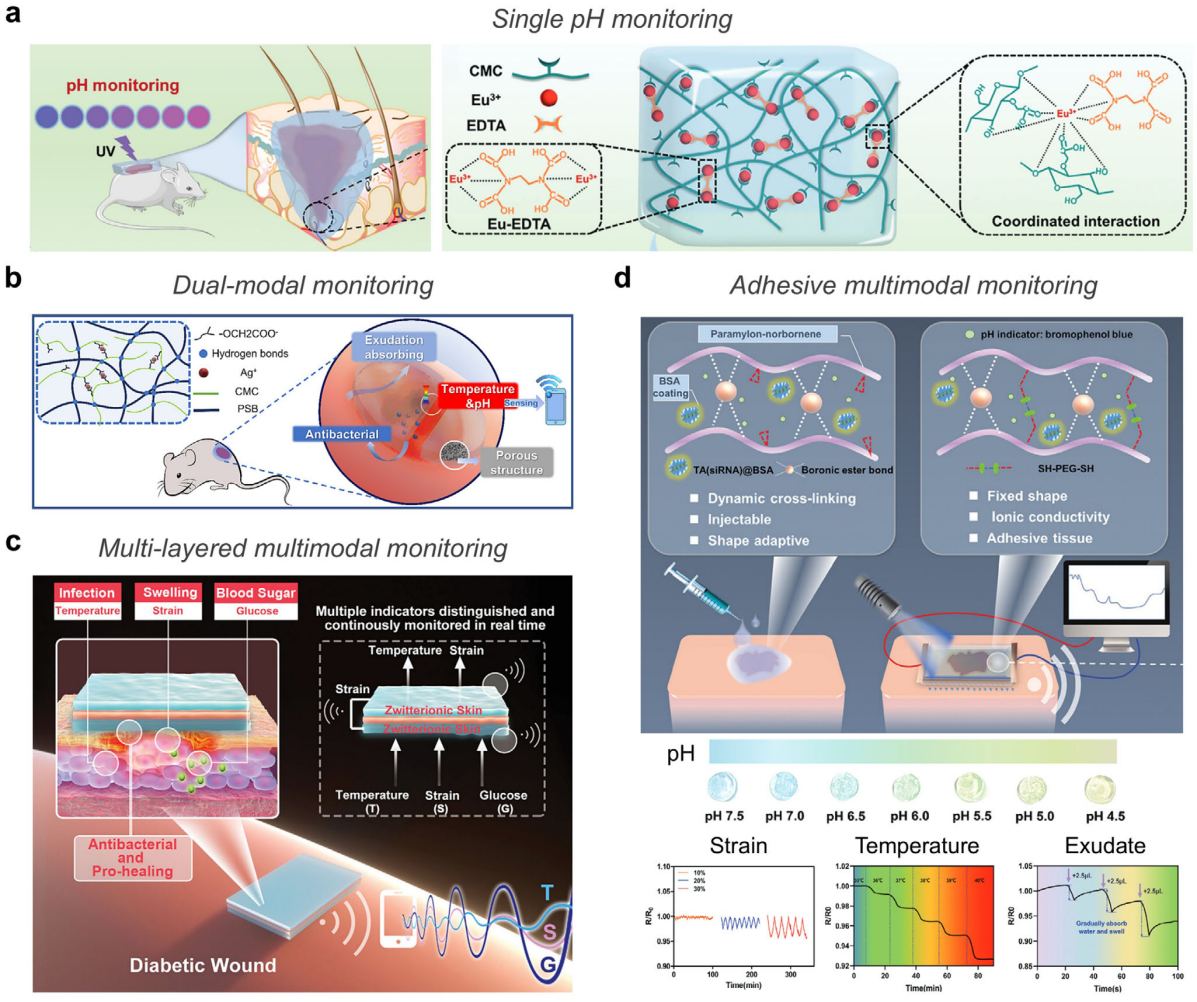
Wound monitoring systems. a) Eu^3+^‐mediated CMC‐EDTA hydrogel detecting pH levels with fluorescence under light stimulation. Reproduced with permission.^[^ ^96^ ^]^ Copyright 2024, Wiley‐VCH GmbH. b) Ag^+^‐coordinated zwitterionic PSB hydrogel in PSB/CMC‐Ag^+^ for wound pH and temperature monitoring. Reproduced with permission.^[^ ^79^ ^]^ Copyright 2022, Elsevier. c) Multi‐layered zwitterionic hydrogel sensor detecting temperature, glucose concentration, and strain signals. Reproduced with permission.^[^ ^136^ ^]^ Copyright 2021, Wiley‐VCH GmbH. d) Nanocomposite hydrogel for real‐time monitoring of pH, strain, temperature, and exudate. Reproduced with permission.^[^ ^475^ ^]^ Copyright 2024, Wiley‐VCH GmbH under CC BY 4.0 license http://creativecommons.org/licenses/by/4.0/.

Smart wound dressings capable of real‐time monitoring of wound‐related biomarkers allow continuous assessment of wound status, supporting accurate assessment of healing progression and infection status. This capability reduces unnecessary dressing replacements and enhances prognosis prediction. Ionically functionalized smart wound dressings improve biomarker sensitivity^[^ ^497^ ^]^ and support multiplexed sensing by regulating ionic conductivity in response to various wound biomarkers.^[^ ^136^ ^]^ Among wound biomarkers, pH serves as a key indicator of wound infection. While conventional electrical pH sensing requires external power and signal transmission modules,^[^ ^498^ ^]^ colorimetric sensing allows visual detection without auxiliary devices.^[^ ^499^ ^]^ To enable such responsiveness, ion–polymer coordination can transduce local ionic changes,^[^ ^500^ ^]^ triggering color transitions for wound monitoring. For example, Xie at al. developed an injectable hydrogel dressing incorporating europium‐ethylenediaminetetraacetic acid (Eu‐EDTA) complex within a carboxymethyl cellulose (CMC) matrix for pH‐responsive fluorescence (**Figure** **17**a).^[^ ^96^ ^]^ Eu^3+^ ions, acting as light‐harvesting antennae, undergo changes in coordination with protonation shifts under varying pH. This process modulates the red fluorescence emission at 593 nm (^5^D_0_→^7^F_1_ transition) and 616 nm (^5^D_0_→^7^F_2_ transition), with the fluorescence intensity ratio I(^5^D_0_→^7^F_2_)/I(^5^D_0_→^7^F_1_) showing a linear correlation with pH of wound environments (pH = 4.5–7.5), enabling quantitative visualization of wound pH changes. Although this pH‐indicating system shows effective color‐based wound sensing via ionic coordination, reliance on UV excitation and single‐biomarker detection limits clinical applicability, necessitating multiplexed platforms for complex wound environments.

Ionic‐mediated monitoring systems are increasing used for multimodal monitoring of wound biomarkers, due to their high responsiveness via ionic conductivity changes. For example, Long et al. developed a zwitterionic CMC‐based hydrogel sensor, monitoring both pH and temperature changes in wound (Figure 17b).^[^ ^79^ ^]^ The hydrogel network is crosslinked through Ag^+^–carboxyl coordination, while the zwitterionic poly(sulfobetaine methacrylate) (PSB) reduces bacterial adhesion. The sensing mechanism relies on Ag^+^ conductivity modulation. For pH sensing, H^+^ ions from wound displace Ag^+^ from coordination sites, increasing free Ag^+^ content and thereby lowering the hydrogel resistance as pH decreases. For temperature sensing, elevated temperature enhances Ag^+^ mobility, reducing resistance. While this system enables dual biomarker detection, both stimuli modulate ionic conductivity, making it challenging to distinguish between signals and thereby limiting independent analysis.

Beyond pH and temperature, glucose levels and mechanical strain^[^ ^501^, ^502^ ^]^ are also important biomarkers for wound monitoring. Elevated glucose in wound fluid, common in diabetic wounds, impairs leukocyte function, increasing susceptibility to infection.^[^ ^503^ ^]^ Simultaneously, mechanical strain at the wound site can indicate local hyperemia and oedema, providing insight into immune responses and local vascular state.^[^ ^136^ ^]^ Therefore, multi‐stimuli sensing of biochemical and biomechanical biomarkers offer an accurate wound assessment,^[^ ^504^ ^]^ but simultaneous detection often faces signal interference, making stimulus decoupling a major challenge. To address this issue, Guo et al. developed a multi‐layered zwitterionic hydrogel sensor capable of decoupling three wound indicators—temperature, glucose concentration, and strain (Figure 17c).^[^ ^136^ ^]^ The sensor consists of two zwitterionic hydrogels, each layer composed of zwitterion (SBMA) for antifouling, N‐isopropyl acrylamide (NIPAAm) for temperature responsiveness, and methylacrylamide phenylboric acid (MPBA) for glucose responsiveness, with NaCl providing ionic conductivity. These layers sandwich an insulating VHB layer, forming two resistance sensors and one capacitance sensor in a single device. Each stimulus is transduced as follows: (i) temperature increase induces hydrophobic transition in NIPAAm, releasing ions and decreasing resistance, (ii) glucose binds to MPBA, dehydrating the hydrogel, increasing ion concentration and lowering resistance, and (iii) compressive strain reduces the distance between layers, increasing capacitance. By analyzing three resistance outputs: R_0_ (strain dependent), R_1_ from the lower layer contacted with wound (temperature, glucose, and strain dependent), and R_2_ from the upper layer (temperature and strain dependent), the system calculates differences |ΔR_0_|, |Δ(R_2_−R_0_)|, and |Δ(R_1_−R_2_)|, to isolate each signal with minimal crosstalk. Despite its sensing advantages, the thick, multi‐layered architecture (2.5 mm thickness), and the need for an adhesive layer may hinder its applicability as a practical wound dressing.

For user‐friendly, multimodal wound monitoring, a single‐layer ionic hydrogel with inherent adhesion is highly desirable. Lei et al. developed a multifunctional nanocomposite hydrogel designed for real‐time wound monitoring and treatment, with emphasis on mechanical comfort and adhesion (Figure 17d).^[^ ^475^ ^]^ The hydrogel comprises mercapto polyethylene glycol (SH‐PEG‐SH) crosslinked via borate bonds using borax and further crosslinked under light with norbornene dianhydride–modified paramylon (N–P), which enhances hemostasis and tissue adhesion. This system also incorporates tannins and small interfering RNA (siRNA) coated with bovine serum albumin (BSA) as biocompatible therapeutic agents, and bromothymol blue as a colorimetric pH indicator. The hydrogel design enables multiple sensing modalities: i) a decrease in pH due to infection induces a visible color shift in the bromothymol blue indicator from slate‐blue to yellowish green; ii) mechanical strain alters the borate bonding density, modulating ionic pathways and resistance;^[^ ^505^ ^]^ iii) temperature elevations partially rupture borate bonds, softening the network and reducing resistance; and iv) exudate influx causes hydrogel swelling, leading to a shift in resistance. Although all signals modulate resistance, they produce distinct response profiles (shape, response time), distinguish each stimulus. This integrated, adhesive, and flexible wound dressing combines colorimetric pH sensing with resistive detection of strain, temperature, and exudate, offering comfort and real‐time diagnostic potential.

Ionic systems integrated into wound dressings facilitate multi‐sensing of wound biomarkers, such as pH, temperature, strain, and exudate, through changes in ionic conductivity (**Table** **10**). However, as the ionic conductivity is simultaneously influenced by multiple parameters, complete signal decoupling remains challenging. Unlike conventional multimodal systems that use separate non‐ionic sensors,^[^ ^506^, ^507^, ^508^ ^]^ ionic platforms remain limited in the diversity of biomarker detection. Future designs should achieve selective and decoupled detection to broaden diagnostic capabilities beyond basic physicochemical cues.^[^ ^509^ ^]^

**Table 10:** Wound monitoring systems.

Sensing target	Ionic material^a)^	Polymer matrix^b)^	Functions of ions	Sensing mechanism	Therapeutic Properties	Applications	References
pH	Cu_2_O (Cu^n+^)	PVA/PANI	Ionic interaction (doping)	pH‐induced hydrogen or hydronium ions binding	‐	Contactless wound monitoring bandage	[489]
	Eu‐EDTA	CMC	Ionic coordination dynamics	Altering Eu^3+^‐coordination	Promoting angiogenesis, self‐healing property	Diabetic wound monitoring	[96]
Strain	LiCl	PVA/PEI/PAM/GelMA	Ionic coordination	Altering interplanar spacing distance of coordination	Antibacterial property, drug delivery	Diabetic wound monitoring and healing	[501]
	QCS/AAc/AMPS	PAAm	Electrostatic interaction for sol‐gel transition	Piezoelectric effect by migration of charges	Antibacterial property, hemostasis	Wound monitoring and healing	[502]
Multimodal	Ag^+^/CMC/PSB	CMC/PSB	Ionic coordination dynamics	pH: altering Ag^+^‐coordination Temperature: altering ionic conductivity	Antibacterial property	Diabetic wound monitoring	[79]
	Borax	N–P/LAP/SH‐PEG‐SH	Ionic interaction with polymer matrix	Strain: altering borate ionic bonding Temperature: altering borate coordination Exudate secretion: swelling induced ion concentration change	Antibacterial property, bioadhesion		[475]
	NaCl/SBMA	NPAAm/MPBA	Electrostatic interaction	Temperature: hydrophobic transition of NiPAAm and ion release Glucose: dehydration of hydrogel and ion concentration change Strain: altering migration of ions	Antibacterial property, pro‐healing	Diabetic wound monitoring and healing	[136]
	SBMA/AMPS/borax	PVA/THMA		Strain: directional alignment of ionic groups pH: altering electrostatic force difference in sulfonic acid groups Temperature: altering ionic conductivity	Antibacterial property, bioadhesion, hemostasis		[504]
	CaNO_3_	Starch/PAM	Ionic crosslinking	Temperature: temperature‐induced ionic conductivity change Exudate secretion: change in ionic concentration	Bioadhesion	Wound monitoring and healing, external robotic control	[487]

^a)^ AMPS: 2‐acrylamide‐2‐methylpro‐panesulfonic acid, QCS: quaternized chitosan, AAc: acrylic acid ^b)^ LAP: lithium phenyl‐2,4,6‐trimethylbenzoylphosphite, THMA: N‐[tris (hydroxymethyl) methyl] acrylamide

### 5.2 Wound Therapy

Skin injury induces pain and compromises the protective functions against external threats.^[^ ^474^ ^]^ Chronic wounds pose clinical and economic challenges due to prolonged treatment and frequent complications.^[^ ^510^ ^]^ Effective wound management is therefore essential to prevent microbial infection,^[^ ^511^ ^]^ modulate inflammation, and prevent tissue degradation. However, conventional approaches, such as gauze dressings and single‐use drug applications, offer only passive protection, often requiring frequent replacement and resulting in delayed healing and extended hospitalization.^[^ ^499^ ^]^ To accelerate wound healing, advanced wound dressings integrated with therapeutic properties have been developed, encompassing moisture retention,^[^ ^512^ ^]^ antibacterial activity,^[^ ^513^ ^]^ electrostimulation,^[^ ^514^, ^515^ ^]^ and immune regulation.^[^ ^516^, ^517^, ^518^ ^]^ While many of these systems rely on non‐ionic mechanisms, ionically conductive and zwitterionic hydrogels are particularly attractive for their biocompatibility and moisture retention,^[^ ^519^, ^520^ ^]^ and offer enhanced therapeutic effects—including antibacterial activity,^[^ ^521^, ^522^, ^523^ ^]^ hemostasis regulation,^[^ ^524^, ^525^ ^]^ therapeutic gas generation,^[^ ^526^, ^527^ ^]^ electrostimulation,^[^ ^528^, ^529^, ^530^ ^]^ and tissue regeneration^[^ ^531^ ^]^—providing a promising strategy for dynamic wound control and accelerated healing (**Table** **11**).

**Table 11:** Wound therapy systems.

Therapy mechanism	Ionic material^a)^	Polymer matrix^b)^	Functions of ions	Wound therapy	Additional properties	Wound healing applications	References
Antibacterial property	Borax	PVA/TA/HLC	Ionic crosslinking	Antibacterial property, hemostasis	Bioadhesion	Hemostasis of liver	[519]
	[VBIM][Br]	PAM/CNF	Chemical crosslinking, enhancing ion conductivity	Antibacterial property, hemostasis		Hemostasis of liver, rat skin	[521]
	Cu‐Zn doped BGns	PADM	Proinflammatory promotion, anti‐inflammatory	Antibacterial property, immunomodulation		Rat skin with excisional infection	[522]
	Ag^+^/Al^3+^	Lig/PAA	Ionic coordination, antibacterial property	Antibacterial property, cell migration		Rat wound healing	[478]
Hemostasis regulation	Ca^2+^	GelMA/PLD	Hemostasis regulation	Hemostasis, water retention		Rat, porcine skin	[525]
	Mg^2+^/Li^+^	MeTro/SNs	Ionic crosslinking	Hemostasis		Rat dorsal skin, porcine lung	[524]
Therapeutic gas generation	[HEIm][Br]/CO_3_^2−^	NAGA	Crosslinking with Chlorella	Light‐induced O_2_ generation	Antibacterial	Bacterial‐infected diabetic rat	[526]
	PIL	HAMA	Ionic flocculation	Chlorella‐based H_2_ production for fibroblast proliferation	Antibacterial, antioxidant	Diabetic rat	[527]
Electrostimulation	LiCl	PAM/ethylene glycol	Enhancing ionic conductivity	TENG‐induced electrical stimulation	‐	Diabetic rat	[529]
	Ag^+^	Dopamine‐modified HAMA	Antibacterial property	Photovoltaic electrical stimulation	Antibacterial, inflammation regulation	Bacterial‐infected diabetic rat	[528]
	ChCl	PAA/glycerol/HA‐SH	Enhancing ionic conductivity	Electric stimulation‐induced cell migration	Angiogenesis, collagen deposition	Burn rat	[252]
	LiBr	CNTs‐TPU/PAM and CMCS hydrogel	Ionic conductivity, water retention	Self‐powered electrostimulation	Antibacterial, angiogenesis	Diabetic rat	[530]
Tissue regeneration	Fe^3+^	HA‐Cys‐Py	Ionic coordination	Electrical stimulation	Self‐healing	Rat sciatic nerve	[516]
	Cu^2+^	TEOS	Ionic coordination	Immunomodulation, coagulation	Endocytosis	Rat mandibula	[531]

^a)^ BGns: bioactive glass nanoparticles, [HEIm][Br]: 3‐(2‐hydroxyethyl)−1‐vinylimidazolium bromide, PIL: poly(3‐(2‐hydroxyethyl)‐1‐vinylimidazolium bromide)

^b)^ TA: tanic acid, HLC: human‐like collagen, CNF: cellulose nanofibers, PADM: porcine dermal extracellular matrix, Lig: lignin, PLD: poly(lactide‐co–propylene glycol–co‐lactide) dimethacrylate, MeTro: methacryloyl‐modified tropoelastin, SNs: silicate nanoplatelets, NAGA: N‐(2‐amino‐2‐oxoethyl)−2‐propenamide, HAMA: hyaluronic acid methacryloyl, HA‐SH: thiolated hyaluronic acid, HA‐Cys‐Py: pyrrole‐ and cysteamine dihydrochloride‐grafted hyaluronic acid, TEOS: tetraethyl orthosilicate

**Figure 18:**
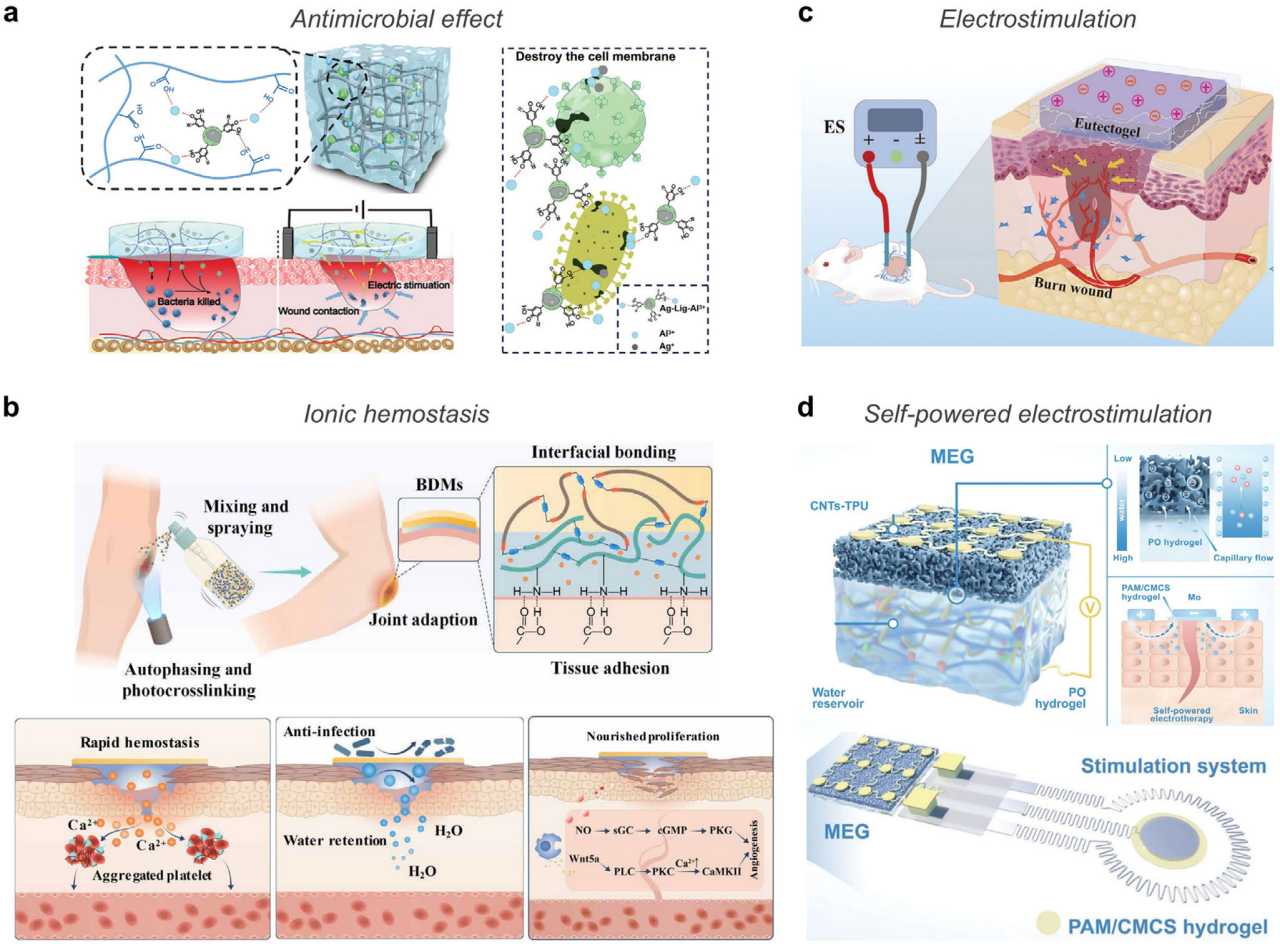
Wound therapy. a) Bimetallic phenolic network‐based antimicrobial hydrogel. Reproduced with permission.^[^ ^478^ ^]^ Copyright 2025, Wiley‐VCH GmbH. b) Sprayable biomimetic double‐layered hydrogel for ionic hemostasis. Reproduced with permission.^[^ ^525^ ^]^ Copyright 2024, The American Association for the Advancement of Science under CC BY 4.0. http://creativecommons.org/licenses/by/4.0/. c) Conductive eutectogel‐based ES therapy. Reproduced with permission.^[^ ^252^ ^]^ Copyright 2024, Wiley‐VCH GmbH. d) MEG‐based wound dressing for self‐powered ES therapy. Reproduced with permission.^[^ ^530^ ^]^ Copyright 2025, Wiley‐VCH GmbH.

Incorporating antimicrobial functionality into high‐moisture hydrogels significantly enhances their therapeutic efficacy by preventing infection‐induced delays in wound healing. Among various strategies, the integration of antimicrobial metal ions such as Ag^+^,^[^ ^478^ ^]^ Cu^2+^,^[^ ^532^ ^]^ and Zn^2+[^ ^533^ ^]^ into hydrogels has proven effective. These ions electrostatically interact with bacteria and destroy bacterial cell membranes.^[^ ^478^, ^533^ ^]^ Yang et al. reported a lignin (Lig) and polyacrylic acid (PAA)‐based hydrogel incorporating both Ag^+^ and Al^3+^ ions to form a bimetallic phenolic network hydrogel of PAA/Ag‐Lig‐Al^3+^ (PALA) for wound care (**Figure** **18**a).^[^ ^478^ ^]^ The system exhibits strong antimicrobial activity, robust mechanical strength, and excellent tissue adhesion due to ion‐mediated coordination. Specifically, Al^3+^ accelerates PAA polymerization and enhance mechanical resilience via energy‐dissipating coordination, while Al^3+^/Al^2+^ oxidizes catechol in Lig to release antimicrobial phenolic hydroxyl groups. Ag^+^ disrupts bacterial membranes by binding sulfur‐containing proteins, and its synergy with Al^3+^ amplifies antimicrobial effects by altering cell permeability. The PALA hydrogel also exhibits high conductivity for electrotherapy, where electrostimulation promotes Ag^+^ release, consequent cell migration, and antibacterial performance. However, the excessive use of bioactive metal ions raises concerns regarding cytotoxicity toward healthy tissue cells^[^ ^534^, ^535^ ^]^ and the emergence of antimicrobial resistance.^[^ ^536^ ^]^ Therefore, while ion incorporation offers therapeutic advantages, careful dosage control and biocompatibility assessment are essential for safe clinical treatment.

Sprayable hydrogels are particularly attractive in clinical applications due to their superior flexibility^[^ ^537^ ^]^ and adaptability to irregularly shaped wounds.^[^ ^479^ ^]^ However, conventional sprayable hydrogels often face limitations in sustaining adequate hydration due to water evaporation and rapid degradation.^[^ ^499^, ^538^ ^]^ To address these limitations, Yang et al. developed a sprayable, biomimetic bilayer hydrogel designed for simultaneous moisture retention, antimicrobial action, and hemostasis (Figure 18b).^[^ ^525^ ^]^ The system features a hydrophobic top layer composed of poly(lactide‐co–propylene glycol–co‐lactide) dimethacrylate (PLD) integrated with triclosan (TCS), which serves to maintain hydration and offers antimicrobial activity. The hydrophilic GelMA‐Ca^2+^ bottom layer promote rapid hemostasis by releasing Ca^2+^ to activating clotting factors. The bilayer structure is achieved by coagulant CaCl_2_, where Ca^2+^ reduces droplet surface tension in GelMA–PLD emulsion, promoting phase separation. Subsequent UV‐induced photocrosslinking stabilizes the hydrogel network via covalent bonding between the methacryloyl groups of GelMA and the amine groups of tissue, resulting in strong adhesion. In a porcine wound model, the sprayable hydrogel reduced wound area to ≈83.6% after 7 days, versus 95.1% in controls. Therefore, this ion‐mediated hemostasis provides a biocompatible strategy for wound management, minimizing cytotoxicity while enhancing cellular activity. However, reliance of UV curing, potential need for additional adhesives to improve mechanical stability, and sensitivity to polymer density‐driving phase separation, remain challenges requiring further optimization for practical use.

Electrostimulation has gained increasing attention in wound therapy for its ability to promote VEGF expression and guide the directional migration of electrosensitive cells such as endothelial cells, macrophages, and fibroblasts.^[^ ^252^, ^529^, ^539^ ^]^ This approach typically relies on direct electrode‐wound contact, where poor interface conformity and high impedance can hinder therapeutic efficiency. To address this issue, ionically conductive hydrogels have been employed as conformal interface materials capable of mediating efficient electric field delivery to the wound.^[^ ^529^ ^]^ In this context, Tian et al. developed a conductive eutectogel wound dressing composed of polymerizable DESs (PDESs), including acrylamide, glycerol, and ChCl, which form a double network with thiolated hyaluronic acid (HA‐SH) (Figure 18c).^[^ ^252^ ^]^ The HA‐SH/PDES eutectogel (HPD gel) exhibits high ionic conductivity, supported by a porous structure that facilitate ChCl transport, while ChCl disrupts bacterial membranes through electrostatic interactions. The HPD gel forms strong hydrogen bonding with wound surface, reducing interfacial resistance and enhancing the efficiency of electrostimulation. In in a murine burn wound model, treatment with ChCl‐loaded HPD gel under electrostimulation significantly accelerated healing, achieving 80.91% wound closure by day 14, outperforming both the electrostimulation‐only group (65.32%) and the gel–only group (70.59%). These results demonstrate the HPD eutectogel with stable conductivity and wound compatibility allows for suitable application in electrotherapy. However, its reliance on external power underscores the need for self‐powered systems.

For self‐powered electrostimulation, triboelectric nanogenerators (TENGs) have been employed to drive ion transport without external voltage.^[^ ^529^ ^]^ While this approach eliminates the need for a power supply, it relies on external mechanical force, leading to unstable output. A promising solution involves moist‐electric generators (MEGs), which utilize water flow to produce a more consistent electrical output.^[^ ^18^ ^]^ Yan et al. developed an autonomous, moisture‐driven flexible electrogenerative dressing (AMFED) that integrates a MEG, molybdenum (Mo) electrodes, and an antibacterial hydrogel layer (Figure 18d).^[^ ^530^ ^]^ The MEG adopts a bilayer structure, comprising a carbon nanotube (CNT)‐modified thermoplastic polyurethane (CNT‐TPU) foam as the electricity‐generating layer and a PAM/carboxymethyl chitosan (PAM/CMCS) organic‐ionic hydrogel (PO hydrogel) loaded with LiBr as the moisture supply. The porous CNT‐TPU foam promotes water evaporation through capillary action and cation migration via its negative surface charge, generating a potential difference. The PO hydrogel absorbs wound exudate via the hydrophilic PAM, while CMCS offers antimicrobial activity. The top electrode of MEG connects to a Mo stimulation electrode placed on the PAM/CMCS hydrogel dressing in direct contact with the wound for continuous electrostimulation delivery. Despite its advantages, the system faces challenges in maintaining hydrogel stability and consistent MEG output under chronic wounds, where excessive exudate can disrupt function. Furthermore, the need for external adhesives to maintain electrode–tissue contact limits practicality, highlighting the need for integration of bioadhesive or intrinsically adhesive interfaces.

Ionic‐based wound therapies have demonstrated significant potentials to promote cell proliferation and accelerate tissue regeneration, ultimately reducing overall healing time. However, integrating multiple therapeutic systems within a single platform remains a challenge. Moreover, most ionic‐therapeutic systems require voltage sources, adding bulk and discomfort. To overcome this issue, self‐powered systems such as MEGs,^[^ ^530^, ^540^ ^]^ ultrasound‐enhanced piezoelectric nanogenerators,^[^ ^541^ ^]^ thermoelectric hydrogels,^[^ ^542^ ^]^ or charge‐injected hydrogels^[^ ^543^ ^]^ have emerged as promising alternatives. These platforms enable autonomous voltage generation and multi‐functional ion control for enhanced wound healing.

### 5.3 Injectables and Printables

While sheet‐ or patch‐type hydrogel dressings are effective for wound coverage and healing, their limited conformability to complex or irregular tissue surfaces restricts their application.^[^ ^544^ ^]^ To overcome this issue, injectable and printable ionic materials have emerged as promising platforms for next‐generation wound healing and regenerative therapies. Injectable hydrogels can be delivered directly into irregular wound sites, forming intimate contact with surrounding tissues and enabling minimally invasive treatment.^[^ ^545^, ^546^ ^]^ Printable hydrogels, on the other hand, offer precise spatial control for constructing patient‐specific architectures.^[^ ^547^ ^]^ These systems are particularly suitable for applications requiring close integration with complex biological surfaces, thereby enhancing therapeutic efficacy.^[^ ^548^ ^]^ To function effectively in these formats, hydrogels must meet several rheological and mechanical criteria, including appropriate yield stress, shear‐thinning behavior, and extrudability.^[^ ^549^ ^]^ Ionic crosslinkers such as multivalent cations (X^n+^)^[^ ^263^, ^550^, ^551^, ^552^, ^553^ ^]^ and borax ion^[^ ^554^ ^]^ are widely utilized to enhance mechanical properties, and enable in situ injection,^[^ ^96^, ^555^ ^]^ or 3D printing.^[^ ^556^, ^557^, ^558^ ^]^ Beyond structural roles, ions serve as bioactive agents by directly promoting various therapeutic properties, including the antibacterial property of Ag^+^, Zr^4+^, and Fe^3+[^ ^559^, ^560^, ^561^ ^]^ the anti‐inflammatory property of zwitterionic gel,^[^ ^562^ ^]^ and the promotion of differentiation in nerve,^[^ ^563^ ^]^ cell,^[^ ^564^ ^]^ or bone,^[^ ^565^, ^566^, ^567^ ^]^ utilizing Mg^2+^, Si^4+^, and Ca^2+^. Additionally, Mg^2+^ and Si^4+^ promote angiogenesis^[^ ^87^ ^]^ and Mn^2+^ reduces ROS.^[^ ^568^ ^]^ Such ion‐mediated bioactivity enables hydrogels to simultaneously provide mechanical stability and multifunctionality,^[^ ^569^, ^570^, ^571^ ^]^ positioning injectable (**Table** **12**) and printable (**Table** **13**) hydrogels as highly attractive platforms for advanced tissue‐interface applications.

**Table 12:** Injectables.

Functions of ions	Ionic material^a)^	Polymer matrix^b)^	Therapeutic properties derived from ionic material^c)^	Applications	References
Therapeutic properties	Mg^2+^	HA‐Pam	Nerve regeneration	Conduit for peripheral nerve regeneration	[563]
		LL37‐MSC@OCAHM	Promotion of MSCs differentiation	Osteomyelitis therapy	[564]
	Mg^2+^, Si^4+^	GMN	Immunomodulation, angiogenesis, osteogenesis	Vascularized bone regeneration	[87]
	SeO_3_^2−^, Ca^2+^, Mg^2+^, PO_4_^3−^	Ser‐ADH/OCS	SeO_3_^2−^: anti‐tumor activity Ca^2+^, Mg^2+^, PO_4_^3−^: promotion of bone differentiation	Osteosarcoma‐related bone defect repair	[565]
	CBAA	PCBAA	Anti‐inflammatory, anti‐FBR	Continuous subcutaneous insulin infusion (CSII) catheter	[562]
Crosslinking	Fe^3+^	PEDOT:AlgS	‐	Wound monitoring/sealing, hemostasis	[552]
	Borax ion	CS/Gel		Thermoresponsive reversible adhesion for hemostasis	[554]
Therapeutic properties + crosslinking	Ca^2+^, Mn^2+^	Sodium alginate	Ca^2+^: crosslinking, hemostasis, heat concentration Mn^2+^: STING pathway activator	Microwave ablation for tumor therapy	[550]
	Ag^+^	HA‐NCSN	Antibacterial property	Antibacterial dressing	[559]
	Zr^4+^	Gel/EGCG	Antibacterial property	Wound healing	[560]
	Fe^3+^	CPPFe@TA	Photothermal antibacterial property	Wound healing	[561]
	Ag^+^, Zn^2+^	HA‐SH	Ag^+^: antibacterial Zn^2+^: immunomodulatory	Infected wound repair	[569]
	Choline and geranate‐based IL	Gelatin/oxidized dextran	Antibacterial property	Enterocutaneous fistula treatment	[476]

^a)^ CBAA: carboxybetaine acrylamide

^b)^ HA‐Pam: pamidronate‐grafted hyaluronic acid, LL37‐MSC@OCAHM: LL37‐mesenchymal stem cell@aldehyde‐functionalized chondroitin sulfate‐alendronate/hyaluronic acid‐ adipic acid dihydrazide@Mg, GMN: GelMa/MgCO_3_/nanoclay, Ser‐ADH/OCS: Sericin‐grafted by hydrazide bond/oxidized chondroitin sulfate, PCBAA: poly(carboxybetaine acrylamide), CS/Gel: chondroitin sulfate/gelatin, HA‐NCSN: thiourea grafted hyaluronic acid, Gel/EGCG: gelatin/epigallocatechin gallate, CPPFe@TA: hydrogel based on CMC sodium, PEI, PAM, Fe^3+^, and TA

^c)^ FBR: foreign body reaction, STING: stimulation of interferon genes

**Table 13:** Printables.

Functions of ions	Ionic material	Polymer matrix^a)^	Therapeutic properties derived from ionic material	Applications	References
Therapeutic properties	Mg^2+^	GelMa/Car	Cell proliferation, angiogenesis	Genital tract defect repair	[86]
		PLA/aDNA	Self‐neutralization, high mechanical properties, appropriate degradation rate aligned with bone growth	Tumor therapy, tissue regeneration	[567]
	Mn^2+^	GelMA	Reducing ROS	Microenvironment‐activatable visualization and osteogenesis in diabetic bone defect	[568]
	Cu^2+^, Ga^3+^	GelMA/SA	Antibacterial property	Wound Healing	[571]
	Si^4+^, Ca^2+^, Sr^2+^	PCL	Si^4+^, Ca^2+^: accelerating osteogenic differentiation, blood vessel formation Sr^2+^: enhancing polarization of M2 macrophages	Osteochondral defect repair	[566]
Crosslinking	Ca^2+^	Gelatin/gellan gum	‐	Wound healing	[558]
		SA/AM		Repair osteochondral defects in rats	[556]
		Gelatin/SA		Wound Healing	[557]
		SA		Repair infected mandibular defects	[553]
Therapeutic properties + crosslinking	Si^4+^, Ca^2+^	SA	Si^4+^: angiogenic ability, collagen deposition effects Ca^2+^: crosslinking, cationic bridges between DNA‐silica	Diabetic wound healing	[477]
	Na^+^, Ca^2+^	AMPS	Na^+^: enhancing water absorption Ca^2+^: crosslinking, promoting cell growth, antibacterial property, hemostasis	Wound healing	[570]

^a)^ Car: carrageenan, PLA/aDNA: polylactic acid/adhesive DNA, PCL: polycaprolactone, SA: sodium alginate, AM: acrylamide, AMPS: 2‐acrylamide‐2‐methyl‐propanesulfonic acid

**Figure 19:**
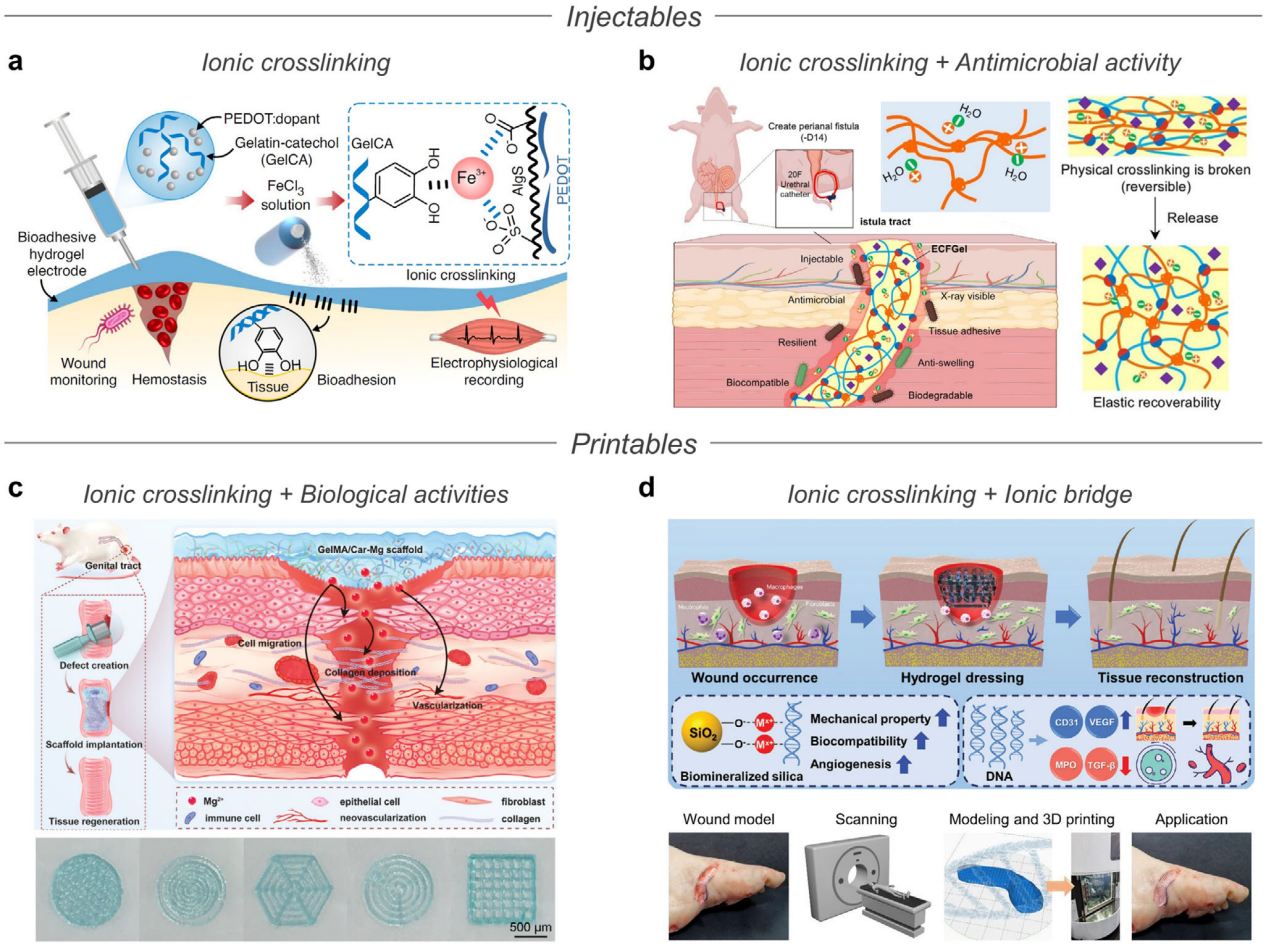
Injectables and printables. a) Fe^3+^ mediate ionic crosslinking of conductive PEDOT:AlgS polymers, enabling their use as conductive inks and bioadhesives for injectable hydrogel bioelectronics. Reproduced with permission.^[^ ^552^ ^]^ Copyright 2025, Springer Nature under CC BY 4.0 license http://creativecommons.org/licenses/by/4.0/. b) Injectable multifunctional hydrogel for occlusion, sterilization, and healing of ECFs, with incorporated IL enhancing mechanical resilience, antimicrobial activity, antiswelling properties, and elastic recoverability. Reproduced with permission.^[^ ^476^ ^]^ Copyright 2025, Wiley‐VCH GmbH. under CC BY 4.0 license http://creativecommons.org/licenses/by/4.0/. c) 3D‐printed dual‐crosslinked bioactive hydrogel scaffold for genital tract defect repair, with Mg^2+^‐mediated ionic crosslinking and tissue regeneration. Reproduced with permission.^[^ ^86^ ^]^ Copyright 2024, Wiley‐VCH GmbH. d) AI‐guided wound imaging and in situ 3D printing of alginate‐based hydrogels for diabetic wound healing, where ionic bridges formed by Na^+^ and Ca^2+^ facilitate DNA‐induced biomineralization. Reproduced with permission.^[^ ^477^ ^]^ Copyright 2023, Wiley‐VCH GmbH. under CC BY 4.0 license http://creativecommons.org/licenses/by/4.0/.

Among injectable hydrogel strategies, ionic crosslinking remains a key approach to achieving mechanical stability and seamless tissue interfacing. Montazerian et al. developed an injectable, water‐dispersible conductive hydrogel by replacing hydrophobic PSS in PEDOT:PSS with hydrophilic sulfonated alginate (AlgS) in the PEDOT:PSS complex (**Figure** **19**a).^[^ ^552^ ^]^ This substitution improved aqueous dispersibility, overcoming the primary challenge of PEDOT hydrogels caused by PSS‐induced aggregation and consequently enhanced the conductivity. The resulting PEDOT:AlgS network formed ionically crosslinkable inks responsive to Fe^3+^ ions through alginate‐mediated coordination,^[^ ^261^ ^]^ leading to rapid gelation not observed in traditional PEDOT:PSS. When combined with catechol‐modified gelatin (GelCA), the resulting hydrogel exhibited minimal swelling, robust tissue adhesion, and hemostatic capability, attributed to the dense ionic network formed by AlgS and Fe^3+^. The hydrogels exhibited a ≈250% improvement in pH sensitivity compared to PEDOT:PSS, likely due to the greater loading of PEDOT moieties within the hydrogel matrix. Overall, the combination of PEDOT:AlgS and ionic crosslinking offers a promising strategy for injectable, biodegradable, and implantable bioelectronics.

Beyond their role in crosslinking, ions can also serve as bioactive agents by being gradually released from hydrogels to exert specific therapeutic effects. Kim et al. developed a multifunctional, tissue‐adhesive injectable hydrogel for the treatment of enterocutaneous fistulas (ECFs) (Figure 19b).^[^ ^476^ ^]^ The hydrogel (ECFgel), composed of gelatin and oxidized dextran (O‐Dex), forms imine bonds with tissue surfaces, enabling strong adhesion. Incorporation of choline and geranate‐based ILs reinforced mechanical properties via the Hofmeister effect,^[^ ^572^ ^]^ enhancing gelation, stiffness, and elasticity without affecting crosslinking. The IL also offered sustained antimicrobial activity, fully eradicating bacteria within four days, and induced antiswelling and contractile behavior by increasing crosslinking density and osmotic pressure. In a porcine perianal fistula model, ECFGel enabled effective tract occlusion, wound contraction, and bacterial clearance, highlighting its potential for treating infected ECFs.

While injectable hydrogels offer simplicity and adaptability for minimally invasive therapies, implantable3D‐printed scaffolds provide mechanical support to maintain structural integrity during tissue regeneration.^[^ ^573^ ^]^ Additionally, in situ 3D printing on biological interfaces enables more precise structural control and personalized regenerative treatments.^[^ ^574^, ^575^ ^]^ To address structural defects such as those associated with Mayer‐Rokitansky‐Küster‐Hauser (MRKH) syndrome, Wang et al. developed a 3D‐printed, dual‐crosslinked hydrogel scaffold combining GelMA and carrageenan (Car) (Figure 19c).^[^ ^86^ ^]^ After room temperature 3D printing, GelMA was UV‐crosslinked while Car was ionically crosslinked with Mg^2+^. The incorporation of Mg^2+^ not only enhanced the mechanical strength and in vivo stability of the hydrogel but also provided continuous Mg^2+^ release for over one month, significantly promoting cell proliferation, migration, collagen deposition, angiogenesis. In a preclinical rat model mimicking vaginoplasty, the GelMA/Car‐Mg scaffold achieved nearly complete wound closure within seven days with minimal scarring. Overall, the integration of Mg^2+^ into printable hydrogels allows ionic crosslinking and sustained bioactivity, offering a promising strategy for personalized and effective regenerative therapies.

Ions not only contribute to crosslinking and therapeutic release but also serve as ionic bridges between negatively charged biomolecules, facilitating structural assembly and biomineralization. Kim et al. developed an artificial intelligence (AI)‐assisted, 3D‐printed hydrogel dressing for diabetic wound healing by integrating marine‐derived biomaterials with a DNA‐induced biomineralization strategy (Figure 19d).^[^ ^477^ ^]^ The hydrogel matrix was based on functionalized sodium alginate (FSA), modified with 3‐glycidoxypropyl trimethoxysilane (GPTMS) to enhance rheological and mechanical properties. Therapeutic bioactivity was imparted by incorporating polydeoxyribonucleotide (PDRN)—a DNA‐derived molecule known for its anti‐inflammatory, pro‐angiogenic, and regenerative effects, along with silica (SiO_2_) nanoparticles (NPs). SiO_2_ NPs reinforced the hydrogel through electrostatic interaction with DNA and released bioactive Si^4+^ ions, promoting angiogenesis and collagen deposition. Ionic bridging by Na^+^ from PDRN and Ca^2+^ reduced electrostatic repulsion between negatively charged DNA and SiO_2_, enabling effective silica biomineralization along the DNA backbone. Furthermore, AI‐driven 3D printing enabled the generation of patient‐specific wound models, allowing for precise in situ fabrication of hydrogel dressings tailored to complex wound geometries. This combination of biomaterial engineering with AI‐guided 3D printing offers a promising approach for personalized treatment of chronic and diabetic wounds.

The development of injectable and printable ionic gels has greatly advanced tissue‐interface applications by enabling conformal wound coverage, mechanical tunability, and enhanced bioactivity through ionic crosslinking and controlled ion release. However, achieving reliable conformability in dynamic in vivo environments remains a key challenge. For injectable systems, granular ionic gels, composed of densely packed microgels, have attracted growing attention due to their shear‐thinning behavior, which facilitates high injectability and microscale porosity, supporting cell migration and infiltration.^[^ ^576^, ^577^ ^]^ Concurrently, increasing focus is being placed on engineering biodegradable ionic gels that can degrade controllably after completing their therapeutic role, ideally breaking down into non‐toxic, bioresorbable components.^[^ ^578^ ^]^ Printable ionic gels, typically used in scaffold‐like structures, should also accommodate dynamic tissue movements to maintain mechanical integrity and intimate tissue contact. As tissue repair progresses, it is also important for the printed gel structure to adapt its morphology^[^ ^579^ ^]^ or gradually disassemble to support regeneration without disrupting natural remodeling processes.^[^ ^580^ ^]^ Given the need for dynamic adaptability in complex biological environments, 4D printing is being actively explored as a novel strategy that combines stimuli‐responsive materials with 3D printing to create structures capable of undergoing programmable shape changes, offering new opportunities for minimally invasive surgical applications.^[^ ^581^, ^582^ ^]^ Moreover, the recent development of deep in vivo sound printing has introduced a new technique, for directly printing hydrogels within living tissues, further expanding the possibilities for minimally invasive interventions.^[^ ^583^ ^]^

### 5.4 Drug Delivery

In addition to wound healing therapies, effective drug delivery systems are crucial for accelerating tissue regeneration by maintaining optimal local drug concentration at the target site. While conventional drug delivery systems, including oral, intravenous, and topical administrations, have been widely used,^[^ ^584^ ^]^ they often lead to systemic drug circulation through the bloodstream, causing off‐target effects and reduced therapeutic efficacy.^[^ ^585^ ^]^ To overcome these limitations, localized delivery strategies for site‐specific drug release are gaining attention, which are vital not only for wound management but also for treating pathological conditions, including cancers^[^ ^586^ ^]^ and tumors,^[^ ^587^ ^]^ as well as for hormone injection therapies.^[^ ^588^ ^]^

For localized drug delivery system, hydrogels are promising platforms due to their ability to encapsulate therapeutic agents^[^ ^589^ ^]^ and provide sustained, controlled release.^[^ ^590^ ^]^ These systems offer several advantages, including biocompatibility,^[^ ^591^ ^]^ tunable structure,^[^ ^490^ ^]^ and high‐water content that facilitate prolonged retention and release of therapeutics.^[^ ^592^, ^593^ ^]^ In addition, they have been engineered to respond to external stimuli, including pressure,^[^ ^594^ ^]^ temperature,^[^ ^595^ ^]^ light,^[^ ^596^ ^]^ and electrical signals,^[^ ^597^ ^]^ enabling on‐demand and site‐specific drug release. Complementing these approaches, ionically mediated delivery systems have garnered attention for their ability to modulate drug release via ionic conductivity, electrostatic interactions,^[^ ^598^ ^]^ and coordination with charged therapeutic agents.^[^ ^599^, ^600^, ^601^ ^]^ Moreover, ILs exhibit unique transdermal permeability, serving as effective carriers for transporting drugs across the skin into subcutaneous tissues.^[^ ^602^, ^603^ ^]^ ion‐based systems have been explored in diverse applications, including skin repair, wound healing,^[^ ^604^, ^605^ ^]^ tumor therapy,^[^ ^606^ ^]^ hair regeneration,^[^ ^166^ ^]^ and implantable drug delivery systems^[^ ^607^ ^]^ (**Table** **14**).

**Table 14:** Drug delivery systems.

Application	Ionic material^a)^	Polymer matrix^b)^	Functions of ions	Drug^c)^	Drug delivery mechanism	References
Skin repair	Ca^2+^/ChCl	HP/AA	Ionic coordination, hydrogen bonding	Flurbiprofen and diclofenac sodium	Zero‐order release	[600]
	LiCl	PAM‐PEGDA	Enhancing hydrogel strength and ionic conductivity	Negatively charged budesonide	Drug release via TENG	[594]
	Choline and geranic acid	PNIPAM/SF/Tween 20	Drug solvent, skin penetration	Methotrexate	Temperature responsive drug release	[603]
Wound healing	SO_4_^2−^, NO_3_^−^	PVA	Ion‐mediated strength transition	Mesenchymal stem cell‐exosomes	Hofmeister‐driven drug release, ion‐responsive hydrogel softening	[604]
	Ce^3+^/Ce^4+^	PU fibers	Metal ion chelation	Bletilla striata polysaccharides	Force‐controlled BCe release	[605]
Tumor treatment	Cu^2+^	BAM15	Ionic coordination	AQ4N	Glutathione‐responsive drug release	[599]
	Positively charged DOX/Mg^2+^	MgSiO_3_ fiber membrane		ICG, DOX	Photothermal and pH induced electrostatic effect	[606]
	DOX	DADMAC/AAm	Charge repulsion and transportation	DOX	Selective ion transport by electric potential	[481]
Hair regeneration	Choline and geranic acid	‐	Drug solvent/API‐ILs	Choline and geranic acid, Minoxidil	ILs‐based drug penetration, API‐ILs	[166]
Implantable system	NaCl/ACh SPA	PSS	Ion transport through ion exchange membrane	ACh	Electrophoretic drug delivery of charged drugs	[598]
	CaCl_2_	Methylcellulose/alginate	Ca^2+^: Methylcellulose thermal gelation, ionic crosslinking Cl^−^: Salting‐out effect	Fenofibrate	Thermoresponsive and ionotropic gelations	[607]

^a)^ DOX: doxorubicin, ACh SPA: poly (acetylcholine 3‐sulfopropyl acrylate)

^b)^ HP: p‐hydroxyphenyl methacrylate, AA: acrylic acid, PNIPAM: poly(isopropylacrylamide), SF: silk fibroin, AMPSA: 2‐acrylamido‐2‐methyl‐1 propanesulfonic acid, BAM15: N5,N6‐bis(2‐Fluorophenyl) [1,2,5]oxadiazolo [3,4‐b]pyrazine‐5,6‐diamine

^c)^ AQ4N: 1,4‐bis([2‐(dimethylamino‐N‐oxide)ethyl]amino)5,8‐dihydroxy‐ anthracene‐9,10‐dione, ICG: indocyanine green

^d)^ BCe: bletilla striata polysaccharide‐confined nano cerium oxide

**Figure 20:**
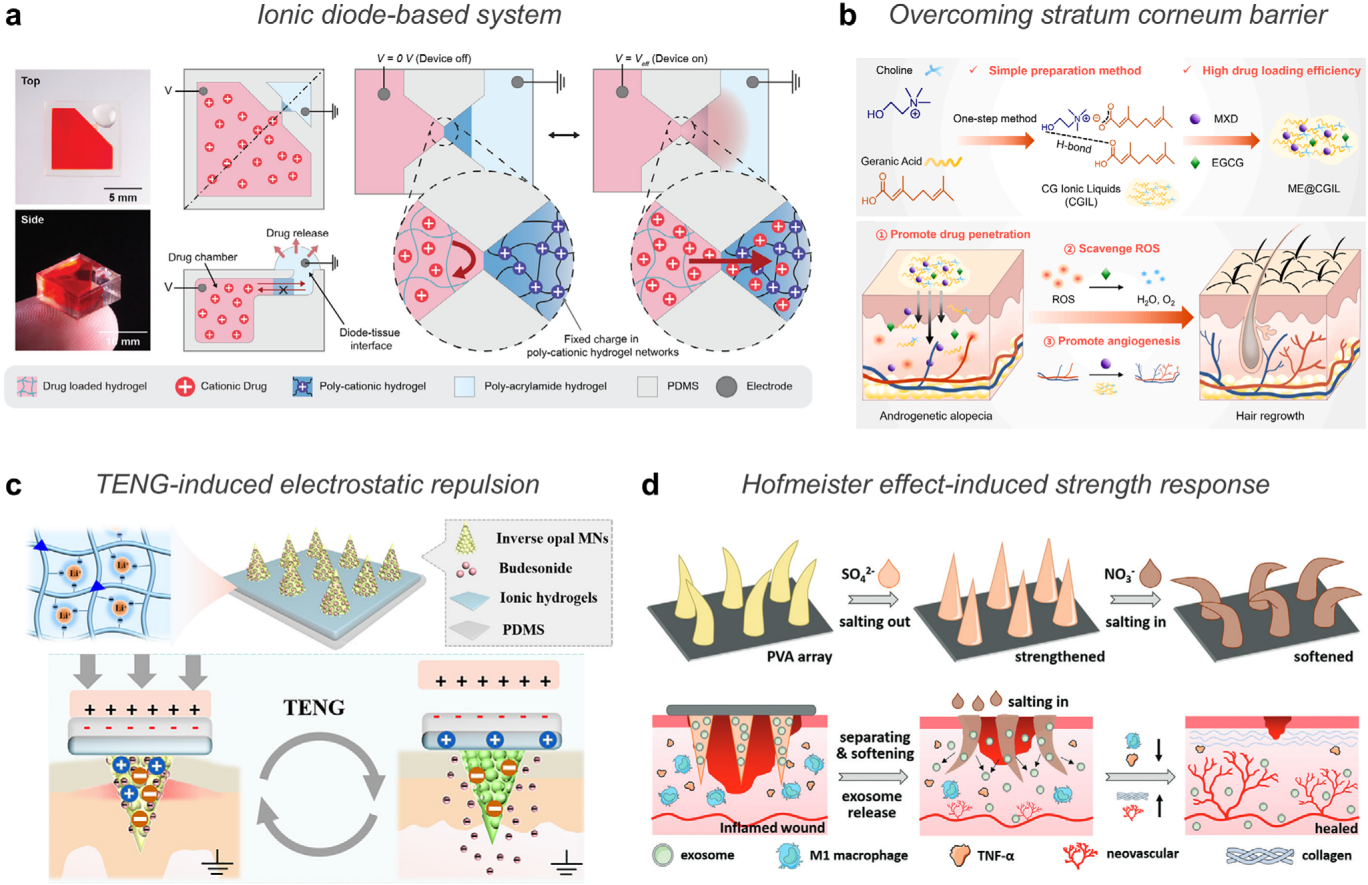
Drug delivery systems. a) Flow‐free, spatiotemporally controlled, and implantable ionic diode‐based drug delivery system. Reproduced with permission.^[^ ^481^ ^]^ Copyright 2025, Wiley‐VCH GmbH. b) IL‐mediated topical delivery of drugs for treating androgenic alopecia. Reproduced with permission.^[^ ^166^ ^]^ Copyright 2024, Elsevier. c) Triboelectric MN patch for psoriasis treatment. Reproduced with permission.^[^ ^594^ ^]^ Copyright 2023, Elsevier. d) Bioinspired adaptable indwelling MNs mediating strength response by ions. Reproduced with permission.^[^ ^604^ ^]^ Copyright 2023, Wiley‐VCH GmbH.

Ionic diode systems, composed of oppositely charged polymers, enable selective and unidirectional transport of charged carriers under electric field.^[^ ^608^ ^]^ When integrated into drug delivery systems, they offer a promising strategy for achieving controlled release with electrically tunable on‐off switching functionality. Demonstrating this potential, Yoo et al. developed an ionic diode‐based drug delivery system (IDDS) for on‐demand drug release for tumor removal applications (**Figure** **20**a).^[^ ^481^ ^]^ This system employs a poly‐cationic hydrogel composed of diallyldimethylammonium chloride (DADMAC) and acrylamide (AAm) to selectively regulate the cationic drug (doxorubicin) delivery. In the off‐state, electrostatic repulsion between the cationic drug and polycationic hydrogel prevents drug diffusion toward the target site. Upon application of an electrical potential (on‐state), the electric field overcomes the ionic barrier, driving the drug release to the target. Encapsulated in biocompatible PDMS, the soft IDDS is suitable for subcutaneous implantation. The platform offers flow‐free and sustained drug delivery platform for on‐demand implantable therapy but requires external voltage and is limited to single‐charged, ion‐specific drugs, restricting broader application.

Conventional drug delivery systems often rely on implantation of drug reservoirs or adhesive patches,^[^ ^609^ ^]^ both of which are limited by bulky form factors or poor skin penetration, leading to suboptimal therapeutic efficiency.^[^ ^610^, ^611^ ^]^ In contrast, ILs have gained attention for their ability to overcome the stratum corneum (SC) barrier, enhancing transdermal transport of active drug ingredients.^[^ ^480^ ^]^ Additionally, ILs enhance the solubility of water‐insoluble drugs, increasing their bioavailability and expanding their utility for non‐aqueous drug delivery system.^[^ ^162^ ^]^ Notably, choline and geranin acid ILs (CGIL) enhance permeability by interacting with SC lipids, making them effective transdermal delivery agents.^[^ ^602^ ^]^ Luo et al. developed a multifunctional CGIL‐based formulation for the co‐delivery of minoxidil (MXD) and epigallocatechin gallate (EGCG), termed ME@CGIL (Figure 20b),^[^ ^166^ ^]^ to treat androgenetic alopecia (AGA), a progressive hair loss disorder driven by oxidative stress and ROS‐induced follicle damage.^[^ ^612^, ^613^ ^]^ MXD promotes follicular activity, while EGCG acts as a ROS scavenger. CGIL, formed via ionic and hydrogen bonding, functions as both a hair regrowing factor and solvent for MXD and EGCG, while also enhancing skin penetration and retention time of drugs. Despite its efficacy, the liquid‐phase nature of IL system limits long‐term stability and sustained release, requiring an encapsulating matrix to maintain drug levels.^[^ ^614^ ^]^ To address these limitations, incorporating biocompatible polymer matrices offers a promising strategy to transform IL‐based systems into stable solid‐phase formulations with sustained drug release capabilities.

Microneedle (MN) patches offer a minimally invasive approach to bypass the SC barrier and create microchannels for transdermal drug transport.^[^ ^615^ ^]^ However, conventional MN systems often lack precise drug release, resulting in unpredictable dose. To address this limitation, Wang et al. developed a self‐powered MN patch integrated with a TENG for electrically mediated, tunable drug release (Figure 20c).^[^ ^594^ ^]^ The MN patch consists of a PAM‐PEGDA‐LiCl ionic hydrogels with budesonide, a psoriasis drug, loaded into the MN tips. Li^+^ enhances mechanical strength of hydrogel through hydrogen bonding with free water and improve conductivity, both essential for efficient electrostimulation. Encapsulated with negatively charged PDMS, triboelectric contact with skin drives ionic redistribution, causing Li^+^ accumulation and anion retention that electrostatically repels and releases the negatively charged budesonide. This self‐powered, ion‐mediated platform enables contact‐dependent, precisely controlled drug release, offering superior performance over passive MN systems.^[^ ^612^, ^616^ ^]^ However, a mechanical mismatch between the rigid MNs and soft tissues may induce local stress or cellular damage.^[^ ^604^ ^]^ Tuning the modulus for tissue compatibility and ensuring biocompatible degradation are essential for safe and effective drug delivery.

Dynamic modulation of mechanical strength is critical for MN patches to adapt to soft tissues and minimize damage. Zhang et al. developed an adaptable indwelling MN system based on PVA hydrogel loaded with mesenchymal stem cell (MSC)‐derived exosomes for diabetic ulcer treatment (Figure 20d).^[^ ^604^ ^]^ The system leverages Hofmeister effects, an ion‐specific phenomenon altering the solubility and stability of polymers,^[^ ^617^ ^]^ to achieve ion‐responsive tuning of mechanical strength of PVA hydrogel. Sulfate ions (SO_4_^2−^), from Na_2_SO_4_, strengthen PVA needle tips by inducing water expulsion and promoting chain aggregation and crystallization, supporting SC penetration and stable MSC‐exosome loading. After insertion, nitrate ions (NO_3_^−^), delivered through Fe(NO_3_)_3_ treatment, disrupt the PVA crystalline matrix, softening the tips to adapt to tissue and facilitate continuous exosome release for improved wound healing and regeneration. While the system employs innovative ion‐mediated Hofmeister effects to dynamically modulate the mechanical properties of MN tips, the potential cytotoxicity from Na_2_SO_4_ and Fe(NO_3_)_3_ necessitates precise concentration control for safe clinical application.

Ion‐mediated drug delivery systems allow precise dosing and transdermal transport, offering substantial therapeutic potential. However, material‐specific limitations remain, as hydrogels are generally suitable for water‐soluble drugs, while IL‐based systems better deliver water‐insoluble components, requiring careful material selection. For long‐term clinical use, fully biodegradable systems that degrade completely in vivo without residual toxicity are essential.^[^ ^618^, ^619^ ^]^ Incorporating mechanically tunable materials with biodegradable systems can enhance cytocompatibility and support adaptability to diverse tissues, making these platforms more effective and patient‐friendly for personalized therapies.

### 5.5 Neural and Cardiac Stimulation

Electrical modulation of excitable tissues restores or adjusts physiological functions by delivering controlled electrical signals to targeted tissues with disrupted or weak bioelectrical activity.^[^ ^620^ ^]^ Peripheral nerve stimulation activates specific neural pathways to alleviate chronic pain or restore motor function in conditions such as spinal cord injury or neuropathy.^[^ ^621^ ^]^ Cardiac stimulation delivers timed impulses to the myocardium to maintain rhythmic contractions, support blood circulation, and prevent arrhythmias.^[^ ^622^ ^]^ Traditional approaches rely on metallic electrodes, such as platinum or iridium, to deliver pulsed electrical currents via capacitive or faradaic charge transfer.^[^ ^623^ ^]^ While effective, such systems often suffer from mechanical mismatch with soft neural tissues, leading to high interfacial impedance or foreign body responses.^[^ ^624^ ^]^ In contrast, iontronic devices that employ ionic materials, such as OMIECs,^[^ ^196^, ^197^, ^219^, ^625^, ^626^, ^627^ ^]^ hydrogels,^[^ ^546^, ^628^, ^629^, ^630^, ^631^ ^]^ or ion gels,^[^ ^41^, ^174^ ^]^ offer a more biologically relevant mode of signal delivery (**Table** **15**). By conducting or modulating ions directly, these materials provide soft neural coupling and precise charge delivery, minimizing tissue damage.^[^ ^316^ ^]^ Moreover, their tunable ionic conductivity^[^ ^632^ ^]^ and hydration compatibility^[^ ^633^ ^]^ further support conformal, high‐resolution interfaces for bidirectional communication with biological systems.

**Table 15:** Neural and cardiac stimulation systems.

Target	Ionic material^a)^	Polymer matrix	Properties	Applications	References
Peripheral nerve	PEDOT:PSS	PEDOT:PSS	Laser‐induced phase separation	Sciatic nerve stimulation	[196]
	PEDOT:PSS	PAA/PEDOT:PSS	Enhanced fiber network orders via template‐directed assembly		[219]
	PEDOT:PSS, EMIM^+^, TCB^−^	PEDOT:PSS	3D printing with colloidal ink		[197]
	Na^+^ (SEBS‐SN), Cl^−^ (SEBS‐IM)	SEBS‐SN/SEBS‐IM	Fiber‐shaped ionic heterojunction enabling EDL‐driven ion rectification	In vivo sciatic nerve stimulation & peripheral nerve injuries rehabilitation via end‐to‐side anastomosis	[638]
	Ch^+^, MA^−^	Genipin‐crosslinked chitosan	Non‐faradaic (high EDL capacitance)	Nerve stimulation for overactive bladder control	[41]
	Ch^+^, MA^−^	GelMA			[174]
	EMIM^+^, Cl^−^	PAA/PASP‐DA	Tunable adhesion	Electrodiagnostic monitoring for peripheral neuropathy	[631]
Heart	Li^+^	Recombinant silk‐based hydrogel	UV‐activated soft Li‐ion battery	Electrical stimulation of ex vivo rat heart	[630]
	ETE‐BuSA	Variant of PEDOT:S	Bioresorbable	Heartbeat regulation, arrhythmia rectification	[625]
	NM, SBMA	PEDOT:PSS	Fully implantable and wireless	Cardiac pacing	[626]
	PEDOT:PSS	PEDOT:PSS hydrogel	Low impedance		[314]
Neuron	Ca^2+^, Cl^−^	Agarose hydrogel shell PAH (anion‐selective), PSS (cation‐selective)	Droplet‐based ionic voltage generator enabling autonomous stimulation	Battery‐free modulation of neuronal activity in mouse brain slices	[628]
	PEDOT:PSS	PLGA/PEDOT:PSS	Biodegradability	Brain neural precursor cell activation	[627]

^a)^ SEBS‐SN: SEBS grafting sodium sulfonate groups, SEBS‐IM: SEBS grafting 3‐hexylimidazolium groups, ETE‐BuSA: 8‐(2‐(2,5‐bis(2,3‐dihydrothieno[3,4‐b][1,4]dioxin‐5‐yl)thiophen‐3‐yl)ethoxy)−1‐(trimethylammonio)octane‐4‐sulfonate, NM: neostigmine methanesulphate, SBMA: [2‐(methacryloyloxy) ethyl] dimethyl‐(3‐sulfopropyl) ammonium hydroxide

^b)^ PASP‐DA: poly(aspartic acid) grafted with dopamine, PAH: poly(allylamine hydrochloride), PLGA: poly(lactic‐co‐glycolic) acid

**Figure 21:**
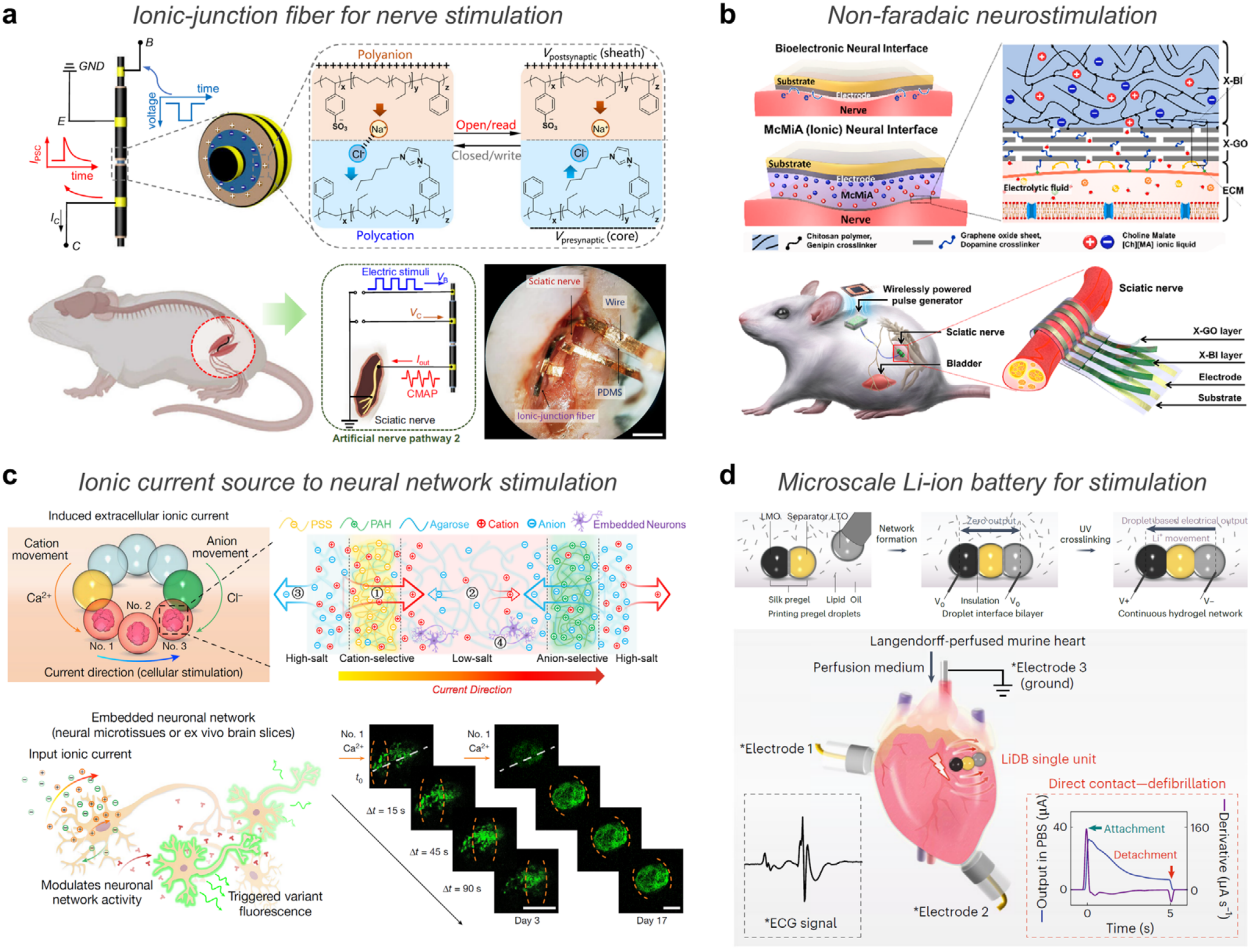
Ionic systems for neural and cardiac stimulation. a) Fiber‐shaped ionic heterojunction for seamless and conformal nerve stimulation. Reproduced with permission.^[^ ^638^ ^]^ Copyright 2023, Springer Nature under CC BY 4.0 license http://creativecommons.org/licenses/by/4.0/. b) Ionic neural interfaces for non‐faradaic neurostimulation. Reproduced with permission.^[^ ^41^ ^]^ Copyright 2023, American Chemical Society. c) Hydrogel droplet‐based ionic current source for stimulating neural network activity. Reproduced with permission.^[^ ^628^ ^]^ Copyright 2023, Springer Nature under CC BY 4.0 license http://creativecommons.org/licenses/by/4.0/. d) Microscale Li‐ion battery for cardiac rhythm modulation. Reproduced with permission.^[^ ^630^ ^]^ Copyright 2024, Springer Nature under CC BY 4.0 license http://creativecommons.org/licenses/by/4.0/.

Ionic heterojunction devices, composed of p‐ and n‐type ionic layers, can rectify and switch ionic currents, enabling direct biosignal communication^[^ ^12^ ^]^ and bio‐mimetic signal modulation.^[^ ^634^ ^]^ However, most ionic heterojunction devices rely on 2D^[^ ^234^ ^]^ or 3D^[^ ^481^ ^]^ structures, limiting integration with fiber‐shaped tissues due to structural mismatch. Meanwhile, fiber‐shaped iontronic devices offer nerve‐like morphology and mechanical compatibility for seamless implantation.^[^ ^635^, ^636^ ^]^ Nonetheless, incorporating ionic heterojunctions into 1D structures is challenging due to difficulties in multilayer alignment and interface control.^[^ ^637^ ^]^ Xing et al. addressed these limitations by fabricating an ionic heterojunction fiber using an integrated opposite charge grafting (IOCG) strategy (**Figure** **21**a).^[^ ^638^ ^]^ Oppositely charged functional groups were sequentially grafted onto a common poly(styrene‐b‐(ethylene‐co‐butylene)‐b‐styrene) (SEBS) precursor: 3‐hexylimidazolium groups for a cationic layer (SEBS‐IM) and sulfonate groups via poly(sodium 4‐styrenesulfonate) (PSSNa) for an anionic counterpart (SEBS‐SN), incorporating Na^+^ and Cl^−^ as mobile ions. These ion‐selective polymers were coated layer‐by‐layer onto a CNT fiber core, forming a continuous ionic heterojunction along the fiber axis, followed by CNT spray coating on the outer anionic layer to form a CNT sheath. This coaxial configuration provides ionic rectification behavior via ionic double layer formation, while maintaining high flexibility and durability. This work demonstrates a structurally integrated and biocompatible fiber‐based ionic junction structure capable of localized neuromodulation, offering significant potential for seamless soft neural interface applications.

The neural interface plays a critical role in determining the stimulation efficiency of neural devices. Since electric fields diminish rapidly with distance between the electrode and tissue, maintaining conformal contact is essential. Conventional metal electrodes poften suffer from mechanical mismatch, leading to interfacial defects or tissue damage.^[^ ^639^ ^]^ Moreover, the aqueous nature of physiological environments limits the electrochemical window, increasing the risk of irreversible Faradaic reactions, including water electrolysis,^[^ ^640^ ^]^ electrode oxidation,^[^ ^322^ ^]^ and gas formation,^[^ ^641^ ^]^ when using electron‐conductive materials such as graphene or CNTs, thereby compromising long‐term biocompatibility. Even in ion‐conductive electrodes, ion exchange at the electrode–tissue interface may disturb electrolyte balance, potentially leading to blood pressure fluctuations or nervous system disorders.^[^ ^642^ ^]^ To overcome these issues, Kim et al. developed a biocompatible, multi‐crosslinked membrane‐ionogel (McMiA) for conformal neural interfacing (Figure 21b).^[^ ^41^ ^]^ McMiA enables non‐faradaic capacitive stimulation while minimizing chemical and mechanical mismatches. McMiA comprises two parts: a genipin‐crosslinked chitosan‐based ionogel with choline malate ([Ch][MA]) IL, referred to as X‐BI, for ionic conductivity and mechanical compliance, and a dopamine‐crosslinked graphene oxide membrane (X‐GO) that acts as an ion‐selective barrier, blocking biological ion exchange and synthetic ion leakage into surrounding tissue. Together, these layers create a bioadhesive, flexible, and electrochemically stable interface. This work highlights the potential of ion gel as a stable, conformal, and biocompatible neural interface that avoids the adverse effects in neurostimulation associated with faradaic charge injection.

While previous neural stimulation studies have primarily focused on device architecture and interfacial stability, the realization of fully bio‐integrated systems ultimately requires a reliable power source.^[^ ^643^ ^]^ Traditional approaches, including wired power delivery or wireless energy transmission,^[^ ^644^, ^645^ ^]^ are effective for large‐scale systems, but face limitations at the microscale. Even wireless systems typically require implanted pulse generators, hindering miniaturization, flexibility, and long‐term biocompatibility.^[^ ^41^ ^]^ To overcome these limitations, Zhang et al. demonstrated a miniaturized soft power source capable of generating ionic current for biological stimulation (Figure 21c).^[^ ^628^ ^]^ The device consists of hydrogel droplets, each with a diameter of several hundred micrometers, made from low‐gelling‐temperature (LGT) agarose. These droplets are arranged in sequence: a high‐salt droplet, a cation‐selective layer PSSNa), a low‐salt droplet, an anion‐selective layer (poly(allylamine hydrochloride) (PAH)), and a second high‐salt droplet, with calcium chloride (CaCl_2_) used as the ionic component. Upon lipid bilayer removal and thermal gelation, a continuous hydrogel structure forms, allowing spontaneous ion diffusion across the droplets. This initiates an internal ionic current via a reverse electrodialysis‐like mechanism, yielding a power density of ≈1300 W/m^3^. These findings present a compact, biocompatible, and flexible power platform for microscale neuromodulation, integrating ionic energy generation and localized stimulation to advance autonomous, minimally invasive bio‐integrated devices.

Despite progress in biocompatible microscale stimulation, the salt‐based hydrogel droplet platforms face several limitations, including salt‐gradient‐dependent low output, limited rechargeability compared to Li‐ion batteries, and complex activation procedures involving temperature‐sensitive gelation and oil‐based buffer exchange, hindering practical biomedical integration. Although effective at the microtissue level, such systems require further development to achieve organ‐level stimulation, which demands higher intensity and sustained power delivery for full physiological responses.^[^ ^646^ ^]^ To overcome these limitations, Zhang et al. incorporated a soft, rechargeable Li‐ion droplet battery integrated within the same lipid–droplet–hydrogel framework, enabling autonomous tissue stimulation (Figure 21d).^[^ ^630^ ^]^ The Li‐ion droplet battery (LiDB) consists of a cathode droplet containing lithium manganese oxide (LiMn_2_O_4_) and CNTs, an anode droplet containing lithium titanate (Li_4_Ti_5_O_12_) and CNTs, and a separator droplet with lithium chloride (LiCl). Each droplet is coated with a lipid monolayer that forms a droplet interface bilayer (DIB) upon contact, preventing premature ion mixing. Upon UV‐induced dityrosine crosslinking of silk fibroin, the DIBs rupture, initiating Li^+^ conduction across the hydrogel network. Once activated, the LiDB provides stable electrochemical performance, with a volumetric capacity of 570 nAh/µL, an average discharge voltage of 0.65 V, and a power density of ≈10 µW/cm^2^. Multiple units can be connected in series to generate higher voltages (≈3.3 V), sufficient to power LEDs and small electronic devices. The soft, biodegradable silk hydrogel ensures conformal contact with biological tissues, allowing direct application of the LiDB onto ex vivo mouse hearts. It delivered a low‐energy electrical shock (≈30 µA) to restore normal heart rhythms after chemically induced arrhythmia. Overall, this work introduces a fully soft, rechargeable, and biodegradable power source capable of directly stimulating tissue, making it highly suitable for implantable biomedical applications. Nonetheless, for systems that require repeated or long‐term operation, improving in vivo rechargeability remains a key challenge.

Ionic materials and iontronic devices offer versatile platforms for neuromodulation and cardiac stimulation, including fiber‐shaped devices for seamless nerve integration, ion gels with wide electrochemical windows for safe stimulation, and droplet‐based ionic gels serving as miniaturized power sources. Although these advances are promising, the inherently low charge density of ionic systems compared to metallic electrodes reduces the efficiency of charge injection needed for reliable and localized tissue stimulation.^[^ ^184^ ^]^ Enhancing electroactive surface area through structural optimization^[^ ^647^, ^648^ ^]^ or using conductive polymers like PEDOT:PSS^[^ ^649^, ^650^ ^]^ and incorporating conductive fillers^[^ ^651^ ^]^ offers a viable strategy to overcome this limitation.

## 6 Conclusions and Future Directions

### 6.1 Research Highlights in Iontronic Bioelectronics

Iontronic bioelectronics presents a powerful strategy to bridge the longstanding gap between traditional electronics and biological systems, which inherently rely on ionic mechanisms for signal transmission and physiological regulation. Traditional bioelectronic devices, primarily based on electron transport and rigid substrates, struggle to interface effectively with soft, hydrated, and ion‐mediated biological tissues. In contrast, iontronic systems incorporate mobile ions within mechanically compliant, soft matrices, enabling the formation of intimate, low‐impedance interfaces with living tissues. These ion‐based systems facilitate the accurate and stable detection of electrophysiological signals, vital signs, and biomarkers. Moreover, their mechanical compliance and tunable ionic conductivity make them particularly well‐suited for integration with soft, dynamic biological environments.

Importantly, iontronic materials extend beyond passive sensing. A diverse range of ionic species, including metal ions, zwitterions, and ILs, exhibits therapeutic functionalities. Metal ions and zwitterionic compounds exhibit intrinsic antibacterial, anti‐inflammatory, and regenerative effects, while ILs function either as drug carriers or as therapeutic agents. These ionic functionalities play essential roles in a wide range of therapeutic applications, including wound healing, tissue repair, and targeted antitumor therapy. In parallel, iontronic devices have demonstrated powerful capabilities in therapeutic modulation, particularly in neural and cardiac electrostimulation, real‐time wound monitoring, and on‐demand drug release, extending their utility across both therapeutic and diagnostic domains. Emerging platforms, such as ionic heterojunction fibers, ion gels with wide electrochemical windows, and droplet‐based ionic generators, illustrate the potential of iontronic systems to serve as both stimulus‐responsive interfaces and miniaturized energy sources for autonomous operation.

These developments position ions not merely as passive charge carriers but as active biofunctional elements capable of sensing, responding to, and modulating physiological processes in real time. Through their dynamic interactions with biological environments, ranging from ionic signalling and charge transfer to bioactive interactions and therapeutic release, ions serve as integrated components in both diagnostics and therapy. This dual sensing–therapeutic capability of ionic–bionic interfaces expands the scope of bioelectronic applications, enabling seamless communication between artificial systems and living tissues. Furthermore, it paves the way for the development of adaptive, multifunctional, and personalized healthcare technologies that can intelligently interface with the human body to monitor, predict, and treat physiological conditions with high specificity and minimal invasiveness.

### 6.2 Challenges and Future Directions

**Figure 22:**
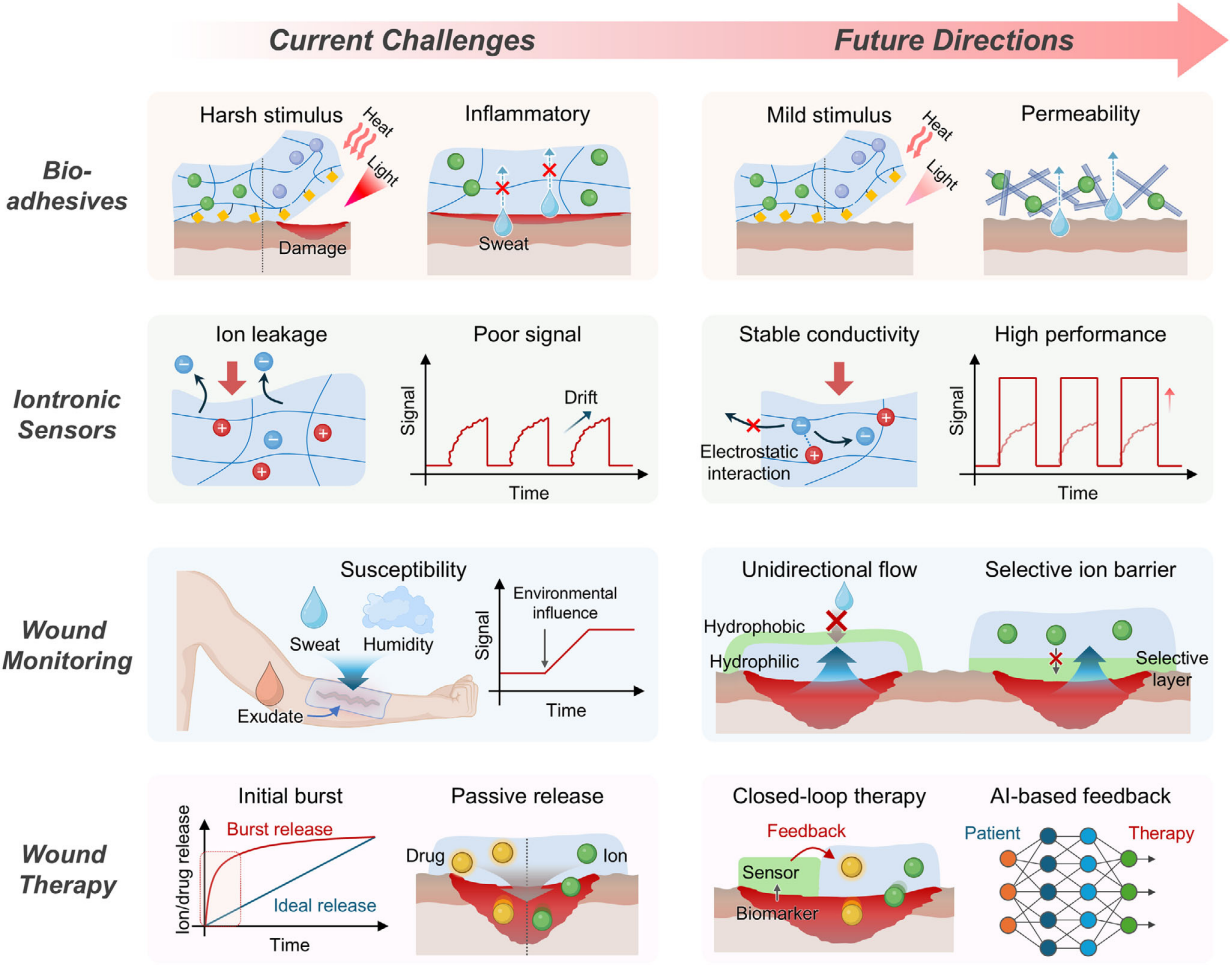
Current challenges and future directions in bioadhesives, iontronic sensors, wound monitoring, and wound therapy. Advancements focus on improving adhesion, signal stability and performance, environmental resilience in wound monitoring, and transitioning from passive to sensor‐ or AI‐assisted closed‐loop drug delivery.

Although iontronic bioelectronics have made rapid advancements, several important challenges remain to be addressed. As illustrated in **Figure** **22**, current limitations span across four key domains: 1) bioadhesives, which still rely on harsh stimuli and often suffer from poor breathability or inflammatory responses; 2) iontronic sensors, which face issues such as ion leakage and signal instability; 3) wound monitoring systems, which struggle with signal interference from sweat, exudate, and humidity; and 4) wound therapy platforms, which are limited by uncontrolled ion release and passive drug delivery. Addressing these challenges will be essential to developing robust, adaptive, and clinically viable iontronic systems. Emerging strategies, including mild‐stimulus‐responsive adhesives, high‐fidelity sensing architectures, selective ion barriers for wound monitoring, and closed‐loop AI‐guided therapeutic platforms, are paving the way toward next‐generation bioelectronics. In practical settings. The following subsections 6.2.1–6.2.4 examine each of these challenges in detail and propose material, structural, and system‐level innovations aimed at realizing stable, responsive, and application‐ready iontronic bioelectronic platforms.

#### 6.2.1 Bioadhesives: Reversibility, Clinical Compatibility, and Breathability

Robust integration with biological tissues is indispensable not only for the stable operation of bioelectronic devices, but also for broader applications in surgical adhesives and tissue repair. In addition to strong and biocompatible adhesion, reversible attachment is often required in surgical applications that involve device repositioning or removal. Various strategies have been developed to modulate adhesion strength, including reversible coordination with metallic ions,^[^ ^287^, ^652^ ^]^ and external stimuli such as heat,^[^ ^274^ ^]^ or ultraviolet (UV) light.^[^ ^35^, ^653^ ^]^ While these strategies offer effective tuning of adhesion strength, their clinical applicability remains limited due to the often harsh and non‐physiological conditions of the stimuli required to modulate adhesion strengths.^[^ ^654^ ^]^ To minimize potential damage to fragile skin or internal organs, future research should focus on designing highly sensitive, ionically responsive materials that reduce the activation threshold and respond to mild stimulus. In addition, expanding the range of external stimuli explored is important. For example, replacing conventional high‐intensity UV light with visible or near‐infrared (NIR) light offers a more clinically favorable alternative.

In parallel, wearable and epidermal applications of ionic bioadhesives present distinct challenges related to long‐term use and skin comfort. Conventional ionic bioadhesives and epidermal electrodes are often fabricated as thick and densely crosslinked polymer matrices with limited moisture permeability, leading to skin irritation and discomfort during prolonged wear. Skin naturally requires a breathable microenvironment, with adequate air and moisture exchange to maintain homeostasis.^[^ ^655^ ^]^ While hydrophilic hydrogel matrices can absorb sweat, they often induce undesired swelling, leading to deteriorated adhesive strength or ionic conductivity.^[^ ^656^ ^]^ To overcome these limitations, next‐generation ionic bioadhesives and epidermal electrodes must combine robust adhesion with enhanced breathability. Structural approaches such as ultrathin, porous, or fibrous architectures offer promising routes to facilitate effective sweat evaporation, while maintaining conformal contact and stable physiological signal acquisition.^[^ ^657^ ^]^ A key challenge is to design breathable ionic networks that enable controlled ion transport without compromising adhesion performance or conductivity, particularly for applications requiring continuous wear and long‐term reliability.

#### 6.2.2 Iontronic Sensors: Long‐Term Stability and Signal Fidelity

Long‐term operational stability is critical for iontronic sensors, which are inherently prone to performance degradation due to the dynamic behavior of mobile ions. As iontronic sensors are increasingly expected to perform reliably not only in normal conditions but also under challenging environments such as mechanical deformation, underwater or wide temperature variations, ensuring robust and stable performance becomes essential. Among the key challenges, ion leakage and signal drift have long hindered operational stability. To suppress ion leakage, zwitterionic polymers^[^ ^658^ ^]^ or polyelectrolytes^[^ ^144^, ^368^, ^659^ ^]^ have been introduced, which immobilize mobile ions by binding them to polymer chains. Signal drift is mitigated by applying creep‐free layers atop these leakage‐suppressing layers.^[^ ^144^, ^368^ ^]^ In parallel, motion‐induced artifacts are reduced by engineering strain‐insensitive pressure sensors using multilayer architectures with modulus gradients,^[^ ^25^ ^]^ while cross‐talk is minimized by structurally isolating ionic sensing elements.^[^ ^660^ ^]^ For robust operation in harsh environments, such as underwater and extreme temperature conditions, self‐healing ionic elastomers employing hydrogen bonding^[^ ^661^ ^]^ or metal ion coordination^[^ ^662^ ^]^ have been developed. Integrating these material and structural strategies into a unified platform offers a promising pathway to achieve high signal fidelity and environmental robustness. As stability improves, it is equally important to ensure that sensor performance, such as sensitivity, resolution, and response time, is maintained, as trade‐offs can occur. While recent efforts have markedly improved the stability of iontronic pressure sensors, progress in iontronic temperature sensing remains limited, despite its essential role in complementary vital sign monitoring. Therefore, systematic strategies are needed to ensure reliable, multimodal sensing performance across various iontronic sensor types.

#### 6.2.3 Wound Monitoring: Ionic Signal Specificity and Environmental Robustness

Ionic sensing systems offer strong potential for real‐time monitoring of wound biomarkers such as pH and temperature, enabling sensitive detection of physiological changes associated with healing progression or bacterial infection. Despite this promise, ion‐based wound monitoring remains underdeveloped, largely due to challenges in maintaining stable and selective responses in complex, dynamic environments influenced by sweat,^[^ ^663^ ^]^ exudate,^[^ ^664^ ^]^ and ambient humidity.^[^ ^665^ ^]^ These external factors can interfere with ionic responses, leading to reduced specificity, signal drift, and long‐term instability. Despite these challenges, ion‐mediated sensing platforms offers key advantages, including direct responsiveness to biochemical cues and seamless integration with therapeutic functionalities. Future efforts should focus on enhancing biomarker specificity under biofluid‐rich conditions to ensure stable and reliable sensing. To address these limitations, structural design strategies can be employed, such as (i) the introduction of unidirectional fluid transport layers^[^ ^666^ ^]^ to restrict back‐diffusion and minimize direct contact between the sensing layer and the wound bed, and (ii) the incorporation of ion‐selective barriers^[^ ^667^ ^]^ to stabilize local ion concentrations within the active layer. By implementing these strategies, it may be possible to develop intelligent ionic wound dressings capable of long‐term, high‐fidelity monitoring under realistic physiological conditions, while preserving compatibility with therapeutic functions.

#### 6.2.4 Wound Therapy: Closed‐Loop Control through Sensing and AI Integration

Effective chronic wound care relies on dressings that are stable over extended periods, minimize replacement frequency, and incorporate therapeutic functions such as biosensing, controlled drug delivery, and antimicrobial activity. To meet these multifunctional demands, ionic materials have been increasingly incorporated into dressing matrices to improve mechanical robustness and serve as carriers for sustained drug and ion release. Particularly, controlled release of multiple ions has gained attention, as distinct ions play complementary roles across different stages of healing.^[^ ^668^ ^]^ However, conventional systems often suffer from initial burst drug release, which may compromise therapeutic efficacy and cause adverse effects,^[^ ^669^ ^]^ as well as uncontrolled ion diffusion, especially at high concentrations of metal ions, increases the risk of cytotoxicity.^[^ ^109^ ^]^ Although regulating ion concentration and tailoring drug release in response to wound conditions could enhance treatment precision, practical implementation remains complex due to individual tissue variability and the reliance on external stimuli for control.

Recent advances have focused on sequential ion release systems that allow temporal control of therapeutic delivery without relying on external triggers. For example, sequential ion release has been achieved using degradable bilayer hydrogels,^[^ ^670^ ^]^ hydrogel systems embedded with metal‐organic frameworks (MOFs)^[^ ^671^ ^]^ or microbeads,^[^ ^668^ ^]^ where multiple ion carriers degrade at distinct stages to enable phased delivery aligned with key healing processes such as inflammation, angiogenesis, and tissue regeneration. These passive strategies mimic natural healing cascades and reduce cytotoxic risk, but they remain limited by fixed release kinetics that cannot respond to patient‐specific wound dynamics.

To move beyond the limitations of fixed, non‐responsive delivery systems, smart closed‐loop platforms are gaining attention for their ability to autonomously modulate drug release based on real‐time physiological feedback.^[^ ^597^, ^672^, ^673^ ^]^ Unlike passive systems that follow predetermined release profiles regardless of wound dynamics, closed‐loop strategies can incorporate sensing components to monitor parameters such as pH, temperature, and inflammatory markers, enabling adaptive drug and ion release through feedback regulation. These sensor‐integrated systems enable dynamic, on‐demand treatment tailored to individual wound environments. Furthermore, integrating AI‐based feedback algorithms enables data‐driven design, predictive modeling, and optimization of hydrogel formulations to achieve controlled and personalized release profiles.^[^ ^674^ ^]^ By responding precisely to actual physiological needs, AI‐guided and sensing‐enabled dressings offer a highly adaptive and personalized therapeutic approach that surpasses conventional passive delivery systems.

## Conflict of Interest

The authors declare no conflict of interest.
